# Genome-wide gene-environment interactions in neuroticism: an exploratory study across 25 environments

**DOI:** 10.1038/s41398-021-01288-9

**Published:** 2021-03-22

**Authors:** Josefin Werme, Sophie van der Sluis, Danielle Posthuma, Christiaan A. de Leeuw

**Affiliations:** 1 Department of Complex Trait Genetics, Centre for Neurogenomics and Cognitive Research, VU University, Amsterdam, The Netherlands; 2 Department of Child and Adolescent Psychology and Psychiatry, section Complex Trait Genetics, Amsterdam Neuroscience, VU University Medical Center, Amsterdam, The Netherlands

**Keywords:** Genetics, Psychiatric disorders

## Abstract

Gene-environment interactions (GxE) are often suggested to play an important role in the aetiology of psychiatric phenotypes, yet so far, only a handful of genome-wide environment interaction studies (GWEIS) of psychiatric phenotypes have been conducted. Representing the most comprehensive effort of its kind to date, we used data from the UK Biobank to perform a series of GWEIS for neuroticism across 25 broadly conceptualised environmental risk factors (trauma, social support, drug use, physical health). We investigated interactions on the level of SNPs, genes, and gene-sets, and computed interaction-based polygenic risk scores (PRS) to predict neuroticism in an independent sample subset (*N* = 10,000). We found that the predictive ability of the interaction-based PRSs did not significantly improve beyond that of a traditional PRS based on SNP main effects from GWAS, but detected one variant and two gene-sets showing significant interaction signal after correction for the number of analysed environments. This study illustrates the possibilities and limitations of a comprehensive GWEIS in currently available sample sizes.

## Introduction

Neuroticism is a personality trait that is characterised by emotion dysregulation and negative affect. It has been thought to confer a general susceptibility to mental health problems, resulting in the frequent experience of negative emotions such as worry, sadness, self-consciousness, or anger^1–3^. High neuroticism is associated with increased psychiatric comorbidity, and there is a substantial overlap between neuroticism and a wide range of psychiatric disorders, particularly depression and anxiety^4–6^. The associated societal costs of neuroticism are substantial^7^, leading to increased use of both mental and physical health services due to poorer overall health and quality of life^8^.

Twin studies have estimated the heritability of neuroticism to be around 40%, with the rest typically attributed to non-shared environmental factors^9–13^. In recent years, the genetic aetiology of neuroticism has been studied using large-scale genome-wide association studies (GWAS) which have uncovered more than a hundred genomic loci that point towards genes and pathways involved in brain functioning^14,15^.

In the epidemiological literature, neuroticism and related phenotypes have been linked with a range of different environmental factors, with traumatic events, childhood maltreatment, and social support receiving the greatest attention^16–23^. Despite such studies consistently implicating environments that are shared within families, twin studies tend to assign very little or no proportion of variance to shared environmental factors:^10–13^ a phenomenon called the ‘the shared environment paradox’^24^.

It has been hypothesised that shared environments simply do not matter as much as do non-shared environments^25^, a notion which has been related to the distinction between the ‘objective’ and ‘effective’ environments^26^. That is, while an environment may ‘objectively’ be shared between family members, their ‘effective’ environment, i.e., the environment as they experience it, is nevertheless unique; as is then also the resulting impact of that environment on each individual.

More recently, Uher and Zwicker proposed that the most parsimonious explanation for this shared environment paradox is the presence of gene-environment interactions (GxE). They argue that GxE would lead monozygotic twins to respond more similarly to shared environmental exposures than dizygotic twins and that GxE should therefore result in a substantial proportion of the shared environmental influences being wrongly attributed to genetic factors, causing an inflation of the heritability estimate instead^24^.

From a biological perspective, GxE can be seen as the process by which environmental influences are moderated by genetic factors (or vice versa). GxE has been speculated to play an integral role in the aetiology of psychiatric phenotypes for a long, as it provides an explanation for why some develop psychiatric symptoms after particular risk exposures while others do not^24,27–30^. Though neuroticism has traditionally been viewed as a relatively stable trait, a more dynamic aetiology has been proposed whereby it is continuously influenced by ongoing gene-environment interactions throughout the life span^31^.

To date, however, there have been few truly genome-wide GxE studies (GWEIS) of psychiatric phenotypes, and the majority of molecular GxE research has been limited to candidate genes^29,32–36^. It is only quite recently that the available data and computational resources have begun to allow for the conduction of GWEIS, but as interactions may require larger sample sizes to detect effects of similar magnitude as main effects, sample size requirements may be even greater for GWEIS than for GWAS^37,38^.

To overcome this, some have reduced the multiple testing burden by pre-selecting variants based on main effects from GWAS^39,40^. While these two-stage approaches could potentially yield more significant SNPs, individual SNP effects are unlikely to yield insight into the higher-order biological mechanisms underlying GxE (as is the case for GWAS^41^), and the lack of genome-wide GxE data limits the opportunity for follow-up analyses such as gene-set analysis, which could elucidate the function of GxE effects^42^. In addition, since interacting SNPs may not display strong main effects, this approach could also lead to potential key interactions going undetected^40,43^. Another option may be to model interactions of individual variants with multiple environments simultaneously^44^, though this is also at the cost of environmental specificity which could complicate the interpretation of any functional follow-up analysis.

Alternatively, global GxE effects across the entire genome may be investigated by estimating the proportion of variance explained by GxE effects^45^, or by modelling interactions with polygenic risk scores constructed using SNP main effects from GWAS^46–49^. But while such approaches may indicate the presence of GxE, they cannot determine which SNPs or genes are driving the interactions. For the purposes of gaining relevant biological information from the GxE analyses, we, therefore, considered GWEIS to be the most suitable approach.

Beyond issues with power, GWEIS requires particular consideration regarding the control of error rate inflation, as it is particularly vulnerable to the effects of heteroscedastic residuals^50^. While this can be resolved with the use of heteroscedasticity consistent, or so-called robust, standard errors^51,52^, these are not currently available in software optimised for large-scale genetic analysis like PLINK^53^, and researchers have had to implement this them themselves^34,54^. Interaction effects may also be confounded by covariate-SNP and covariate-environment interaction effects unless these are accounted for^55^, but doing so can dramatically increase the number of variables analysed and add further computational constraints to this already intensive analysis.

To our knowledge, there have only been three GWEIS of psychiatric phenotypes to date, all of which have focused on depressive symptoms and used some composite measure of stressful life events as environment^32–34^. These studies have found few significant interactions, though only one of these studies featured a sample size close to 100,000 individuals (the rest fewer than 10,000). As such, it is evident that there is a substantial gap in the available genome-wide evidence for GxE in mental health phenotypes in general, including neuroticism for which there are currently none.

To address this, we used data from the UK Biobank^56^ to perform a series of GWEIS for neuroticism, with a total of 25 broadly defined environmental variables (*N* = 84,711–313,339; Table 1). While ensuring proper control for inflation and confounding as mentioned above, we first explored SNP-environment interactions between all 25 environmental variables and a total of 8,614,007 SNPs genome-wide.

**Table 1:** Overview of environmental factors.

Category	UKB ID	Full name	Short name	*N*	Type	Levels	Range
Physical health	2188	Long-standing illness, disability, or infirmity	Disability/infirmity	308,892	Ordinal	2	Yes–No
Physical health	23,104	Body mass index (BMI)	BMI	308,303	Continuous	–	12.8–68.4
Physical health	100,048	Pain types experienced for 3+ months*	Chronic pain	313,219	Count	4	0–3
Physical health	4548	Health satisfaction	Health satisf.	123,307	Ordinal	6	Extremely unhappy–…–Extremely happy
Physical health/Trauma	20,528	Diagnosed with a life-threatening illness	Terminal illness	106,089	Ordinal	3	Never–Yes, but not in the last 12 months–Yes, within the last 12 months
Trauma	20,531	Victim of sexual assault	Sexual assault	105,362	Ordinal	3	Never–Yes, but not in the last 12 months–Yes, within the last 12 months
Trauma	20,529	Victim of physically violent crime	Physical assault	106,257	Ordinal	3	Never–Yes, but not in the last 12 months–Yes, within the last 12 months
Trauma	6145	Illness, injury, bereavement, the stress in last 2 years	Multiple stress	312,278	Count	7	Serious illness, injury, or assault to yourself–…–Death of a close relative–…–None of the above
Trauma/Social support	20,488	Physically abused by family as a child	Child. physical abuse	106,240	Ordinal	5	Never true–…–Sometimes true– ...–Very often true
Trauma/Social support	20,487	Felt hated by a family member as a child	Felt hated	106,183	Ordinal	5	Never true–…–Sometimes true– ...–Very often true
Trauma/Social support	20,489	Felt loved as a child	Felt loved	106,098	Ordinal	5	Never true–…–Sometimes true– ...–Very often true
Social support	20,522	Been in a confiding relationship as an adult	Adult confiding rel.	104,073	Ordinal	5	Never true–…–Sometimes true– ...–Very often true
Social support	1031	Frequency of friend/family visits	Friend/family visits	312,492	Ordinal	7	No friends/family outside household–…–Almost daily
Social support	6160	Leisure/social activities	Social activities	312,940	Count	6	Sports club–…–Pub or social club –…–None of the above
Social support	2110	Able to confide	Able to confide	307,308	Ordinal	6	Never or almost never–…– Almost daily
Social support	4559	Family relationship satisfaction	Family satisf.	122,578	Ordinal	6	Extremely unhappy–…– Extremely happy
Social support	4570	Friendships satisfaction	Friendship satisf.	122,423	Ordinal	6	Extremely unhappy–…– Extremely happy
Sociodemographic	4537	Work/job satisfaction	Work satisf.	84,711	Ordinal	6	Extremely unhappy–…– Extremely happy
Sociodemographic	4581	Financial situation satisfaction	Financial satisf.	123,172	Ordinal	6	Extremely unhappy–…– Extremely happy
Sociodemographic	189	Townsend deprivation index at recruitment	TDI	313,108	Continuous	–	−6.26–11
Sociodemographic/Education	845	Age completed full-time education	Y/o schooling	203,479	Continuous	–	5–35
Cognitive function	20,016	Fluid intelligence score	Intelligence	120,422	Continuous	–	0–13
Sleep	1200	Sleeplessness/insomnia	Insomnia	313,321	Ordinal	3	Never/rarely–Sometimes– Usually
Substance use	1558	Alcohol intake frequency	Alcohol intake	313,339	Ordinal	6	Never–…–Daily or almost daily
Substance use	20,116	Smoking status	Smoking	312,638	Ordinal	3	Never–Previous–Current

628* = item constructed using multiple variables, the UKB ID is the UK Biobank category ID (see “Methods” section).

Given that conceptually meaningful interaction effects may not be evident on the level of individual SNPs, whose effects are likely small in magnitude, we sought to elucidate relevant biological mechanisms that might govern GxE by testing whether single SNP-environment interaction effects were over-represented within particular genes, tissues, or gene-sets. We also evaluated the predictive ability of SNP-interaction effects across the genome by constructing interaction-based polygenic scores (iPRS^GxE^) for each environment and used these to predict neuroticism in an independent subset of the UKB sample (*N* = 10,000).

**Fig. 1:**
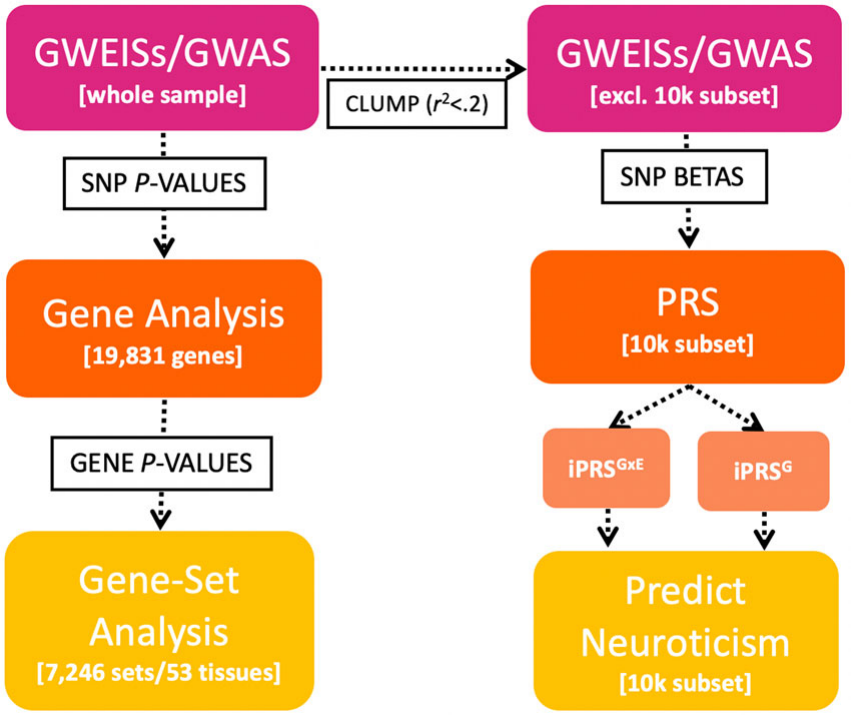
Overview of the analysis workflow. We first obtained SNP-environment interaction effect estimates for the 25 environments (GWEISs), as well as SNP main effects (GWAS). Results from these analyses were used to perform gene and gene-set analyses. The effect of SNPs, genes, and gene-sets that reached standard genome-wide significance (i.e., not corrected for the 25 environments) in the interaction-based analyses were compared with their corresponding main effects. Interaction-based polygenic risk scores (iPRSs) were constructed in an independent subset of the sample to predict neuroticism: this was done by modelling the interactions between each environment and a polygenic risk score constructed based on either the SNP-environment interaction effect from GWEIS (iPRS^GxE^), as well as the SNP main effect from GWAS (iPRS^G^) as a comparison.

In parallel with all interaction-based analyses, we performed a traditional neuroticism GWAS in the same sample to evaluate the concordance between the top interaction effects and corresponding main effects, as well as to allow the predictive power of the iPRSs to be contrasted to that of a traditional main effect PRS constructed from the GWAS results (see Fig. 1 for an overview of the analysis workflow).

The selection of environmental variables was based on the epidemiological literature, and consisted primarily of variables relating to trauma and social support, but also to physical health, socioeconomic status, cognitive function, sleep, and substance use (Table 1; see “Methods” section). While some of these environmental variables are not traditionally seen as ‘environments’ (such as cognitive function, insomnia, BMI), we decided to include these here anyway as they have often been highlighted as risk factors in epidemiological studies in the past^57–59^. Given that many of these environments are themselves heritable, it is thus possible that some interactions we observe could reflect gene-gene interactions (GxG) rather than pure GxE (see Plomin et al.^60^ and Vinkhuyzen et al.^61^ for discussions about the heritability of the environment). Though our rationale for including these is that any potential interactions, be it GxE or GxG, may nonetheless highlight relevant biological mechanisms that contribute to neuroticism. For reasons of convenience, we chose to retain the general term ‘GxE’ throughout the paper, but acknowledge that the term ‘Gene x Trait’ interaction is more suitable.

We note that although a potential correlation between the genetic influences on the environment and the outcome phenotype has been a cause for concern for the estimation of GxE in twin studies^62^, we would not expect this to lead to spurious detection of interaction effects in a GWEIS setting, since the linear regression model allows the SNP and environment main effects to be modelled simultaneously, and can thus account for any correlation that exists between these, as well as with the interaction term.

## Results

### Interacting SNPs implicated by GWEIS

Due to the risk of inflation of the GWEIS test statistics that were mentioned previously^50,54^, we analysed SNP-environment interactions in a linear regression framework in R, computing t-statistics for the interaction coefficients using robust standard errors in the form of the Huber-White sandwich estimator^51,52^ (see “Methods” section for more detail, and Suppl. Info (A): ‘Heteroscedasticity and Spurious Inflation of GWEIS Test Statistics**’** for comparison with traditional, model-based standard errors). In order to account for any potentially confounding covariate interactions^55^, we also included covariate-SNP and covariate-environment interaction effects in the model in addition to covariate main effects (see “Methods” section).

We analysed the single SNP-by-environment interactions between each of the 25 environments (*N* = 84,711–313,339; Table 1) and a total of 8,614,007 SNPs (minor allele frequency >0.01; imputation quality >0.9; see “Methods” section), from which we identified 8 independent SNPs (*r*^2^ < 0.8) for 7 environments that showed interaction effects at the standard genome-wide significance threshold of *p* < 5e−8 (Table 2; Suppl. Figures). Of these, one intergenic SNP on chromosome 6 remained significant after applying further Bonferroni correction for the number of environments analysed (*p* < 5e−8/25 = 2e−9; rs115385310, ‘felt hated as a child’); an SNP which was also suggestively significant for ‘childhood physical abuse’ (*p* = 6.93e−7).

**Table 2:** SNPs-environment interactions detected in the GWEIS.

Environment	SNP	Chr	BP	Gene	P_GWEIS_	P_GWAS_	MAF
Felt hated	rs115385310	6	6,721,120	—	6.09e−10*	0.454	0.02
Able to confide	rs874616	15	87,901,482	—	8.57e−9	0.310	0.66
Work satisf.	rs4461224	2	23,485,507	—	1.44e−8	0.223	0.10
TDI	rs11700517	21	33,273,542	HUNK	1.76e−8	0.195	0.16
Adult confiding rel.	rs12649942	4	38,261,175	—	2.86e–8	0.996	0.58
Social activities	rs111497581	4	91,033,882	CCSER1**	3.98e−8	0.756	0.02
Terminal illness	rs5928040	X	32,623,390	DMD	4.97e−8	0.705	0.19
Terminal illness	rs185839186	5	7,409,995	ADCY2	4.98e−8	0.842	0.02

SNPs from all 25 GWEIS analyses with a *p*-value below the standard genome-wide significance threshold (*p* < 5e−8), i.e., not corrected for the number of environments. * = survived Bonferroni correction for the 25 environments (*p* < 5e−8/25 = 2e−9); ** = SNP located within 15 kb upstream of the transcription start site.

These results are in stark contrast to a traditional GWAS on neuroticism performed using the same main effect covariates as the GWEISs (see “Methods” section), for which 103 independent significant SNPs were detected. For the 8 SNPs that showed significant interactions at the standard genome-wide significance threshold (*p* < 5e−8), we did not find any evidence of a significant main effect in the GWAS (*p* > 0.05; Table 2).

### Genes implicated by SNP-environment interactions

To facilitate functional interpretation of GWEIS results, we sought to determine whether SNP-environment interaction effects across the genome tended to congregate within particular genes. Although any direction of effect is inevitably lost when aggregating the effects from multiple SNPs, this analysis nonetheless provides information about whether variants in certain genes could moderate the effect of specific environmental exposures on the phenotype.

We thus performed 25 gene-based tests for 19,831 protein-coding genes in MAGMA using the interaction *p*-values from the GWEISs as input (see “Methods” section). From these analyses, we found a total of 10 genes from 7 environments that reached standard genome-wide significance, correcting only for the number of genes analysed (*p* < 2.52e−6 (0.05/19,831); Table 3; Suppl. Tables 1a–1y); though none survived further correction for the number of environments (*p* < 2.52e−6/25).

**Table 3:** Genes detected in the MAGMA gene analyses.

Environment	Gene	Chr	BP_START_	BP_STOP_	P_GWEIS_	P_GWAS_
Alcohol intake	CLDN4	7	73,213,872	73,247,014	1.16e−7	0.190
	WBSCR27	7	73,248,920	73,256,865	1.44e−6	0.093
Chronic pain	VPS9D1	16	89,773,542	89,787,394	5.59e−7	0.532
	FANCA	16	89,803,957	89,883,065	1.30e−6	0.016
Intelligence	EIF5A	12	7,210,318	7,215,774	8.43e−7	0.116
	POLE	12	133,200,348	133,263,951	1.18e−6	0.080
Family satisf.	CWC27	5	64,064,757	64,314,590	1.22e−6	0.054
Sexual assault	FHIT	3	59,735,036	61,237,133	1.15e−6	1e−4
Social activities	ZSWIM3	20	44,486,256	44,507,761	1.85e−6	0.097
Y/o schooling	TUSC5	17	1,182,957	1,204,281	2.14e−6	0.702

Results from MAGMA gene analysis of 19,831 genes, using the GWEIS interaction *p*-values as input (*p* < 0.05/19,831 = 2.52e−6); No gene survived additional Bonferroni correction for the number of environments analysed (*p* < 2.52e−6/25 = 1.01e−7).

Similar to the SNP-level results, the concordance between suggestive main and interaction effects on the gene-level was low, and only one gene (FHIT, ‘sexual assault’) reached suggestive significance in the main effects gene analysis for neuroticism (*p* = 1e−4; Table 3; Suppl. Table 1z).

### Gene sets enriched by the most interactable genes

In order to determine whether the most strongly associated genes for any environment (including sub-significant ones) tended to be overrepresented within particular pathways, cellular locations, or implicated in particular tissue-specific gene expression patterns, we performed competitive gene-set and gene-property analyses in MAGMA using the results from the 25 GWEIS-based gene analyses as input. These analyses concerned 7426 gene sets (MSigDB) and 53 tissues (GTEx; see “Methods” section).

At a *p*-value threshold of 6.85e−6 (.05/(7246 + 53)), 12 gene sets from 7 environments were significant (Table 4), but no tissues (Suppl. Tables 2a–2y and 3a–3y). Of these 12 gene sets, two survived the additional correction for the number of environments analysed (*p* < 6.85e-6/25): ‘nucleotide transmembrane transporter activity’ (terminal illness), and ‘glucose binding’ (insomnia).

**Table 4:** Gene-sets detected in the MAGMA gene-set analyses.

Environment	Pathway/Tissue	P_GWEIS_	P_GWAS_
Insomnia	Glucose binding	1.44e–8*	0.095
Terminal illness	Nucleotide transmembrane transport	1.61e–8*	0.151
	Nucleotide transmembrane transporter activity	1.09e–6	0.091
	Nucleotide transport	3.40e–6	0.011
Sexual assault	Telomerase pathway	1.60e–6	0.219
	RNA-dependent DNA biosynthetic process	3.68e–6	0.572
Friendship satisf.	Positive regulation of ion transport	1.70e–6	0.763
	Regulation of metal ion transport	1.71e–6	0.360
	Regulation of calcium ion transport	4.41e–6	0.354
Child. physical abuse	PKA mediated phosphorylation of CREB	4.03e–6	0.645
Physical assault	Negative regulation of DNA metabolic process	4.73e–6	0.254
Family satisf.	Advanced glycosylation endproduct receptor signalling	6.13e–6	0.807

Results from MAGMA analysis of 7,246 MSigDB gene sets and 53 gene expression patterns from GTEx (p < 0.05/(7,246 + 53) = 6.85e–6). * = gene sets that survived Bonferroni correction for the number of analysed environments (p < 6.85e–6/25 = 2.74e–7).

Again, none of the interacting gene-sets showed evidence of a significant, or even suggestively significant, the main effect in neuroticism (Table 4; Suppl. Tables 2z and 3z).

### Interaction-based polygenic risk scores (iPRS)

To evaluate the predictive accuracy of our SNP-environment interactions, we constructed interaction-based polygenic risk scores (iPRS^GxE^)—taking the sum of effect alleles weighted by the interaction beta and the environment—and used these to model neuroticism in an independent subset of the UKB sample (*N* = 10,000; see “Methods” section). An alternative to GWEIS mentioned earlier is to model the interaction between a traditional main effect PRS from GWAS and an environmental variable of interest (henceforth: iPRS^G^). Since the iPRS^G^ is more widely accessible than the iPRS^GxE^ (as it does not require existing GWEISs), we also computed iPRS^G^s for each environment as a comparison.

The variance explained by each iPRS was evaluated by comparing the fit between a full model containing the iPRS and covariates to that of a covariate only model (here, the environment main effect and the main effect PRS were included in addition to the standard covariates used for the GWEIS/GWAS, as well as the interactions between these and the standard covariates; see “Methods” section). This was done using the anova() function in R.

**Fig. 2:**
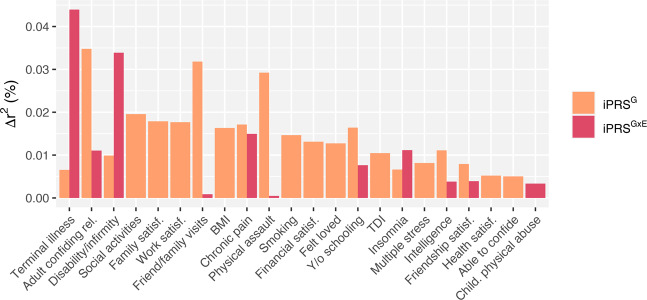
Prediction accuracy of interaction-based polygenic risk scores. Interaction-based polygenic risk scores (iPRS) were computed for an independent subset of 10,000 individuals, using the sum of risk alleles weighted by the SNP betas and the environment. For the iPRS^GxE^s the SNP-environment interaction beta was used as a weight, whereas for the iPRS^G^s, the SNP main effect betas from GWAS was used instead. The *Y*-axis reflects the ∆*r*^2^, i.e., the difference in adjusted *r*^2^ between models containing the iPRSs and all covariates to a covariates-only model. The differences in model fit between the full model and the covariate only model were evaluated using anova in R. Only environments with a ∆*r*^2^ greater than zero are shown; none of the iPRS^G^s or iPRS^GxE^s explained a significant proportion of variance in neuroticism beyond covariates (*p* < 0.05/25/2).

For any of the 25 environments, neither the iPRS^GxE^ nor the iPRS^G^ provided a significant increase in model fit (*p* < 0.05/25/2; see “Methods” section) above that of the covariates only model, with the attributable variance reaching a maximum of .04% for the iPRS^GxE^s and .03% for the iPRS^G^s (Fig. 2). This contrasts with the traditional main effect PRS, which explained 2.06% of the variance in neuroticism beyond standard covariates (*p* = 6.31e−49; see “Methods” section).

We, therefore, conclude that based on the environments and sample population analysed here, there is currently limited evidence that genome-wide GxE effects in the form of iPRSs can improve prediction accuracy in neuroticism beyond what can already be achieved using SNP and environment main effects.

## Discussion

In this study, we have investigated genome-wide gene-environment interactions in neuroticism across a total of 25 different environmental variables previously associated with mental health outcomes. From all SNP, gene, and gene-set based analyses, we detected one SNP (rs115385310 for ‘felt hated as a child’) and two gene-sets (‘glucose binding’ for ‘insomnia’ and ‘nucleotide transmembrane transport’ for ‘terminal illness’) that survived Bonferroni-correction for the number of environments analysed.

Although multiple interactions were found at standard genome-wide significance thresholds (i.e., not correcting for the number of environments), they were substantially fewer than that detected in a traditional GWAS on neuroticism, in which we identified just over 100 independent significant SNPs. This is in line with the notion that the power to detect interactions is lower than that of main effects, and suggests that even larger data sets will be required before we can uncover a more considerable fraction of relevant interactions. The lack of predictive value for interaction-based polygenic risk scores (iPRSs) echoed this further.

A GWEIS analysis will naturally suffer more from an increased multiple testing burden compared to, for example, two-stage GxE approaches which pre-select genetic variants based on their observed main effects. In this study, however, we found that none of the interacting SNPs identified at standard genome-wide significance thresholds (i.e., uncorrected for the number of environments) showed any evidence of even suggestive main effects in the GWAS—the same was largely true the gene and gene-set level results—implying that preselection based on main effects could result in key interactions being overlooked. In addition, as individual SNP interaction effects might themselves not yield notable insight into the biological mechanisms that govern GxE (as is typically the case with single SNP analyses^41^), the genome-wide nature of GWEIS is vital as it allows for follow-up analyses, such as gene-set analysis, which can elucidate the function of GxE effects.

Although the multiple testing burden was further exacerbated by the analysis of multiple environments here, we argue that this approach could enable the identification of common patterns across environments and further strengthen the evidence for any particular gene or pathway (particularly when restricted to environments already thought to be implicated). While a systematic investigation of shared GxE effects was not conducted here due to the lack of power even when not correcting for the environments, we hope that our results may prove useful for researchers conducting similar studies in the future, for example, as a basis for replication or meta-analysis. As increased sample sizes lead to the detection of more reliable SNP-environment interactions, we expect that results from GWEIS and related functional follow-up analyses will become valuable for our understanding of the biological mechanisms that underlie GxE.

In this study, we selected neuroticism as our phenotype of interest due to its significant public health impact^7,8^ and widespread links with several clinical psychiatric disorders^3–6^. Although evidence suggests that neuroticism is more dynamic than traditionally thought^31^, as a personality trait, it may nonetheless be more stable than some clinical phenotypes, such as depression or alcohol use disorder^63–66^, and could also be comparatively less sensitive to GxE. In addition, it should be noted that the UKB sample analysed here consists of a relatively older population, and since the influence of GxE may be more pronounced at an earlier stage of development^67^, the age of this sample might have affected our power to detect certain GxE effects.

Here, we chose to model the relationships between all variables as linear (and thus, treating ordinal environments as continuous), but there is a possibility that some interactions may have a more complex, non-linear form. For instance, for the ordinal environmental variable ‘physical assault’ we now assumed that having been assaulted recently versus in the past results in a similar change in the SNP effect as having been assaulted in the past compared to never. While this may not be a fully accurate representation of the data, we expected that the increased multiple testing resulting from analysing the levels of each ordinal variable separately would nevertheless have had a more severe impact on power.

Finally, we wish to reiterate a key limitation regarding the interpretation of our results in the context of heritable environments. The environmental component in GxE is sometimes seen as an independent force that regulates the penetrance of genetic effects (or vice versa), while in practice, any environmental measure obtained in a cross-sectional design is unlikely to be free from genetic influence^60,61^. Although there have been efforts to distinguish GxG from GxE in the twin modelling literature^62,68^, doing so in this setting is not uncomplicated, and simply conditioning on heritable components could induce collider bias^69^.

In this study, we chose to be particularly lenient with what we considered ‘environment’ in favour of covering as broad a range of relevant variables as possible. Based on these results alone, it is therefore not possible to determine whether any interaction detected here represents one with the environmental components directly (GxE) or with some heritable component thereof (GxG). If well-powered, however, we argue that GWEISs of heritable environments are still useful as they could elucidate important sources of aetiological heterogeneity which can be followed up in greater depth using experimental or more controlled observational designs in the future.

Representing the largest effort of its kind to date, we used a total of 25 environmental variables to investigate gene-environment interactions in neuroticism. Although power is low compared to GWAS, we detected one variant and two gene sets that showed significant interaction after correction for the number of environments analysed. Larger sample sizes are, however, needed to obtain more reliable estimates of relevant SNP-environment interaction effects, which will be required in order to understand the molecular mechanisms that govern gene-environment interactions in neuroticism.

## Methods

### Genotype data and quality control

All genotype and phenotype data were obtained from the UK Biobank^56^ (release 3, March 2018), and this study was conducted under the UK Biobank application 16406. Data collection, primary quality control, and imputation of the genotype data were performed by the UK Biobank itself, the full details of which have been described elsewhere^70^. We applied further quality control in order to ensure the inclusion only of high-quality variants. This entailed filtering SNPs with a minimum info score of .9 (HRC panel imputed), maximum missingness of 5%, and a minor allele frequency of at least 1%, resulting in a total of 8,614,007 SNPs for the analysis.

We used only European, unrelated samples with concordant sex (see Suppl. Info (A): UK Biobank Sample Information and Quality Control). Thirty principal components (computed with FlashPCA^71^) were included as covariates in all analyses to control for population stratification. To ensure that the selection of SNPs remained constant across environments, quality control and filtering were performed on the full subset of individuals with complete neuroticism data (see below), and it is, therefore, possible that exact minor allele frequencies and call rates may vary slightly between the sample subsets for each environment.

### Phenotype

Neuroticism was measured using the Eysenck Personality Questionnaire (Revised Short Form^72^), which contains 12 dichotomous items asking participants to indicate whether they agree with statements such as “Do you worry too long after an embarrassing experience?”, or “Do you ever feel ‘just miserable' for no reason?”. An individual’s level of neuroticism was quantified as the sum of items with which they agreed, ranging from 0 and 12. We included only individuals who had provided complete responses to all items (thus performing no imputation of missing values), resulting in 313,467 samples. To ensure that neuroticism and each environment had been measured simultaneously, we used data collected from the first visit only.

### Environmental factors

We considered broadly as ‘environment’ a wide range of variables available from the UKB Biobank that have been associated with neuroticism and related mental health phenotypes in the literature. This included primarily those relating to trauma exposure^16–20^ and social support^21–23^, but also socioeconomic deprivation^73,74^, education^75^ and cognitive ability^57,76^, substance use^77,78^, sleep^58,79,80^, and physical health (overweight/obesity^59,81^, physical disability^82,83^, chronic pain^84,85^). We gathered all available variables that related to any of these categories, limiting the final selection to a subset of 25. We selected variables as such that there was at least one variable from each category, then giving preference to those with larger total sample sizes and less skew in relation to the remaining variables. Given their central role in the literature, we prioritised a wider selection of items related to trauma and social support but sought to include at least one item related to all other domains. Here, we refer to these variables as ‘environments’ as that is their role in the current analyses while acknowledging that many of the selected environments have a (sometimes considerable) heritable component.

The majority of environments were ordinal, consisting of responses such as ‘never true’ to ‘very often true’, or ‘never or almost never’ to ‘almost daily’ (see Table 1). There were two categorical environments that allowed endorsement of multiple answer options: ‘social activities’ and ‘multiple stress’, which we converted to sum scores representing the number of endorsed options. ‘Chronic pain’ was constructed using a collection of pain items that indicated whether participants had experienced pain in multiple regions for three months or more (category ID: 100048). Scores on this variable reflect the sum of regions in which participants experienced pain for 3+ months, with a maximum score of 3. Indicating no pain or pain for less than 3 months in any number of regions gave a score of 0. Indicating chronic pain in one region gave a score of 1, in two regions a score of 2, and indicating pain all over the body, or pain in three or more regions for 3+ months, gave a score of 3. The reason for this truncation was to allow the inclusion of pain all over the body without making strong assumptions about the severity compared to multiples of separate areas.

To ensure that neuroticism and all environmental measures were measured at the same time point, we analysed data from the first visit only. All environments were analysed as continuous, and as with neuroticism, we performed no imputation of missing responses for any of the environments.

### GWEIS

SNP-environment interactions were analysed in a linear interaction model in R (v3.2.1). As have been shown previously^50,54^, GWEIS test statistics are particularly susceptible to spurious inflation of test statistics due to heteroscedasticity of the residuals. To deal with this, we relied on Huber-White estimated standard errors, also known as a sandwich estimator. Unlike model-based standard errors, which are computed using a single residual variance term for all observations, the sandwich estimator allows a unique residual variances term across observations, approximated using the squared residuals^51,52^.

Our script is an adaptation of a PLINK R plugin originally developed by Almli et al.^54^, which performs a joint test of SNP and SNP-environment interaction effects (https://epstein-software.github.io/robust-joint-interaction). Beyond run-time optimisation, we computed *p*-values for the gene-environment interaction (rather than the joint test of SNP main and interaction effects, as done initially), and included covariate-SNP and covariate-environment interactions in addition to covariate main effects. As has been shown^55^, covariate main effects alone do not effectively control for potentially confounding interactions of the covariate with the SNP or the environment, and unless controlled for, such interactions may be captured in the SNP-environment interaction term. We thus implemented the following linear regression model for every SNP and environment:

$$Y_i = \beta _0 + G_i\beta _G + E_i\beta _E + G_iE_i\beta _{GxE} + C_i^\prime \beta _C + C_i^\prime G_i\beta _{CxG} + C_i^\prime E_i\beta _{CxE} + {\it{\epsilon }}_i$$

where *Y*_*i*_ represents the phenotype measure for any individual *i*, *G*_*i*_ the SNP allele count, and *E*_*i*_ the environmental measure. *C*_*i*_ is a *k* *×* *1* vector of covariates, with *k* equalling the total number of covariates, and *ϵ*_*i*_ the residual, and ′ denotes the transpose. The intercept (*β*_0_) and betas for the SNP (*β*_*G*_), environment (*β*_*E*_), and SNP-environment interaction term (*β*_*GxE*_) are all scalars, while the betas for the covariate-environment (*β*_*CxE*_) and covariate-SNP (*β*_*CxG*_) interactions are *k* *×* *1* vectors. The parameter of interest here is *β*_*GxE*_: the beta for the SNP-environment interaction.

As covariates, we included age, sex, 30 PCs, and all assessment centres with *N* > 10,000. As recommendations or standards regarding the number of PCs that should be included typically concern main effects analyses, we could not exclude the possibility that potentially more complex confounding effects of ancestry might arise when analysing interactions, and therefore chose a more cautious approach of including as many as 30 PCs.

For the analysis, PLINK formatted genotype data was read into R (v3.2.1) using the read.plink() function from the snpStats package (see the Suppl. Info (B)–Analysis script for the full R script). As per the snpStats default settings, autosomal SNPs were coded as 0, 1, and 2, representing the homozygous minor, heterozygous, or homozygous major genotypes, respectively. On the X chromosome, male genotypes were coded as 0 and 2, representing single copies of the minor or major alleles.

### GWAS

We conducted two GWASs of neuroticism in PLINK v.2.0^53^ using the same set of covariates as in the GWEIS: one using the full neuroticism sample (*N* = 313,467), done with the purpose of determining whether interacting SNPs, genes, or gene-sets displayed any main effects on neuroticism, and one that excluded a test set of 10,000 individuals done for the purpose of constructing a main effect polygenic risk score.

### Gene analyses

To investigate whether SNP-environment interaction signals tended to congregate within genic regions, we performed genome-wide gene analyses with MAGMA (v1.07b)^42^ using the *p*-values from the GWEIS as input. Gene locations for 20,260 protein-coding genes were obtained from Ensembl (GRCh37, p13, v96), of which 19,831 contained at least one SNP in our data. To allow the inclusion of nearby, potentially regulatory SNPs, we used windows of 2 kb upstream and 1 kb downstream of the transcription start and stop sites, respectively. For computational efficiency, a random subset of 10,000 individuals from the UKB data set was used as a reference for the estimation of LD.

As an aggregation method for the SNP effects, we employed the ‘multi model’ which is a hybrid between the commonly used ‘mean model’, which simply averages the SNP effects across the gene, and the ’top model’, which uses the lowest SNP *p*-value corrected for gene size. In essence, the ‘multi model’ applies both the ‘mean’ and ‘top’ models and selects the one with the best fit.

### Gene-set and gene property analyses

Competitive gene-set and gene-property analyses were performed for all GWEISs and the GWAS using MAGMA (v1.07b)^42^. A total of 7246 gene set definitions were obtained from MsigDB (v6.2), including gene ontology (GO) terms, cellular locations, and biological pathways from multiple sources (e.g., KEGG, Reactome, BioCarta). These were analysed in a competitive framework (as is the default in MAGMA), testing whether the average association with genes within a gene set is greater than that of genes outside the gene set, while correcting for LD.

To test for tissue specificity of associated genes, we used the recently implemented conditional gene property analysis in MAGMA. In this framework, any given tissue can be conceptualised as a gene-set, where gene mRNA expression levels represent the continuous gene-set membership for any given gene, with its mean gene expression level across tissues included as a covariate. For this analysis, we used the mean log-transformed gene mRNA expression profiles in 53 different tissues obtained from GTEx (v7).

### Polygenic risk scores (PRS)

In order to evaluate the predictive ability of our GWEIS results, we constructed interaction-based polygenic risk scores (iPRS^GxE^) using the SNP-environment interaction effects from each of the 25 GWEISs. As a comparison, we created alternative iPRSs representing the interactions between a standard main effect PRS and each environment (iPRS^G^). We evaluated the predictive accuracy of all iPRSs (i.e., the iPRS^GxE^s and iPRS^G^s) against that of a traditional main effect PRS.

To obtain GWEIS and GWAS effect sizes for these PRS analyses, we excluded a hold-out sample of 10,000 individuals (to be used for prediction) and re-analysed all main and interaction effects as described previously. For each analysis, we extracted the independent significant SNPs using clumping in PLINK^53^ (*r*^2^ < 0.2; 250kb), and used these SNPs to construct PRSs for every individual in the hold-out sample.

The different PRS scores were defined as follows. The standard main effect PRS was computed as ${\mathrm{PRS}}_i = \mathop {\sum}\nolimits_j^k {G_{ij}\beta _j^G}$ for each individual *i*, with *G*_*ij*_ their genotype value for SNP *j* (*k* being the number of SNPs used), and $\beta _j^G$ the GWAS effect size. For environment *E*, the iPRS^G^ score was computed as $iPRS_i^G = PRS_i \times E_i$, and the iPRS^GxE^ as $i{\mathrm{PRS}}_i^{GxE} = \mathop {\sum}\nolimits_j^k {G_{ij}E_i\beta _j^{GxE}}$, with $\beta _j^{GxE}$ the GWEIS interaction effect size of SNP *j*.

A possible alternative to how we computed the iPRS^GxE^ here may be to include the SNP main effect from the interaction analyses in the iPRS^GxE^ itself, i.e., $i{\mathrm{PRS}}_i^{G + GxE} = \mathop {\sum}\nolimits_j^k {G_{ij}E_i\beta _j^{GxE} + G_{ij}\beta _j^G}$. As we are interested in determining the extent to which GxE predicts neuroticism beyond any gene and environment main effects, however, we constructed our $i{\mathrm{PRS}}_i^{GxE}$ using only the interaction terms, and instead included *PRS*_*i*_ as a covariate to account for the genetic main effect.

The PRS scores were constructed using SNPs significant at different *p*-value thresholds (.001, .05, .1, .2, …, .8, .9, 1). For each PRS score, we then fit a linear regression in the hold-out sample with neuroticism as an outcome, with the PRS score and a set of covariates as predictors. An estimate of the predictive ability of the PRS score was then computed as the difference between the adjusted *r*^2^ for this model and the corresponding covariate-only model. Here, we chose to use the adjusted *r*^2^, rather than the full *r*^2^, as this provides an unbiased estimation of the population explained variance in models with multiple predictors. For the main effect PRS, as well as for both the iPRS^GxE^ and iPRS^G^ for each environment, we selected the PRS based on the *p*-value threshold for which the predictive ability was greatest.

As covariates, we used the same base covariates as in the GWEIS/GWAS analyses (age, sex, array, and all assessment centres with N > 50). For the traditional PRS, the covariates only model was $Y_i = \beta _0 + C_i^\prime \beta _C + {\it{\epsilon }}_i$, with *Y*_*i*_ the neuroticism score for any one individual *i* in the holdout sample, *C*_*i*_ the *1* *×* *k* vector of base covariates (with ‘ denoting the transpose, and *k* the number of covariates), *β*_0_ the intercept, *β*_C_ the covariate effect sizes, and *ϵ*_*i*_ the residual. The full model including the PRS is then $Y_i = \beta _0 + C_i^\prime \beta _C + {\mathrm{PRS}}_i\beta _{{\mathrm{PRS}}} + {\it{\epsilon }}_i$, with *PRS*_*i*_ representing the main effect PRS for that individual, and *β*_*PRS*_ the beta coefficient for the PRS on neuroticism in the hold-out sample.

For the iPRS^GxE^ and iPRS^G^ scores, however, we also included the relevant environment and the main effect PRS as covariates, as well interaction between these and the base covariates (similar to the GWEIS setup). Thus, the covariate only model used for any iPRS with environment *E* is:

$$Y_i = \beta _0 + C_i^\prime \beta _C + {\mathrm{PRS}}_i\beta _{{\mathrm{PRS}}} + E_i\beta _E + C_i^\prime {\mathrm{PRS}}_i\beta _{PxC} + C_i^\prime E_i\beta _{ExC} + {\it{\epsilon }}_i$$

with PRS_*i*_ and *E*_*i*_ representing the traditional main effect PRS and the environment, respectively (with *β*_PRS_ and *β*_*E*_ their effect on the neuroticism), and $C_i^\prime {\mathrm{PRS}}_i$ the interaction between the main effect PRS and the covariates (with related effect size *β*_*PxC*_), and $C_i^\prime E_i$ the covariate-environment interaction (with effect size *β*_*ExC*_). The full model for any iPRS would then also contain the term for the iPRS and its effect on neuroticism in addition to all variables in the null model, i.e.:

$$Y_i = \beta _0 + C_i^\prime \beta _C + {\mathrm{PRS}}_i\beta _{{\mathrm{PRS}}} + E_i\beta _E + C_i^\prime {\mathrm{PRS}}_i\beta _{PxC} + C_i^\prime E_i\beta _{ExC} + i{\mathrm{PRS}}_i\beta _{i{\mathrm{PRS}}} + {\it{\epsilon }}_i.$$

The reason why we include the main effect PRS derived from the GWAS as a representation of the SNP main effects, rather than simply a PRS constructed from the SNP main effect from the GWEIS, is because the GWEIS PRSs will have been pruned based on the interaction effects, and will thus underestimate the total amount of variance contributed by SNP main effects across the genome. Since we are specifically interested in how much the iPRSs contribute above and beyond what can be obtained using a simple main effect PRS from GWAS, the SNP main effects as obtained in the GWEIS would not have been appropriate.

## Supplementary information

Suppl. Figures

Suppl. Info (A)

Suppl. Info (B) - Analysis Script

Suppl. Tables 1a-1z (genes)

Suppl. Tables 2a-2z (gene sets)

Suppl. Tables 3a-3z (tissues)

## Data Availability

Summary statistics from all the 25 GWEISs, as well as the neuroticism GWAS can be downloaded from the website of the Department of Complex Trait Genetics, CNCR (http://ctg.cncr.nl). Summary statistics from the gene, gene-set, and gene-property analyses are available in Suppl. Tables 1–3.

## Code availability

The full R script used to perform the GWEIS analyses can be accessed in Suppl. Info (B)–Analysis script. Analysis scripts used for the PRS, gene-, gene-set, or gene-property analyses are available from the authors upon request.
