# Correction to: Genome-wide gene-environment interactions in neuroticism: an exploratory study across 25 environments

**DOI:** 10.1038/s41398-021-01334-6

**Published:** 2021-04-08

**Authors:** Josefin Werme, Sophie van der Sluis, Danielle Posthuma, Christiaan A. de Leeuw

**Affiliations:** 1 Department of Complex Trait Genetics, Centre for Neurogenomics and Cognitive Research, VU University, Amsterdam, The Netherlands; 2 Department of Child and Adolescent Psychology and Psychiatry, section Complex Trait Genetics, Amsterdam Neuroscience, VU University Medical Center, Amsterdam, The Netherlands

**Keywords:** Genetics, Psychiatric disorders

Correction to: *Translational Psychiatry*

10.1038/s41398-021-01288-9 published online 22 March 2021

The original version of this article unfortunately contained a mistake. There is a typo in Table 2. The typo concerns the chromosome number for SNP rs11700517 within the HUNK gene (phenotype TDI). It stated chromosome 1, but it should be 21. The authors apologize for the error. The original article has been corrected.

