# Correction: Genome-wide gene-environment interactions in neuroticism: an exploratory study across 25 environments

**DOI:** 10.1038/s41398-022-01974-2

**Published:** 2022-05-13

**Authors:** Josefin Werme, Sophie van der Sluis, Danielle Posthuma, Christiaan A. de Leeuw

**Affiliations:** 1 Department of Complex Trait Genetics, Centre for Neurogenomics and Cognitive Research, VU University, Amsterdam, The Netherlands; 2 Department of Child and Adolescent Psychology and Psychiatry, section Complex Trait Genetics, Amsterdam Neuroscience, VU University Medical Center, Amsterdam, The Netherlands

**Keywords:** Genetics, Psychiatric disorders

Correction to: *Translational Psychiatry* 10.1038/s41398-021-01288-9, published online 22 March 2021

The original version of this article contained a mistake in the Acknowledgements. The following sentence should be changed from “This work was funded by COSYN (Comorbidity and Synapse Biology in Clinically Overlapping Psychiatric Disorders: Horizon 2020 Program of the European Union under RIA grant agreement 667301 to D.P.) and the Netherlands Organization for Scientific Research (NWO: VICI 435-14-005).” to “This work was funded by COSYN (Comorbidity and Synapse Biology in Clinically Overlapping Psychiatric Disorders: Horizon 2020 Program of the European Union under RIA grant agreement 667301 to D.P.), a European Research Council advanced grant (Grant no, ERC-2018-AdG GWAS2FUNC 834057 [to DP]), and the Netherlands Organization for Scientific Research (NWO: VICI 435-14-005).” The authors apologize for the mistake. The original article has been corrected.

