# Supplementary material for: Genome-wide gene-environment interactions in neuroticism: an exploratory study across 25 environments
Source: Transl Psychiatry. 2021 Mar 22;11:180. doi: 10.1038/s41398-021-01288-9 (PMC7985503; doi:10.1038/s41398-021-01288-9)
Supplement: Supplementary file 1 — Suppl. Figures [file 41398_2021_1288_MOESM1_ESM.pdf]

Supplementary Figures

Table of Contents

PER ENVIRONMENT 2
Work Satisfaction *3*
Townsend Deprivation Index (TDI) *5*
Social Activities *7*
Felt Hated by Family Member as a Child *9*
Able to Confide *11*
Diagnosed with Life-Threatening Illness *13*
Been in a Confiding Relationship as an Adult *17*
Alcohol Intake Frequency *19*
Victim of Sexual Assault *20*
Chronic Pain *21*
Age Completed Full-Time Education *22*
Family Relationship Satisfaction *23*
Fluid Intelligence Score *24*
Frequency of Friend/Family Visits *25*
Sleeplessness / Insomnia *26*
Long-Standing Illness, Disability, or Infirmity *27*
Health Satisfaction *28*
Friendships Satisfaction *29*
Financial Situation Satisfaction *30*
Illness, Injury, Bereavement, Stress in the Last 2 years *31*
Smoking Status *32*
Physically Abused by Family as a Child *33*
Felt Loved as a Child *34*
Victim of Physically Violent Crime *35*
Body Mass Index (BMI) *36*
MANHATTAN PLOTS (grouped) 37
GWEIS *38*
Gene Analysis *40*

PER ENVIRONMENT

This section contains relevant analysis plots organized by environment. For all environments, there are Manhattan plots for the GWEIS and gene-analysis, and for those with any genome-wide significant SNPs form the GWEIS (i.e. not corrected across environments), there are also annotated locus plots and interaction plots.

GWEIS Manhattan plot
Shows the -log10 *p*-values for the SNP interaction with the environment in question. Gene names have been annotated to suggestively significant (p < 1e-5) intragenic SNPs with the lowest *p*-value.

Locus plot
Shows all SNPs in the genome-wide significant loci with CADD and RegulomeDB score annotations (obtained from FUMA[1]).

Interaction plots
These plots show the average predicted neuroticism at any given combination of alleles and environmental exposure. To illustrate the components that are specific to the SNP-environment interaction of interest, we plotted neuroticism predicted from the full model (as analysed in the GWEIS, i.e. with the SNP-environment interaction term, SNP/environment/covariate main effects, and covariate-SNP/environment interaction effects) as well as using the same model but excluding the SNP-environment interaction term.

Gene analysis Manhattan plot
Shows the -log10 *p*-values for the MAGMA gene analysis (based on the GWEIS SNP-environment interaction results). Suggestively significant genes (p < 1e-4) have been annotated.

1. *Watanabe, K., Taskesen, E., van Bochoven, A. & Posthuma, D. Functional mapping and annotation of genetic associations with FUMA.* Nat. Commun. ***8**, 1826 (2017).*

Work Satisfaction

GWEIS

Manhattan plot

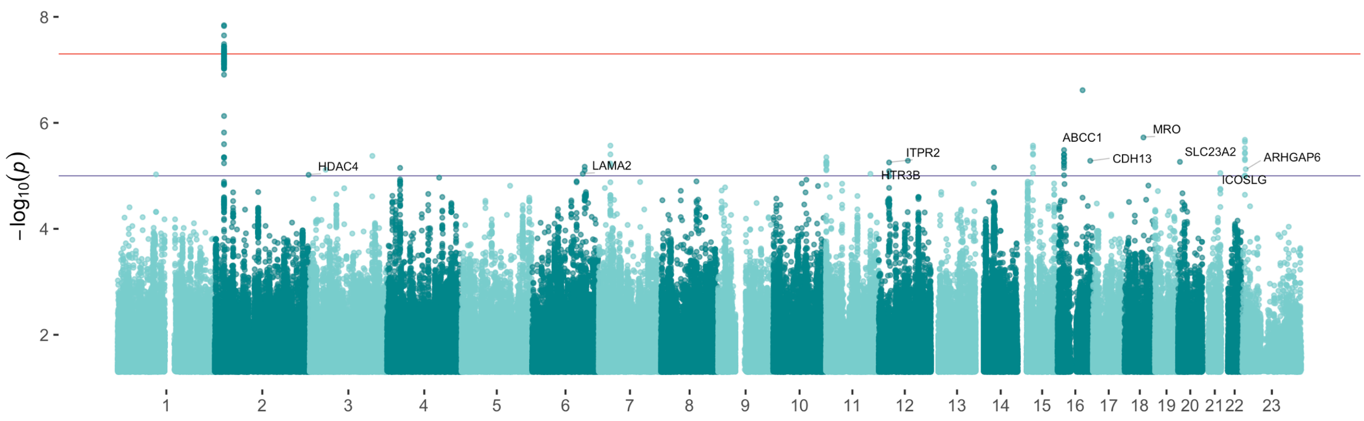

Supplementary Figure 1.1 *Manhattan plot showing the -log10 SNP-environment interaction p-values from the GWEIS. Gene labels have been annotated to suggestively significant (p < 1e-5) intragenic SNPs with the lowest p-value. (Only SNPs with p < .05 were plotted).*

Locus Plot (rs4461224)

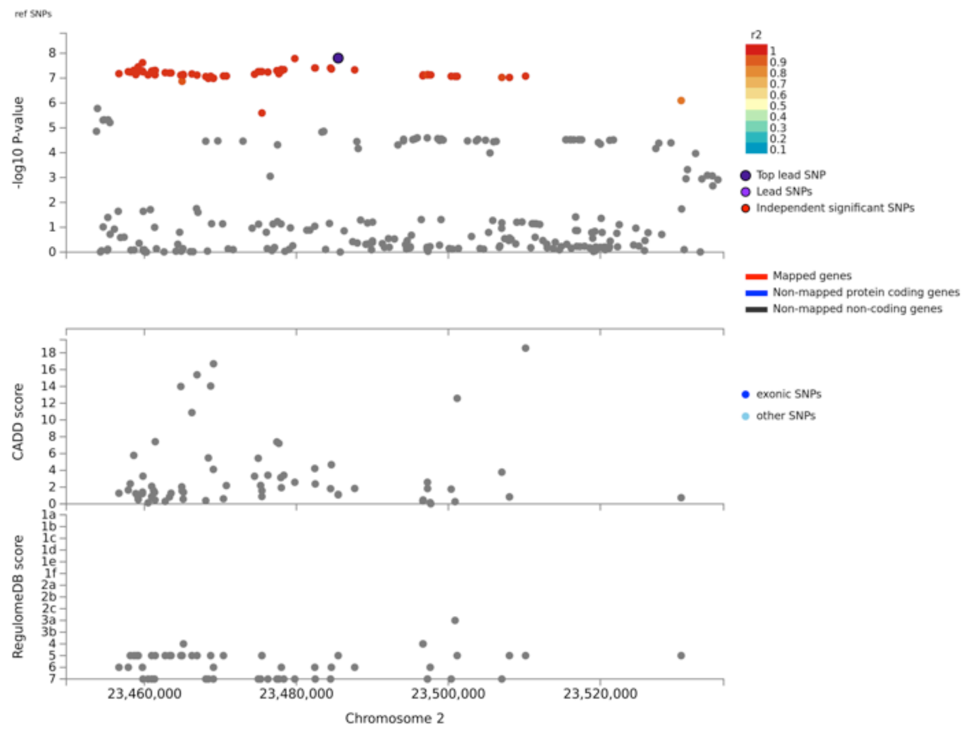

Supplementary Figure 1.2 *Annotated locus plot showing the CADD and RegulomeDB scores for the SNP that reached standard genome-wide significance (p < 5e-8).*

Interaction Plots (rs4461224)

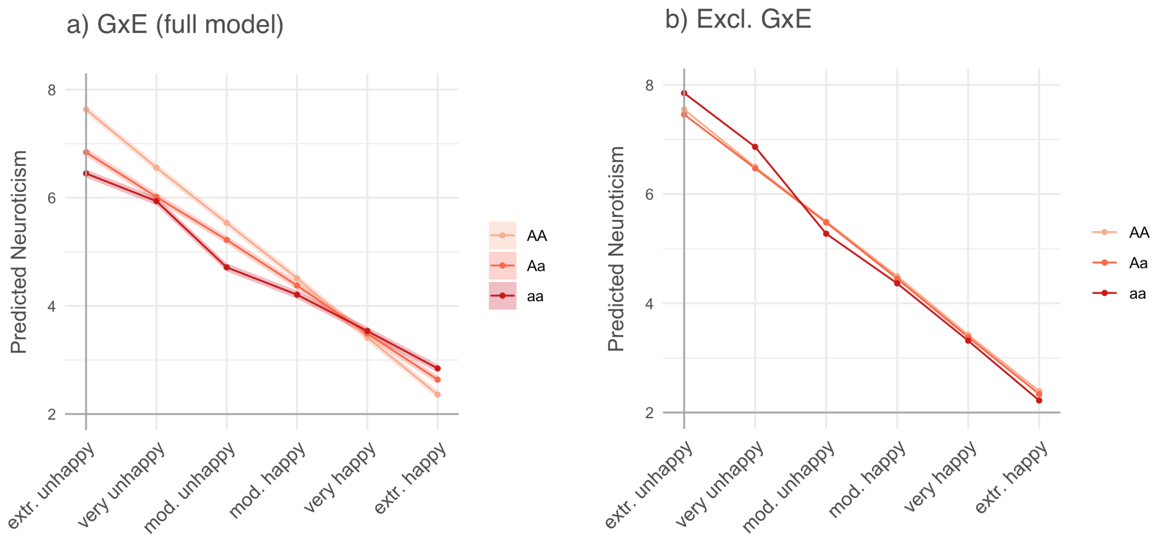

Supplementary Figure 1.3 *Average predicted neuroticism based on a) the full model (i.e., all main and interaction effects), with 95% confidence intervals for the GxE term, or b) everything except the SNP-environment interaction of interest (incl. all main effects and covariate interaction effects). Plot b will show any potential interaction effect captured by the covariates.*

Gene Analysis

Manhattan Plot

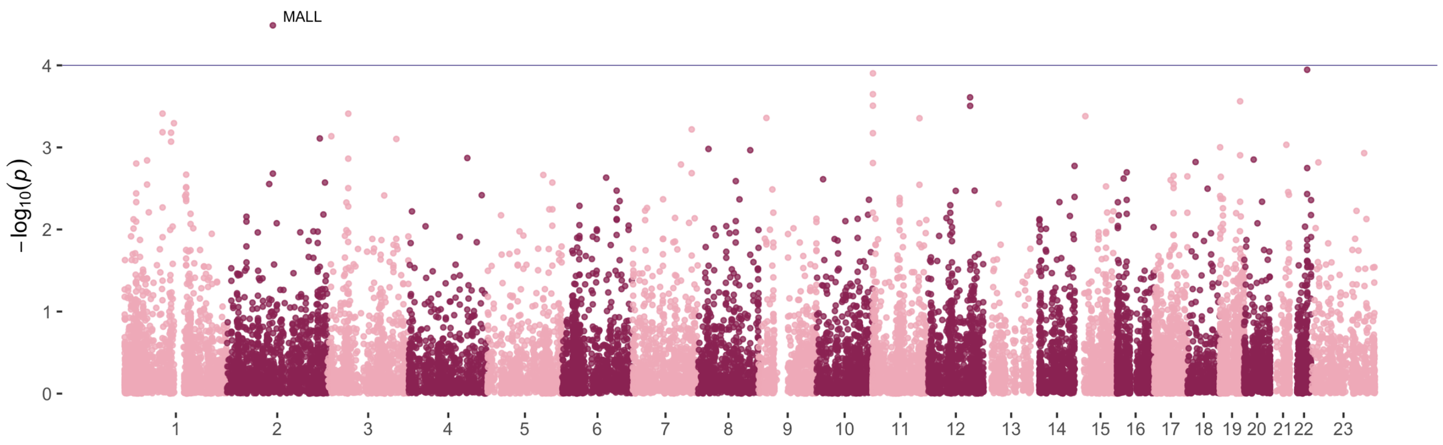

Supplementary Figure 1.4 *Manhattan plot showing the -log10 p-values for the MAGMA gene analysis (based on the GWEIS SNP-environment interaction results). Suggestively significant genes (p < 1e-4) have been annotated.*

Townsend Deprivation Index (TDI)

GWEIS

Manhattan Plot

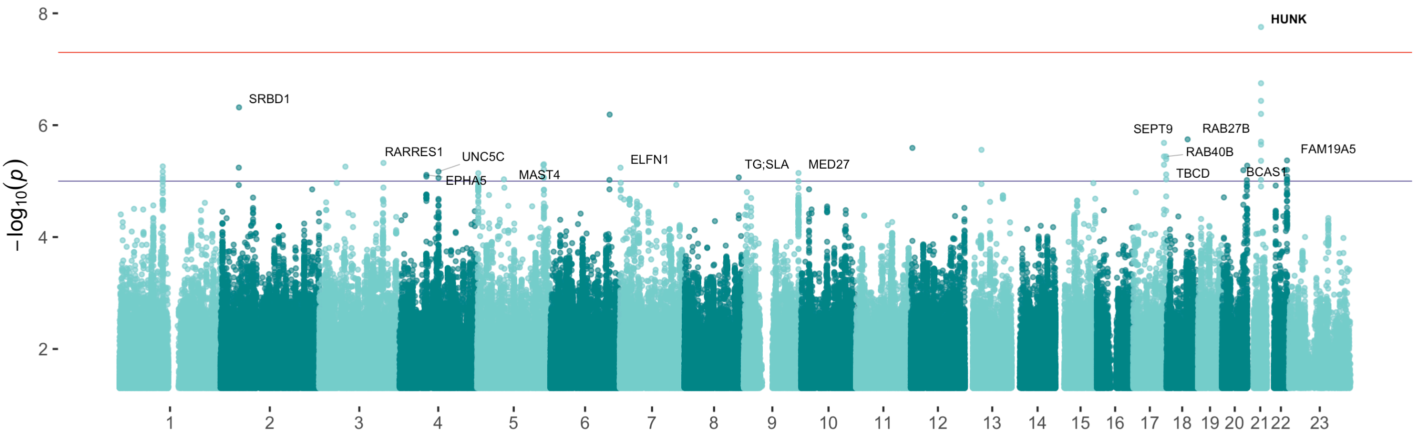

Supplementary Figure 2.1 *Manhattan plot showing the -log10 SNP-environment interaction p-values from the GWEIS. Gene labels have been annotated to suggestively significant (p < 1e-5) intragenic SNPs with the lowest p-value. (Only SNPs with p < .05 were plotted).*

Locus Plot (rs11700517)

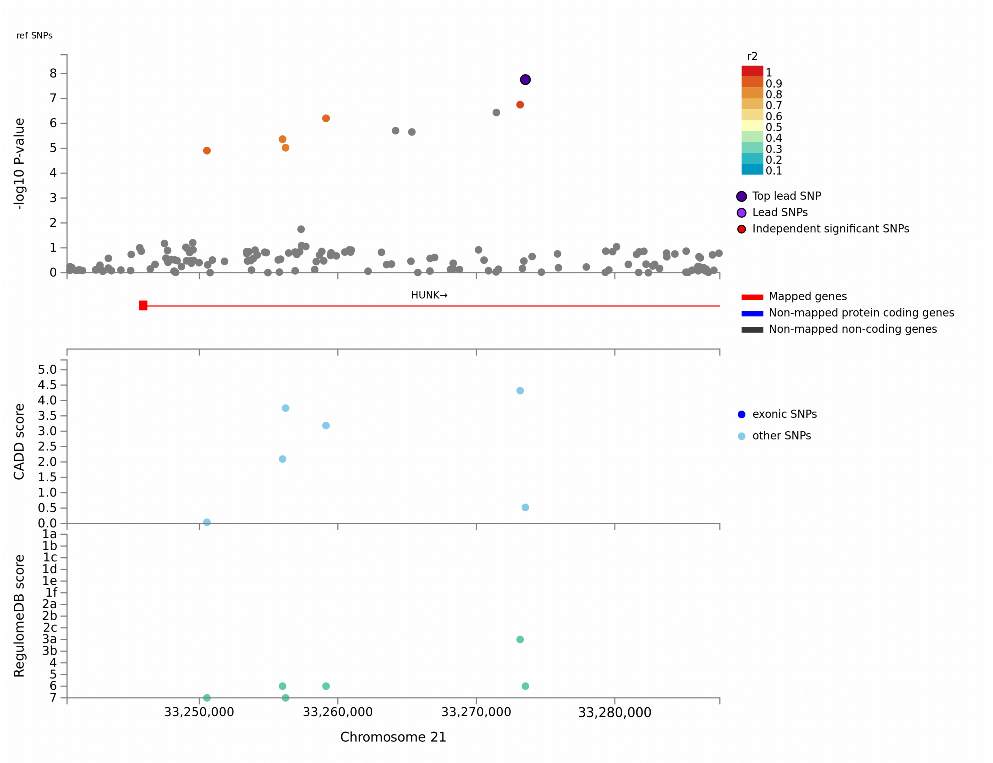

Supplementary Figure 2.2 *Annotated locus plot showing the CADD and RegulomeDB scores for the SNP that reached standard genome-wide significance (p < 5e-8).*

Interaction Plots (rs11700517)

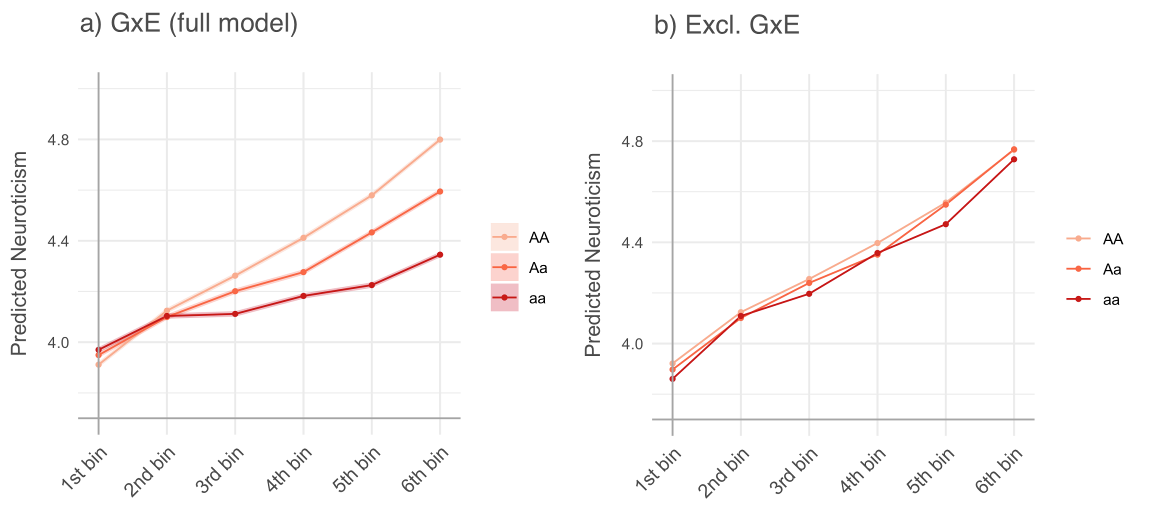

Supplementary Figure 2.3 *Average predicted neuroticism based on a) the full model (i.e., all main and interaction effects), with 95% confidence intervals for the GxE term, or b) everything except the SNP-environment interaction of interest (incl. all main effects and covariate interaction effects). Plot b will show any potential interaction effect captured by the covariates. (For plotting purposes, TDI was split into six bins).*

Gene Analysis

Manhattan plot

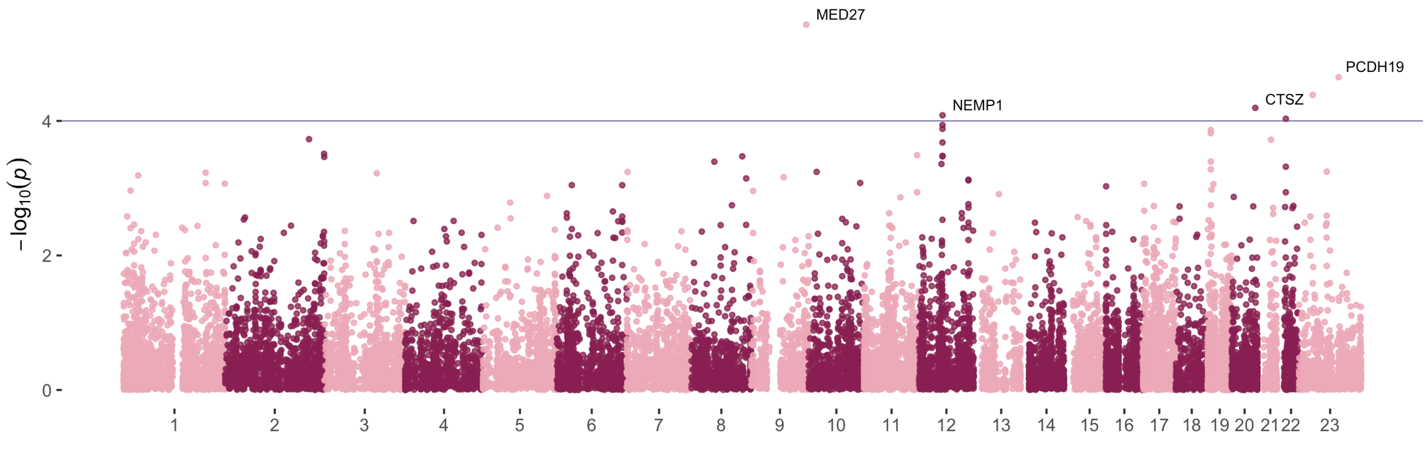

Supplementary Figure 2.4 *Manhattan plot showing the -log10 p-values for the MAGMA gene analysis (based on the GWEIS SNP-environment interaction results). Suggestively significant genes (p < 1e-4) have been annotated.*

Social Activities

GWEIS

Manhattan Plot

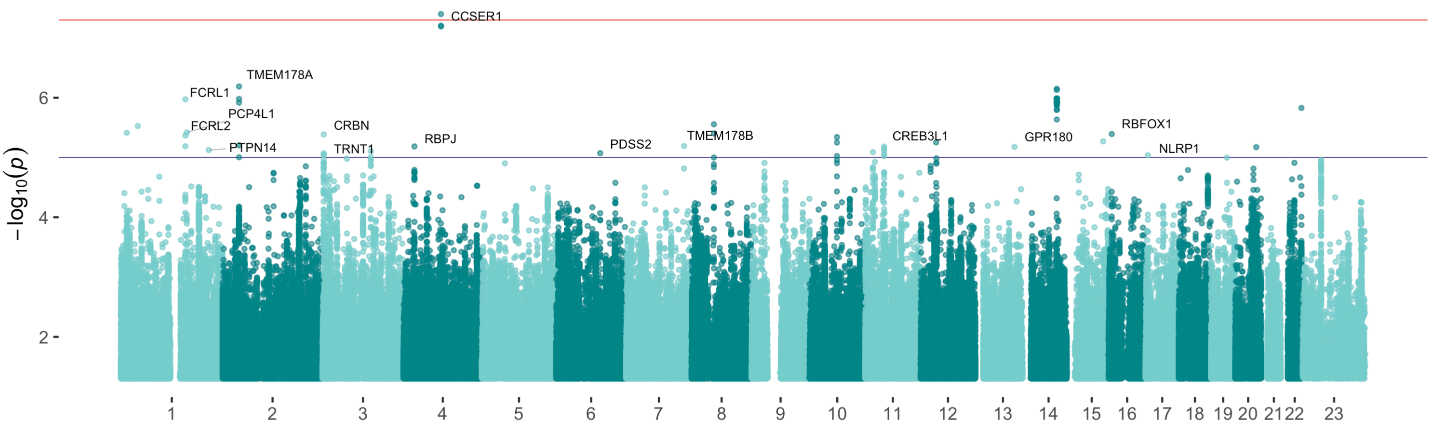

Supplementary Figure 3.1 *Manhattan plot showing the -log10 SNP-environment interaction p-values from the GWEIS. Gene labels have been annotated to suggestively significant (p < 1e-5) intragenic SNPs with the lowest p-value. (Only SNPs with p < .05 were plotted).*

Locus Plot (rs111497581)

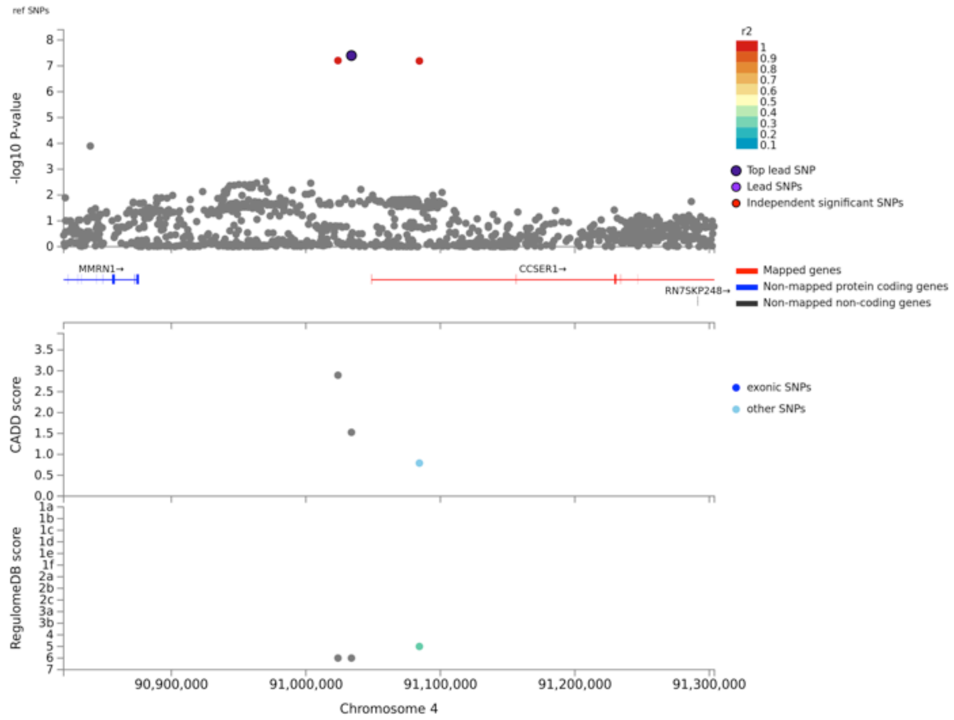

Supplementary Figure 3.2 *Annotated locus plot showing the CADD and RegulomeDB scores for the SNP that reached standard genome-wide significance (p < 5e-8).*

Interaction Plots (rs111497581)

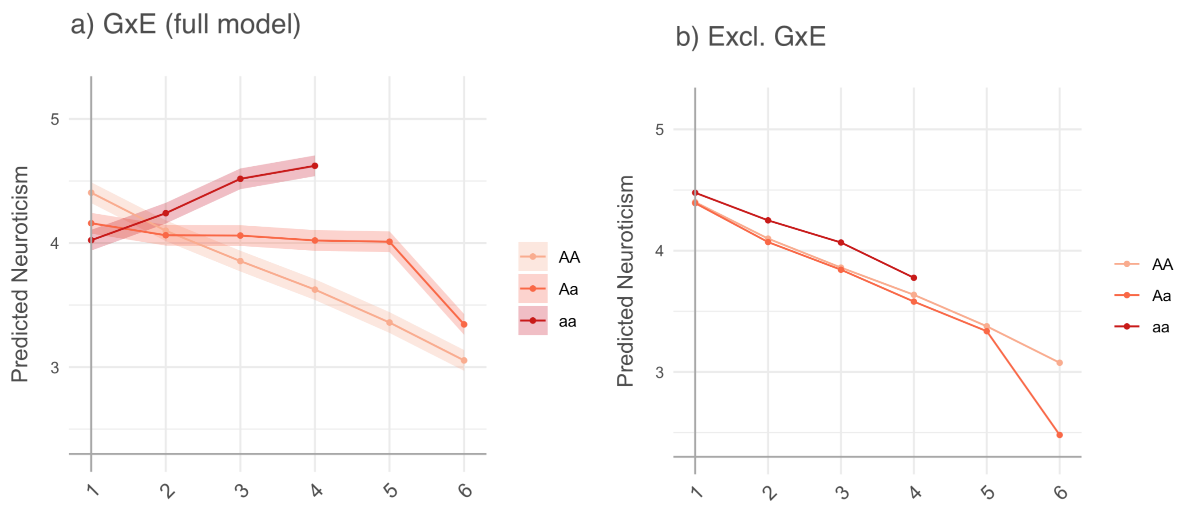

Supplementary Figure 3.3 *Average predicted neuroticism based on a) the full model (i.e., all main and interaction effects), with 95% confidence intervals for the GxE term, or b) everything except the SNP-environment interaction of interest (incl. all main effects and covariate interaction effects). Plot b will show any potential interaction effect captured by the covariates.*

Gene Analysis

Manhattan Plot

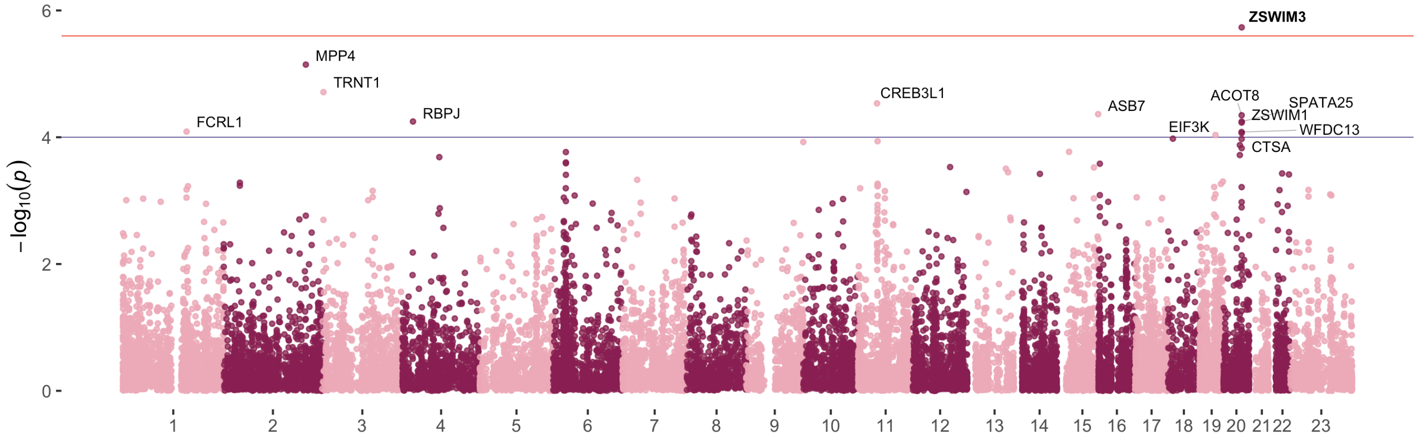

Supplementary Figure 3.4 *Manhattan plot showing the -log10 p-values for the MAGMA gene analysis (based on the GWEIS SNP-environment interaction results). Suggestively significant genes (p < 1e-4) have been annotated.*

Felt Hated by Family Member as a Child

GWEIS

Manhattan Plot

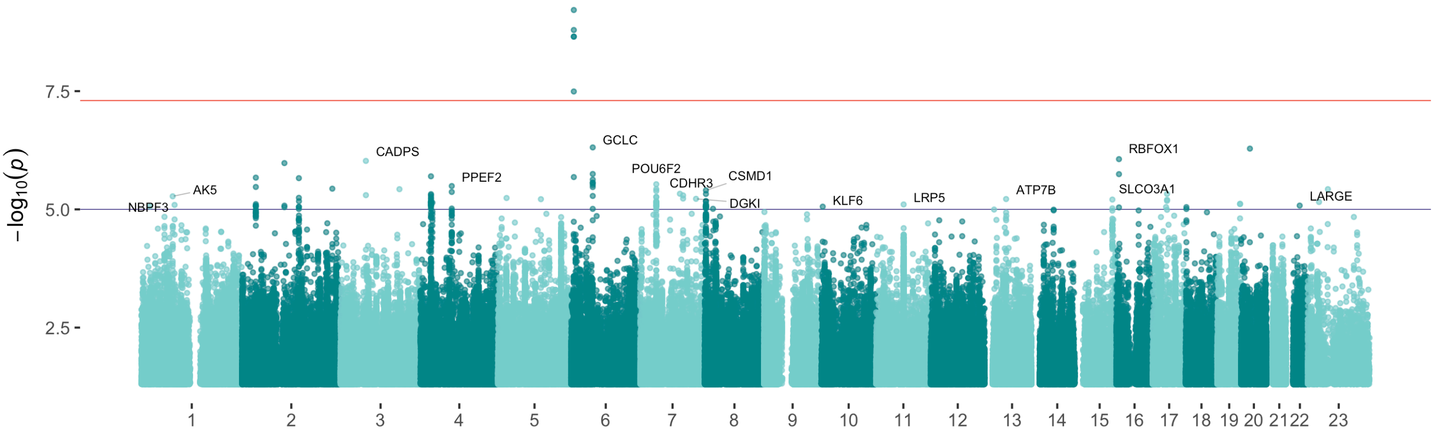

Supplementary Figure 4.1 *Manhattan plot showing the -log10 SNP-environment interaction p-values from the GWEIS. Gene labels have been annotated to suggestively significant (p < 1e-5) intragenic SNPs with the lowest p-value. (Only SNPs with p < .05 were plotted).*

Locus Plot (rs115385310)

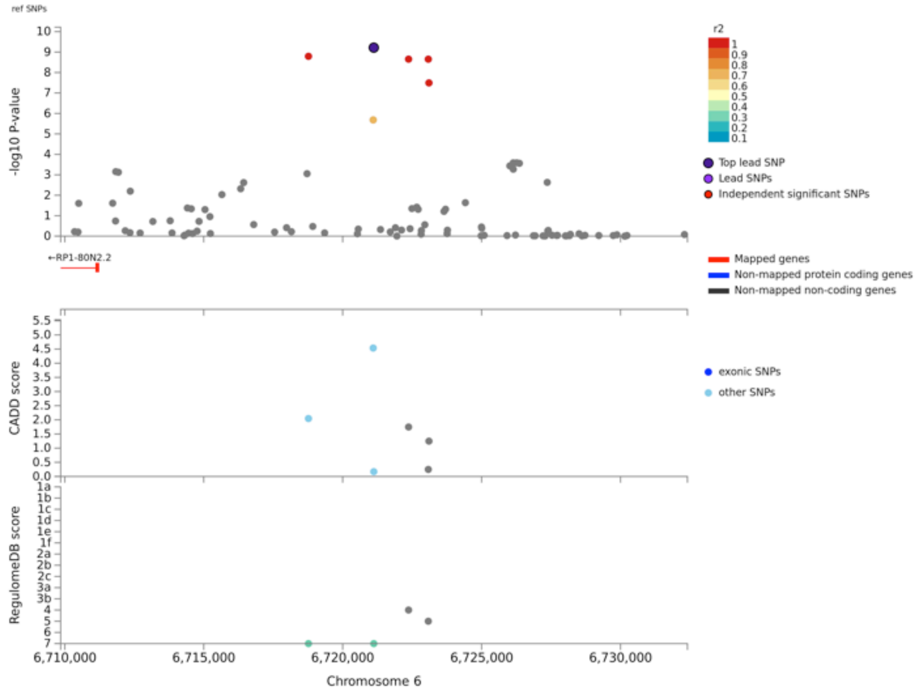

Supplementary Figure 4.2 *Annotated locus plot showing the CADD and RegulomeDB scores for the SNP that reached standard genome-wide significance (p < 5e-8).*

Interaction Plots (rs115385310)

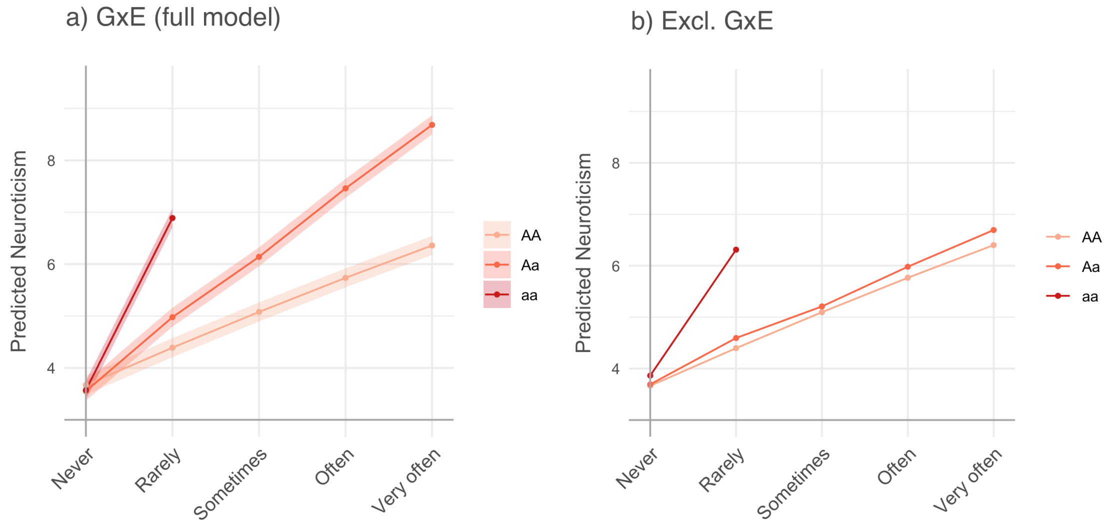

Supplementary Figure 4.3 *Average predicted neuroticism based on a) the full model (i.e., all main and interaction effects), with 95% confidence intervals for the GxE term, or b) everything except the SNP-environment interaction of interest (incl. all main effects and covariate interaction effects). Plot b will show any potential interaction effect captured by the covariates.*

Gene Analysis

Manhattan Plot

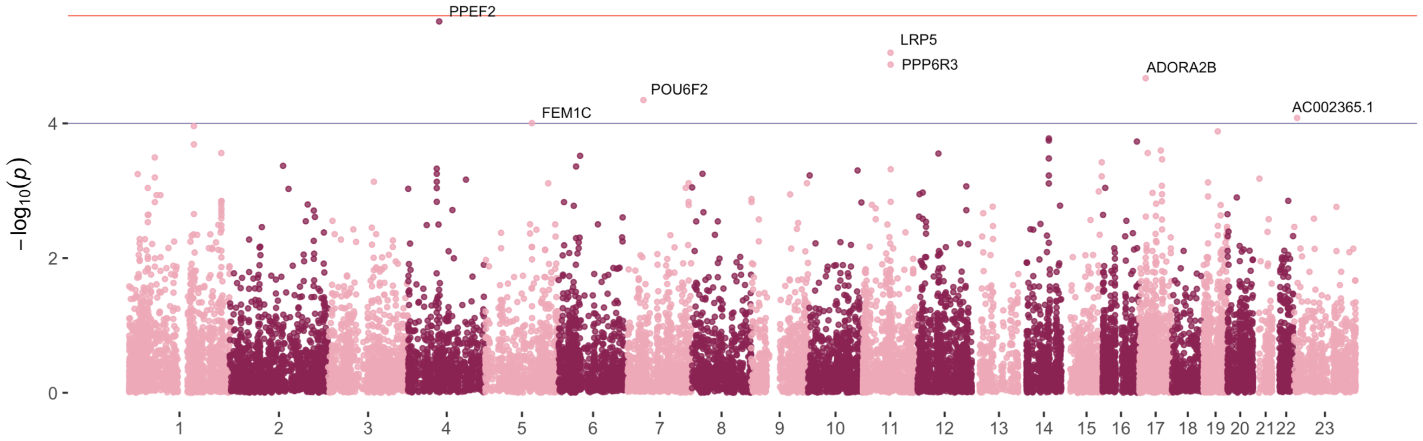

Supplementary Figure 4.4 *Manhattan plot showing the -log10 p-values for the MAGMA gene analysis (based on the GWEIS SNP-environment interaction results). Suggestively significant genes (p < 1e-4) have been annotated.*

Able to Confide

GWEIS

Manhattan Plot

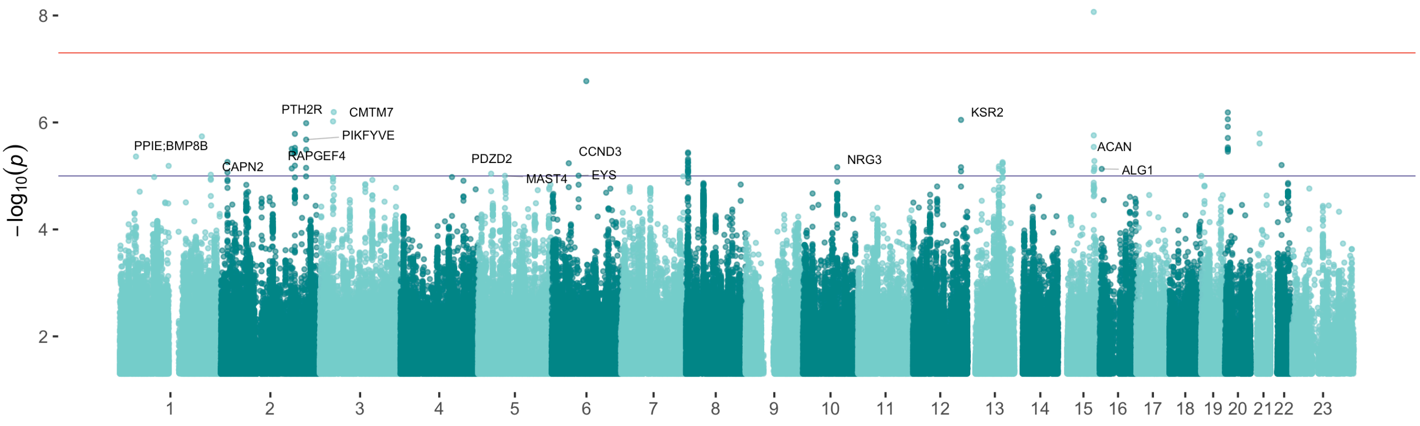

Supplementary Figure 5.1 *Manhattan plot showing the -log10 SNP-environment interaction p-values from the GWEIS. Gene labels have been annotated to suggestively significant (p < 1e-5) intragenic SNPs with the lowest p-value. (Only SNPs with p < .05 were plotted).*

Locus Plot (rs874616)

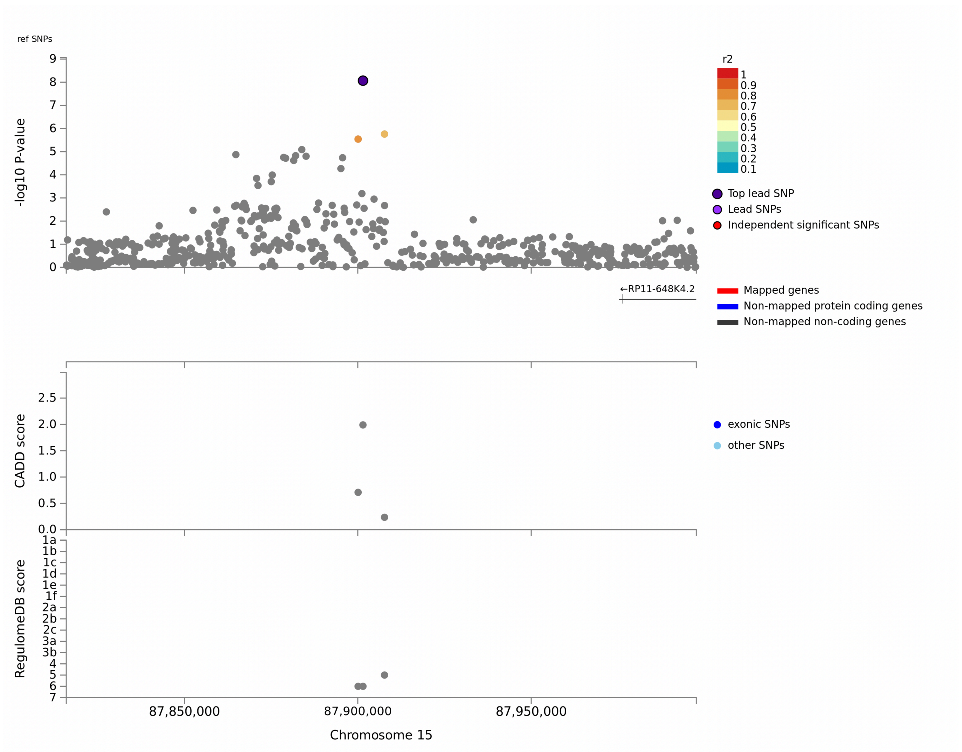

Supplementary Figure 5.2 *Annotated locus plot showing the CADD and RegulomeDB scores for the SNP that reached standard genome-wide significance (p < 5e-8).*

Interaction Plots (rs874616)

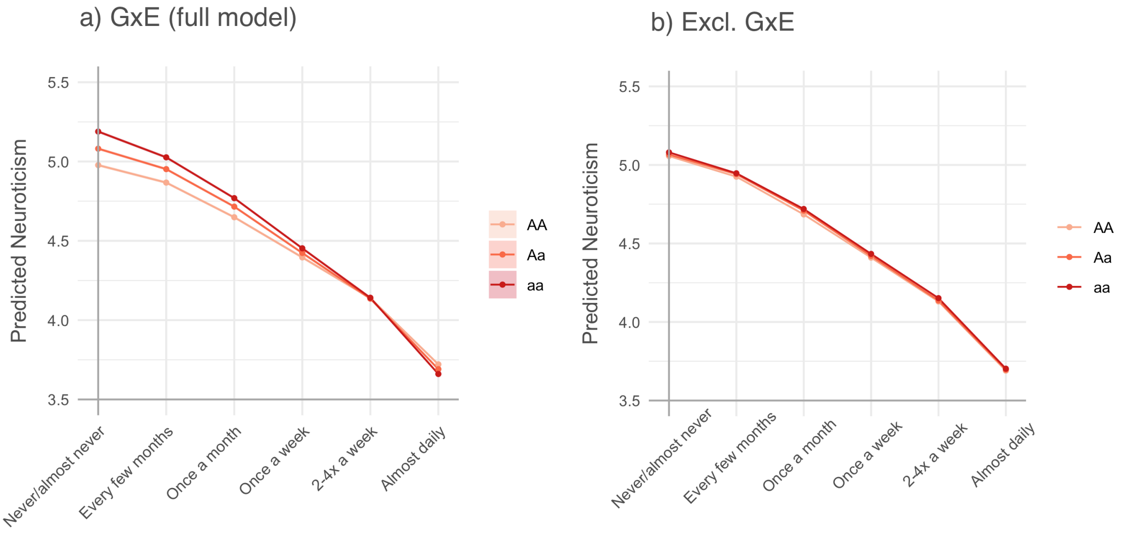

Supplementary Figure 5.3 *Average predicted neuroticism based on a) the full model (i.e., all main and interaction effects), with 95% confidence intervals for the GxE term, or b) everything except the SNP-environment interaction of interest (incl. all main effects and covariate interaction effects). Plot b will show any potential interaction effect captured by the covariates.*

Gene Analysis

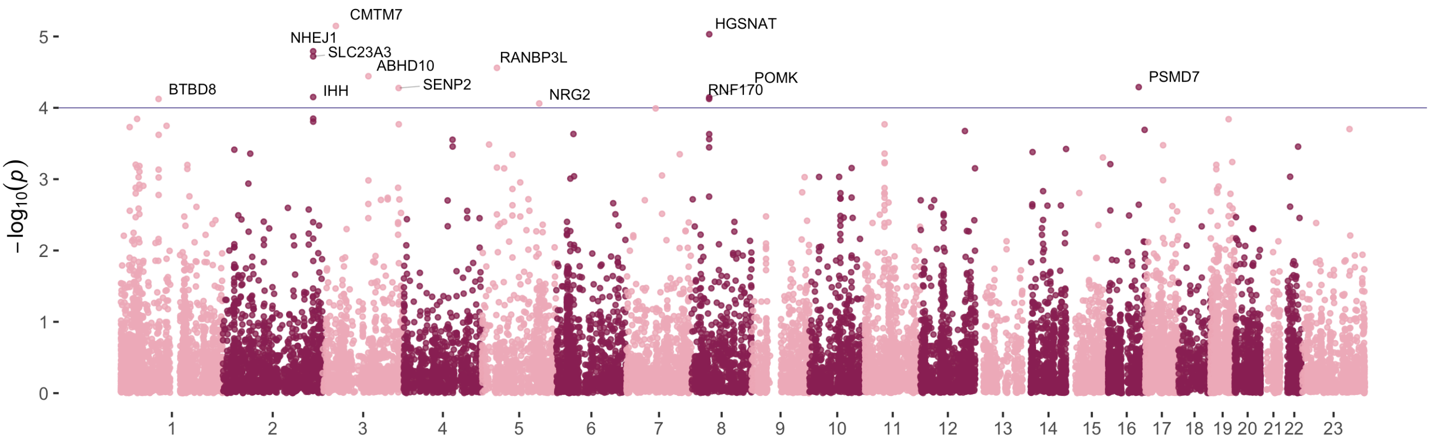

Supplementary Figure 5.4 *Manhattan plot showing the -log10 p-values for the MAGMA gene analysis (based on the GWEIS SNP-environment interaction results). Suggestively significant genes (p < 1e-4) have been annotated.*

Diagnosed with Life-Threatening Illness

GWEIS

Manhattan Plot

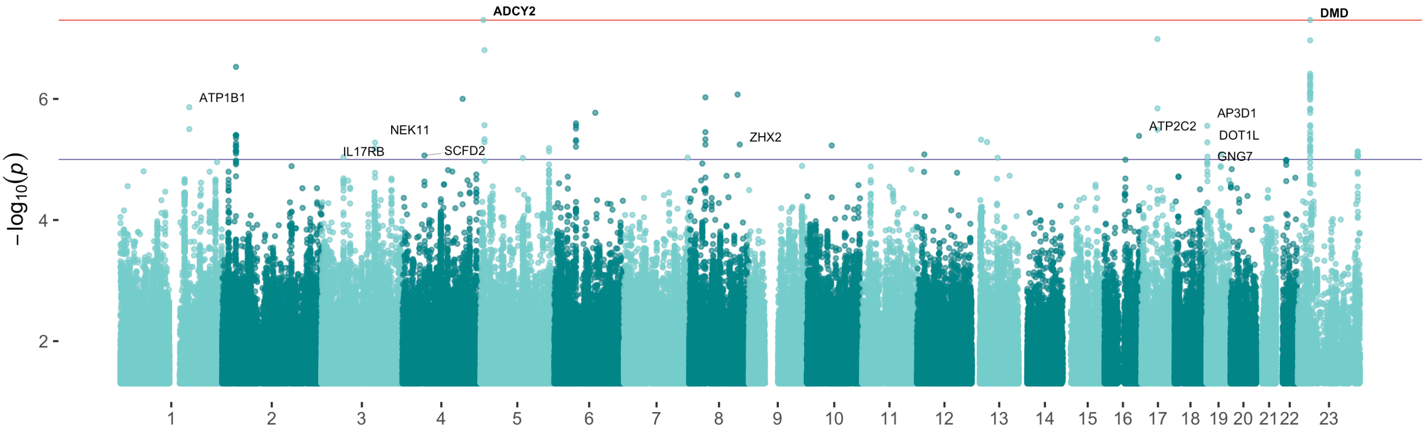

Supplementary Figure 6.1 *Manhattan plot showing the -log10 SNP-environment interaction p-values from the GWEIS. Gene labels have been annotated to suggestively significant (p < 1e-5) intragenic SNPs with the lowest p-value. (Only SNPs with p < .05 were plotted).*

Locus Plots (rs185839186, ADCY2)

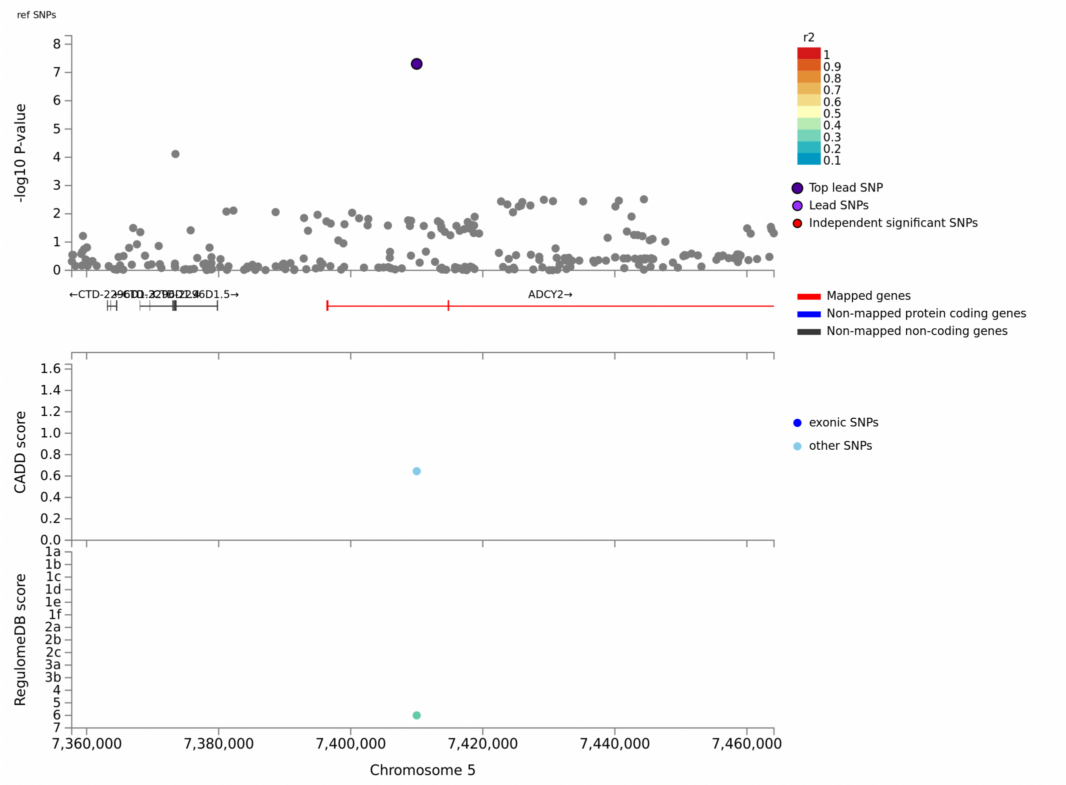

Supplementary Figure 6.2 *Annotated locus plot showing the CADD and RegulomeDB scores for one of the SNPs that reached standard genome-wide significance (p < 5e-8).*

Interaction Plots (rs185839186, ADCY2)

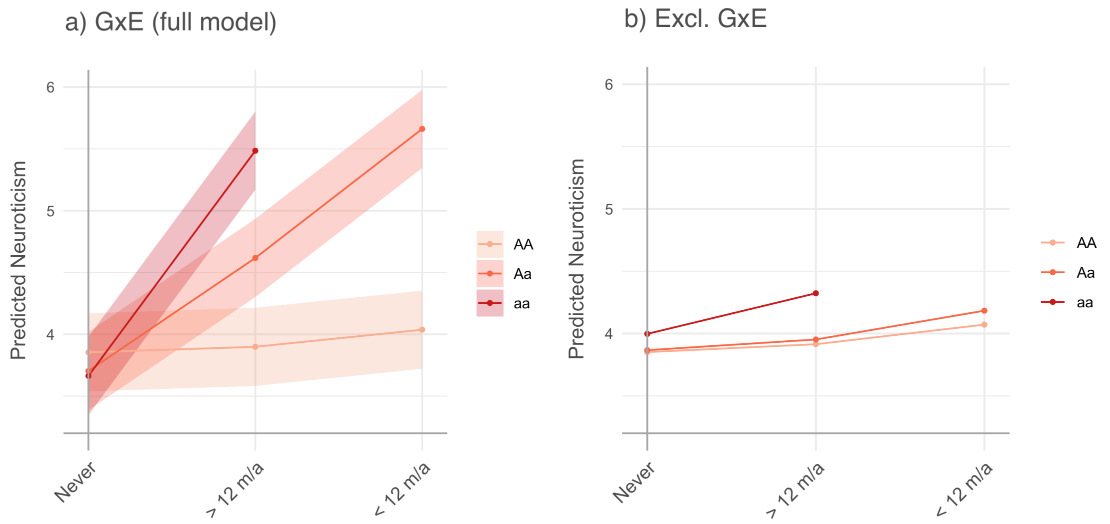

Supplementary Figure 6.3 *Average predicted neuroticism based on a) the full model (i.e., all main and interaction effects), with 95% confidence intervals for the GxE term, or b) everything except the SNP-environment interaction of interest (incl. all main effects and covariate interaction effects). Plot b will show any potential interaction effect captured by the covariates.*

Locus Plot (rs5928040, DMD)

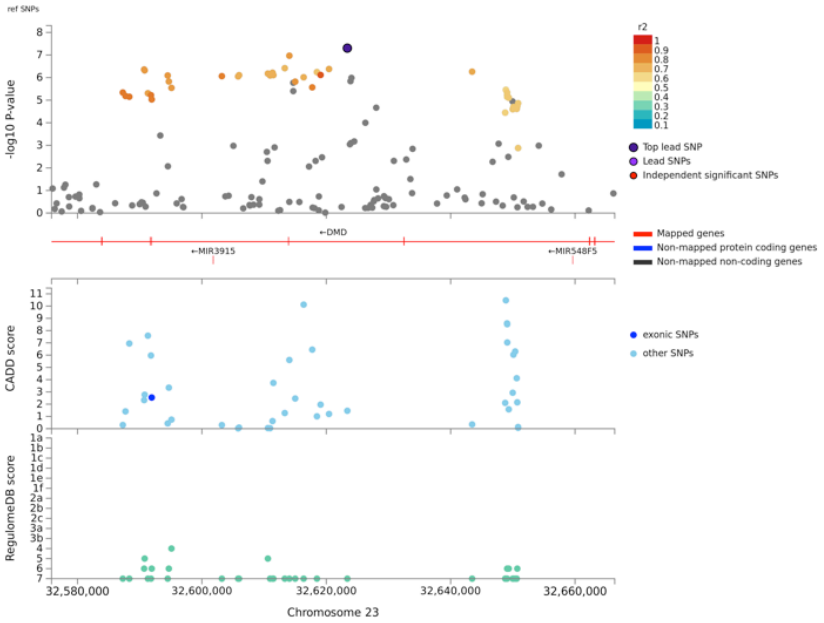

Supplementary Figure 6.4 *Annotated locus plot showing the CADD and RegulomeDB scores for one of the SNPs that reached standard genome-wide significance (p < 5e-8).*

Interaction Plot (rs5928040, DMD)

Females

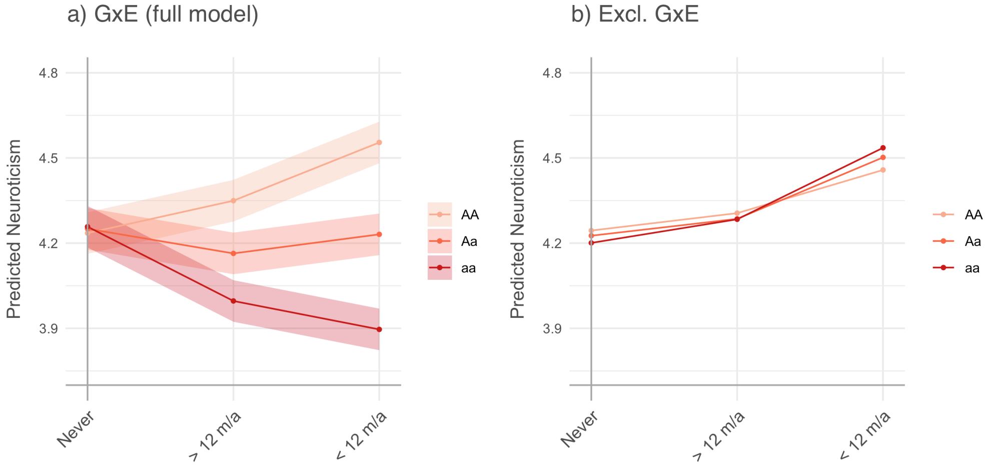

Supplementary Figure 6.5 *Average predicted neuroticism based on a) the full model (i.e., all main and interaction effects), with 95% confidence intervals for the GxE term, or b) everything except the SNP-environment interaction of interest (incl. all main effects and covariate interaction effects). Plot b will show any potential interaction effect captured by the covariates. (NOTE: Males and females were analysed together, but to do differences in the number alleles they were plotted separately).*

Males

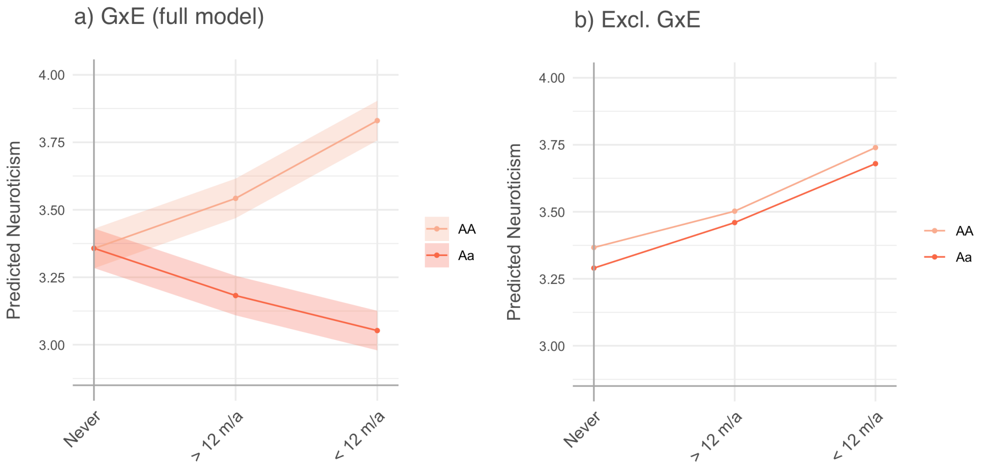

Supplementary Figure 6.6 *Average predicted neuroticism based on a) the full model (i.e., all main and interaction effects), with 95% confidence intervals for the GxE term, or b) everything except the SNP-environment interaction of interest (incl. all main effects and covariate interaction effects). Plot b will show any potential interaction effect captured by the covariates. (NOTE: Males and females were analysed together, but to do differences in the number alleles they were plotted separately).*

Gene Analysis

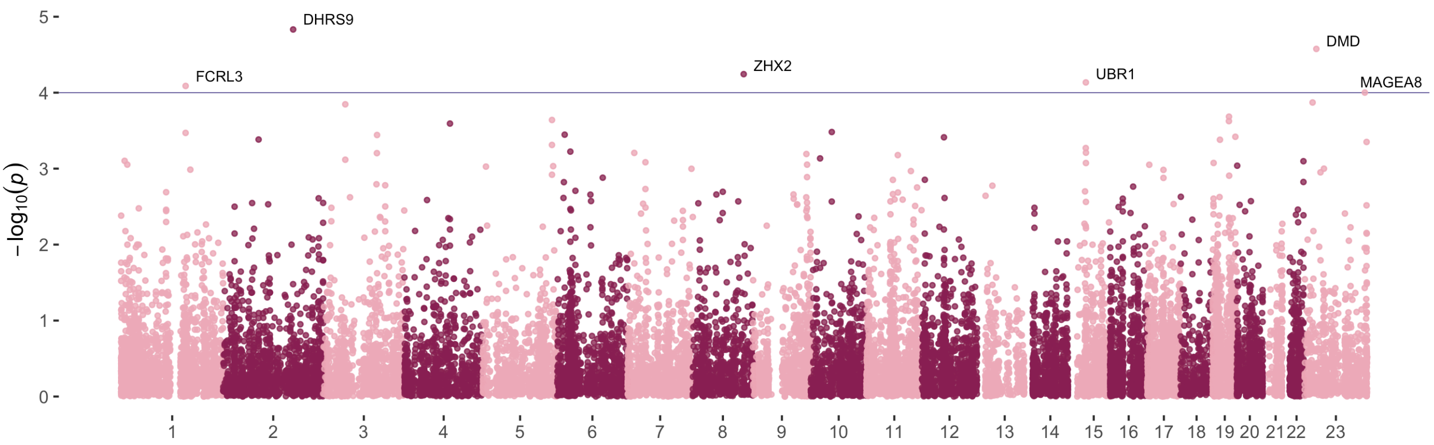

Supplementary Figure 6.7 *Manhattan plot showing the -log10 p-values for the MAGMA gene analysis (based on the GWEIS SNP-environment interaction results). Suggestively significant genes (p < 1e-4) have been annotated.*

Been in a Confiding Relationship as an Adult

GWEIS

Manhattan Plot

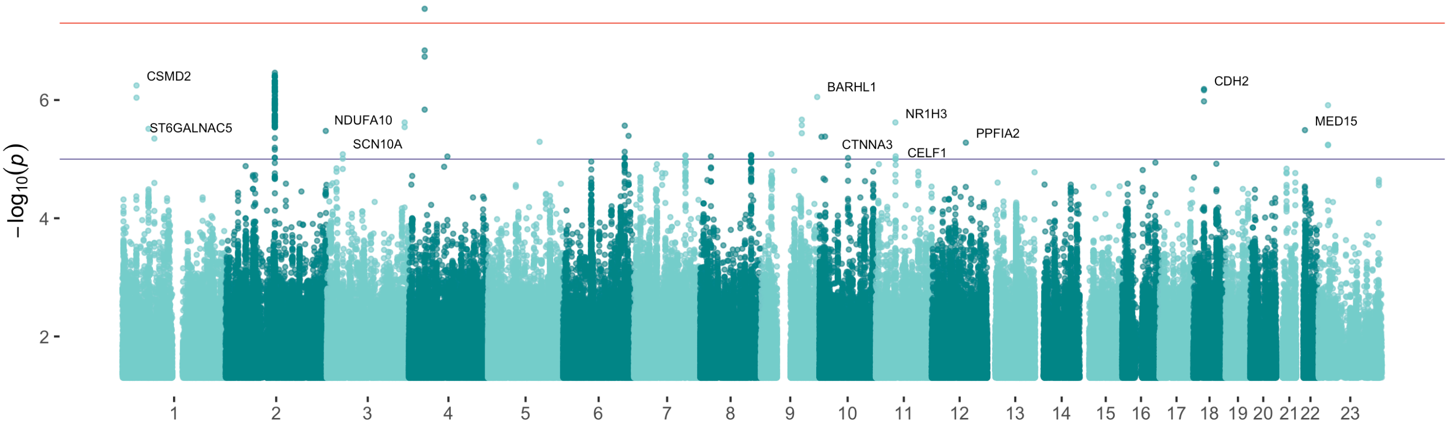

Supplementary Figure 7.1 *Manhattan plot showing the -log10 SNP-environment interaction p-values from the GWEIS. Gene labels have been annotated to suggestively significant (p < 1e-5) intragenic SNPs with the lowest p-value. (Only SNPs with p < .05 were plotted).*

Locus Plot

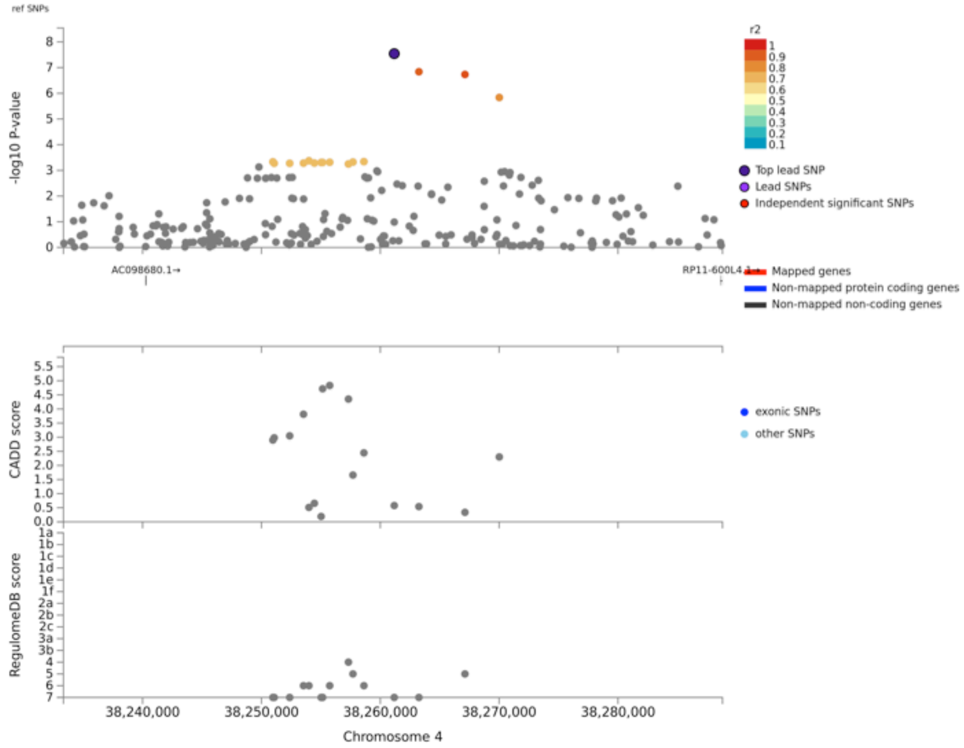

Supplementary Figure 7.2 *Annotated locus plot showing the CADD and RegulomeDB scores for the SNP that reached standard genome-wide significance (p < 5e-8).*

Interaction Plots

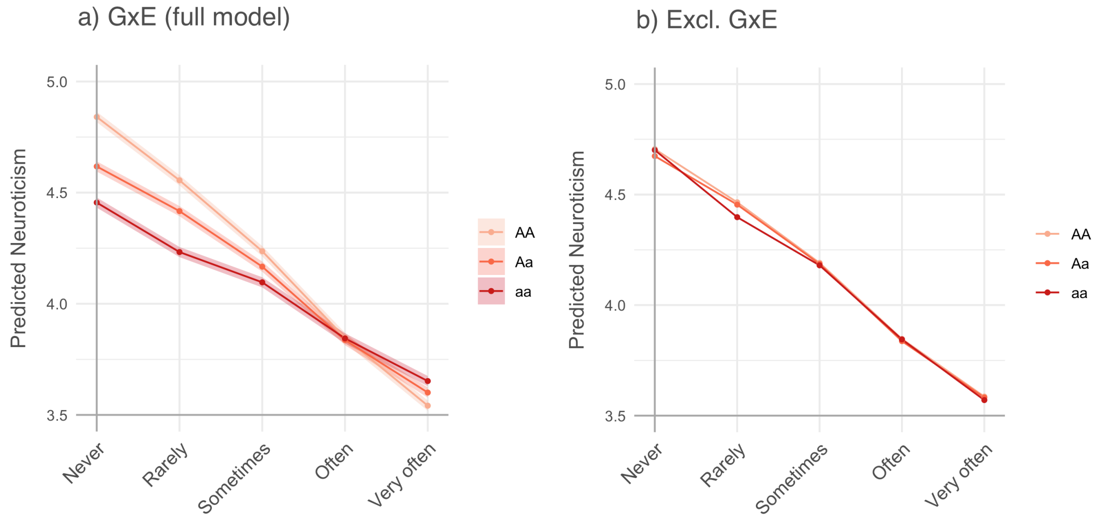

Supplementary Figure 7.3 *Average predicted neuroticism based on a) the full model (i.e., all main and interaction effects), with 95% confidence intervals for the GxE term, or b) everything except the SNP-environment interaction of interest (incl. all main effects and covariate interaction effects). Plot b will show any potential interaction effect captured by the covariates.*

Gene Analysis

Manhattan Plot

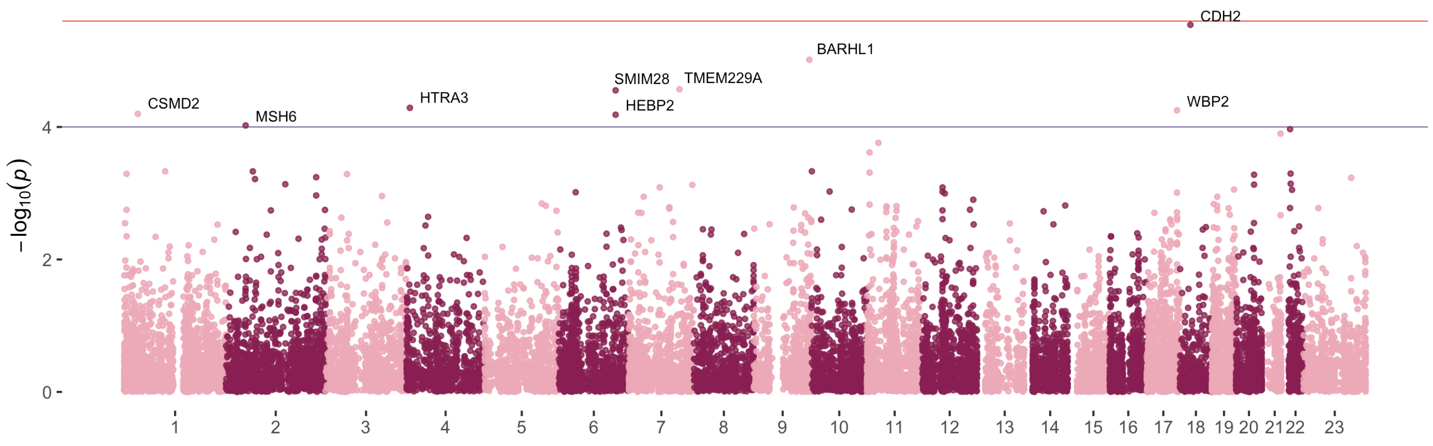

Supplementary Figure 7.4 *Manhattan plot showing the -log10 p-values for the MAGMA gene analysis (based on the GWEIS SNP-environment interaction results). Suggestively significant genes (p < 1e-4) have been annotated.*

Alcohol Intake Frequency

GWEIS

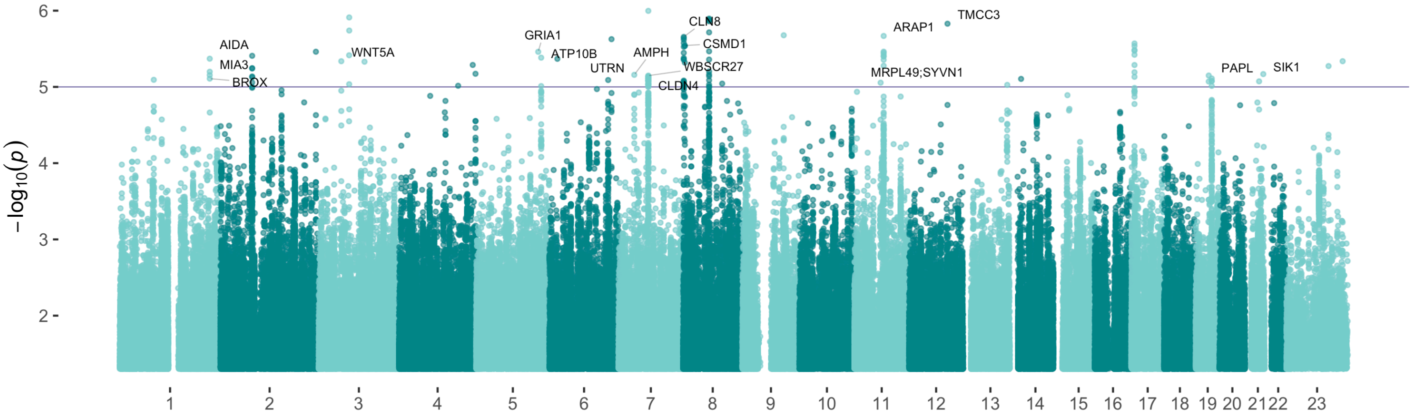

Supplementary Figure 8.1 *Manhattan plot showing the -log10 SNP-environment interaction p-values from the GWEIS. Gene labels have been annotated to suggestively significant (p < 1e-5) intragenic SNPs with the lowest p-value. (Only SNPs with p < .05 were plotted).*

Gene Analysis

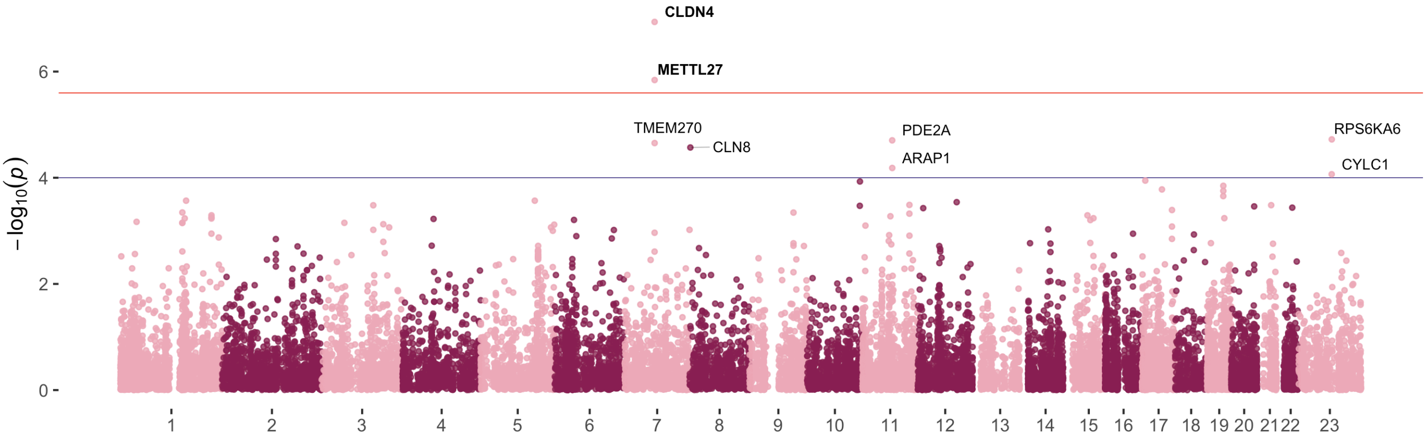

Supplementary Figure 8.2 *Manhattan plot showing the -log10 p-values for the MAGMA gene analysis (based on the GWEIS SNP-environment interaction results). Suggestively significant genes (p < 1e-4) have been annotated.*

Victim of Sexual Assault

GWEIS

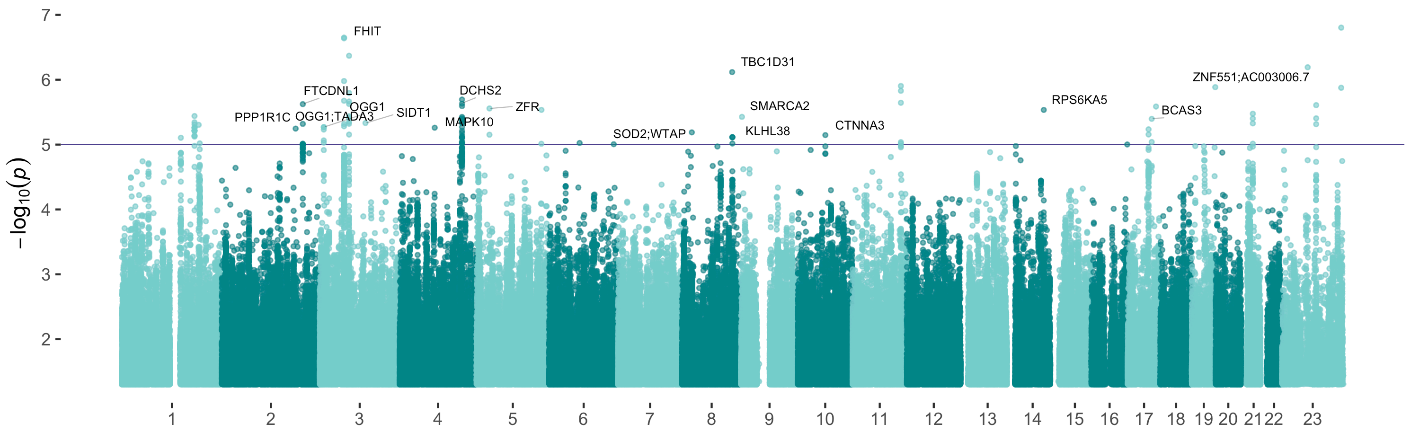

Supplementary Figure 9.1 *Manhattan plot showing the -log10 SNP-environment interaction p-values from the GWEIS. Gene labels have been annotated to suggestively significant (p < 1e-5) intragenic SNPs with the lowest p-value. (Only SNPs with p < .05 were plotted).*

Gene Analysis

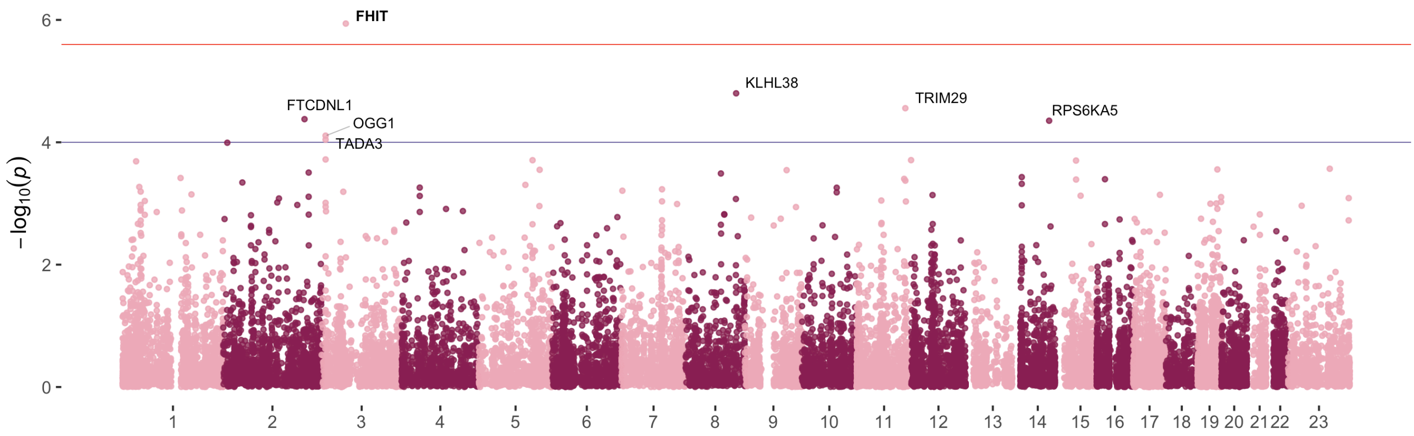

Supplementary Figure 9.2 *Manhattan plot showing the -log10 p-values for the MAGMA gene analysis (based on the GWEIS SNP-environment interaction results). Suggestively significant genes (p < 1e-4) have been annotated.*

Chronic Pain

GWEIS

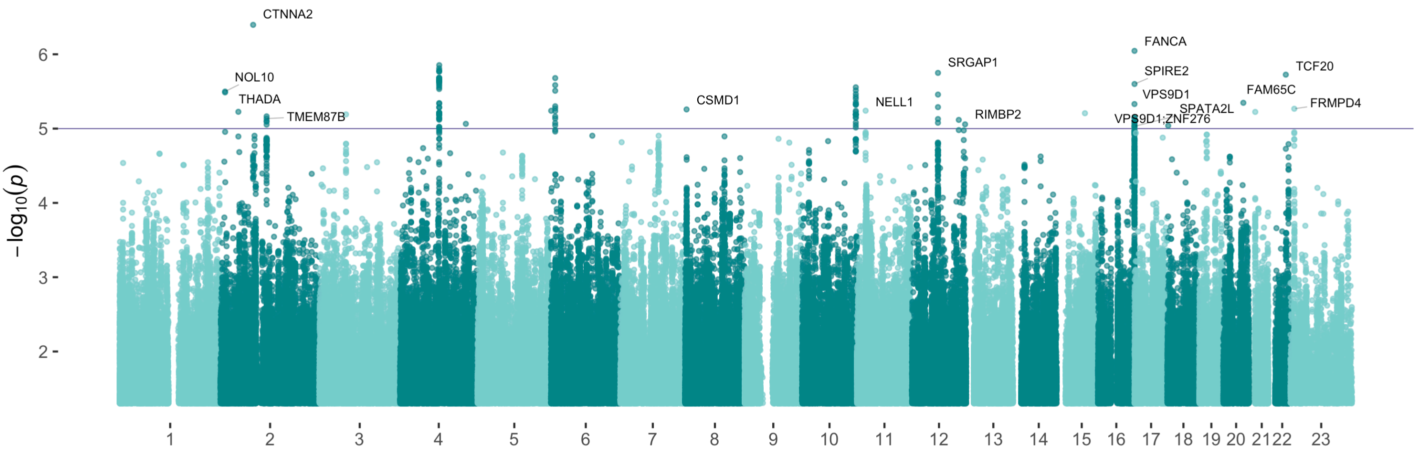

Supplementary Figure 10.1 *Manhattan plot showing the -log10 SNP-environment interaction p-values from the GWEIS. Gene labels have been annotated to suggestively significant (p < 1e-5) intragenic SNPs with the lowest p-value. (Only SNPs with p < .05 were plotted).*

Gene Analysis

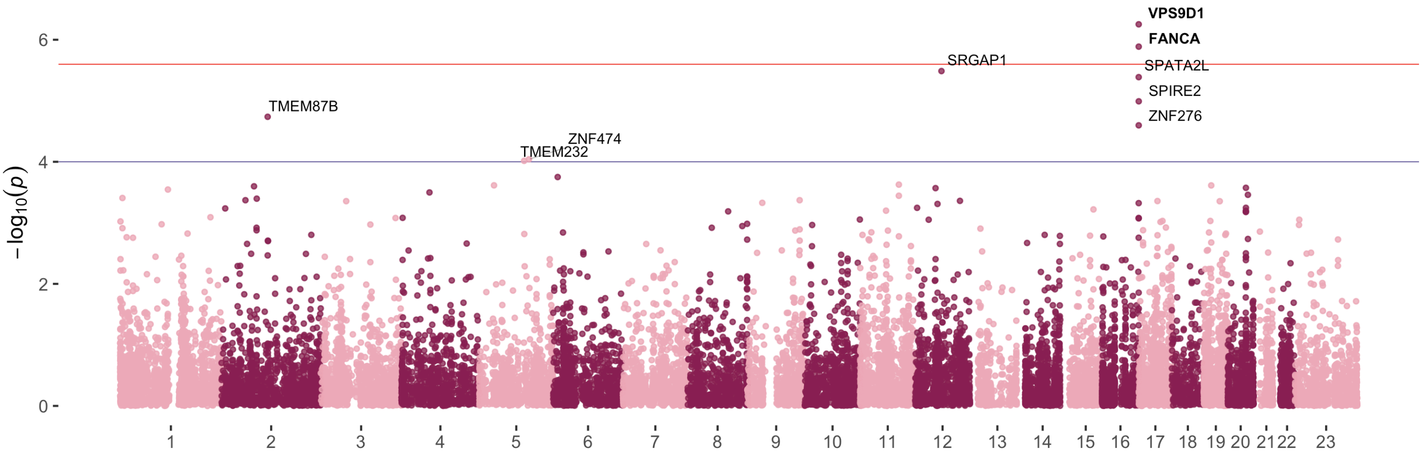

Supplementary Figure 10.2 *Manhattan plot showing the -log10 p-values for the MAGMA gene analysis (based on the GWEIS SNP-environment interaction results). Suggestively significant genes (p < 1e-4) have been annotated.*

Age Completed Full-Time Education

GWEIS

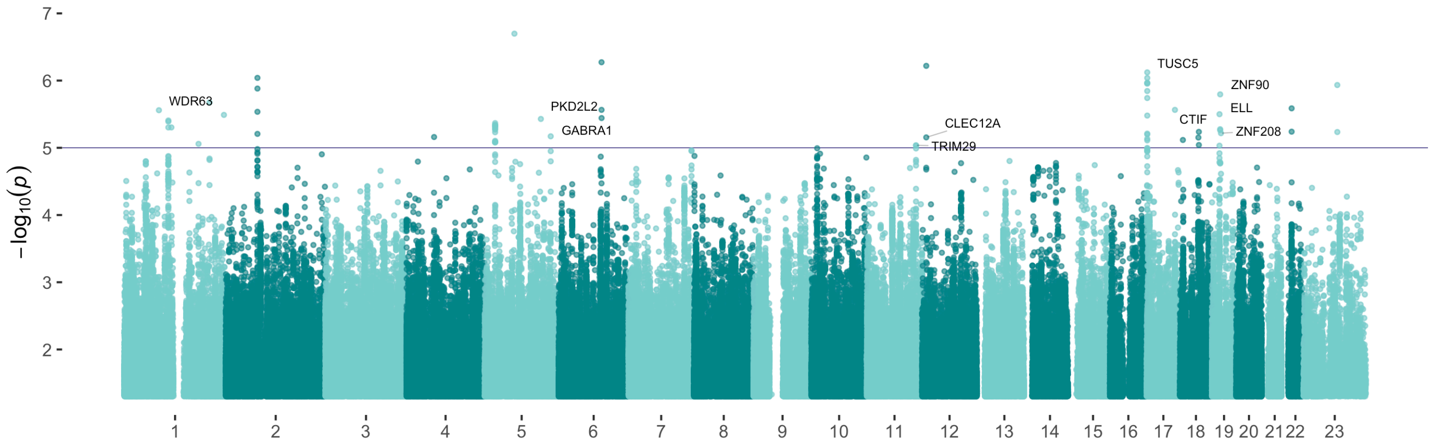

Supplementary Figure 11.1*: Manhattan plot showing the -log10 SNP-environment interaction p-values from the GWEIS. Gene labels have been annotated to suggestively significant (p < 1e-5) intragenic SNPs with the lowest p-value. (Only SNPs with p < .05 were plotted).*

Gene Analysis

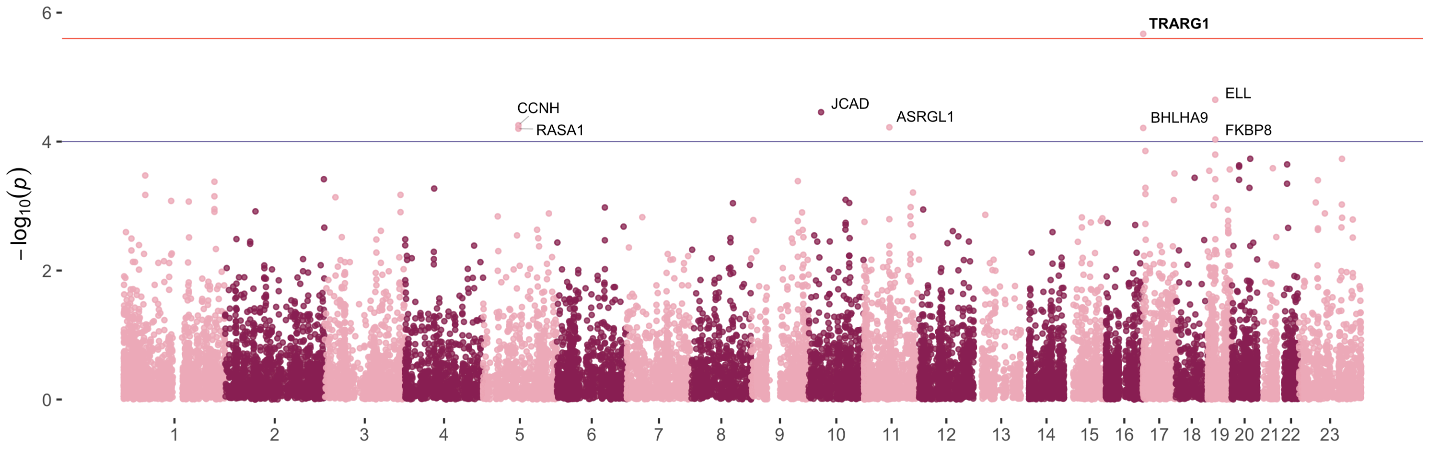

Supplementary Figure 11.2 *Manhattan plot showing the -log10 p-values for the MAGMA gene analysis (based on the GWEIS SNP-environment interaction results). Suggestively significant genes (p < 1e-4) have been annotated.*

Family Relationship Satisfaction

GWEIS

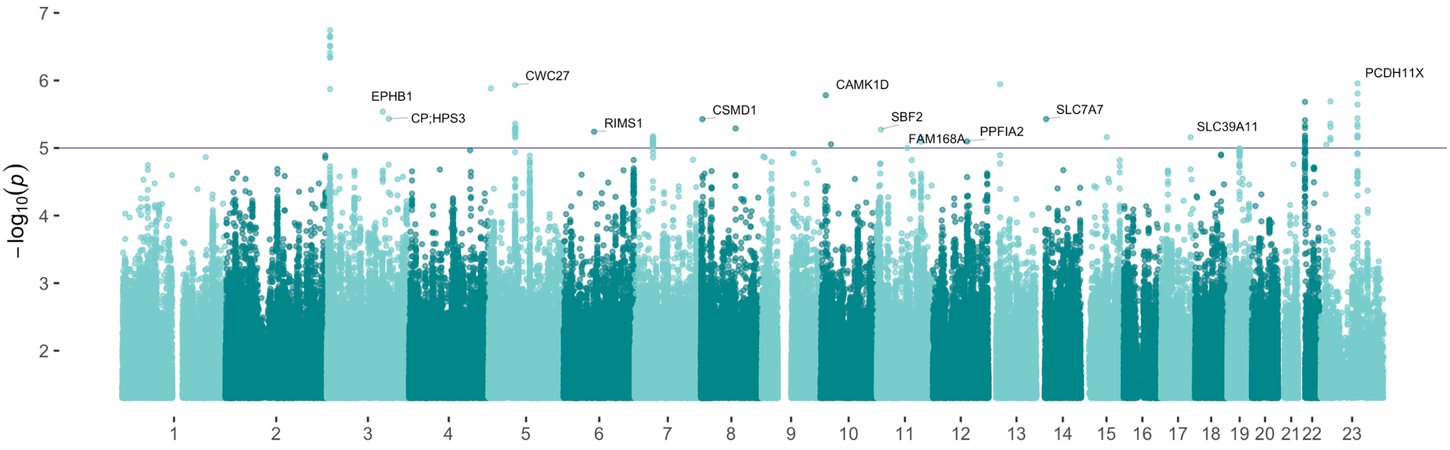

Supplementary Figure 12.1 *Manhattan plot showing the -log10 SNP-environment interaction p-values from the GWEIS. Gene labels have been annotated to suggestively significant (p < 1e-5) intragenic SNPs with the lowest p-value. (Only SNPs with p < .05 were plotted).*

Gene Analysis

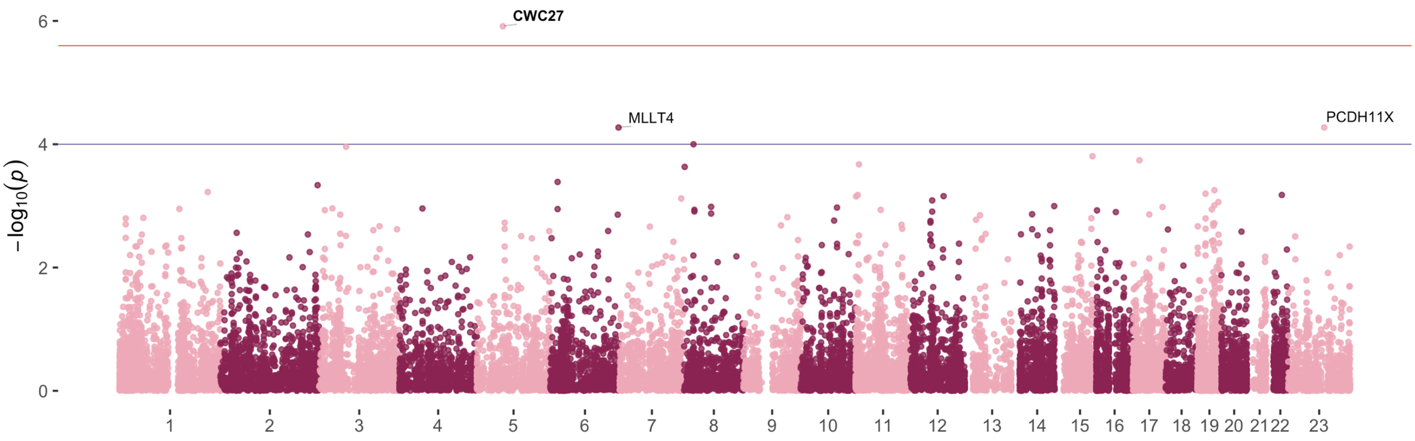

Supplementary Figure 12.2 *Manhattan plot showing the -log10 p-values for the MAGMA gene analysis (based on the GWEIS SNP-environment interaction results). Suggestively significant genes (p < 1e-4) have been annotated.*

Fluid Intelligence Score

GWEIS

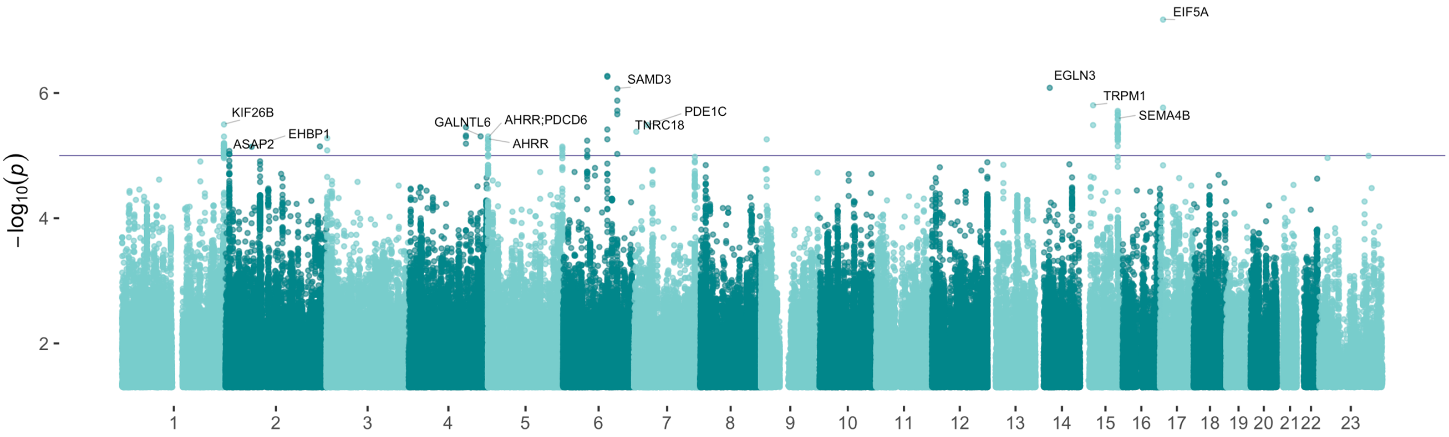

Supplementary Figure 13.1 *Manhattan plot showing the -log10 SNP-environment interaction p-values from the GWEIS. Gene labels have been annotated to suggestively significant (p < 1e-5) intragenic SNPs with the lowest p-value. (Only SNPs with p < .05 were plotted).*

Gene Analysis

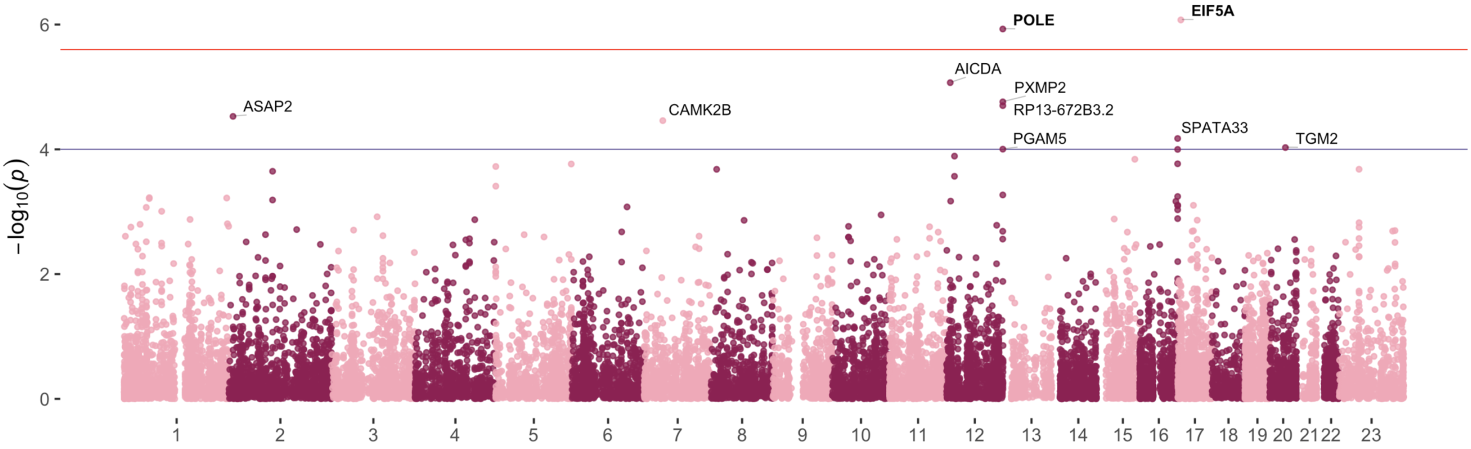

Supplementary Figure 13.2 *Manhattan plot showing the -log10 p-values for the MAGMA gene analysis (based on the GWEIS SNP-environment interaction results). Suggestively significant genes (p < 1e-4) have been annotated.*

Frequency of Friend/Family Visits

GWEIS

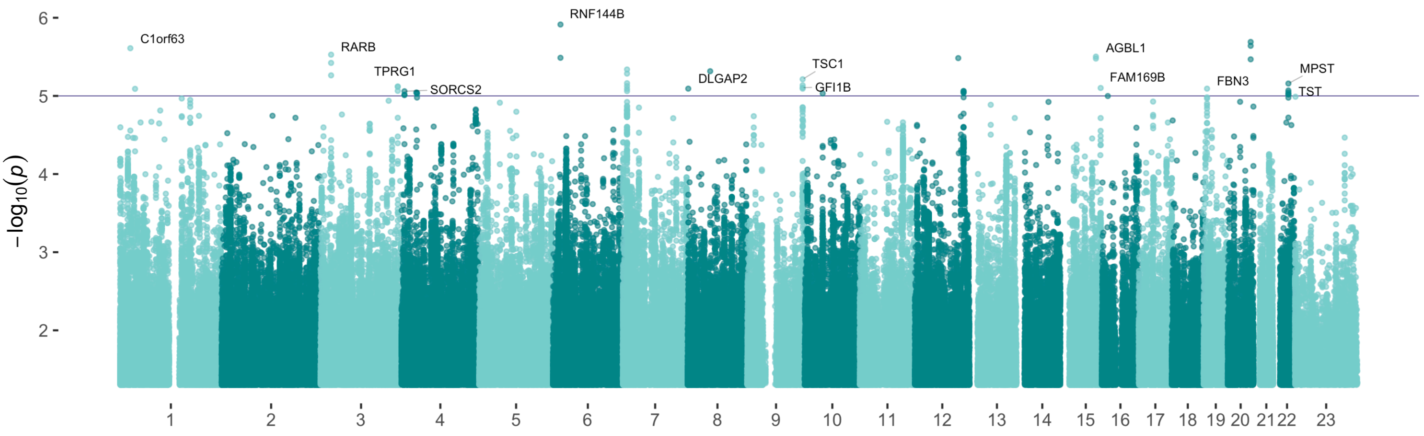

Supplementary Figure 14.1 *Manhattan plot showing the -log10 SNP-environment interaction p-values from the GWEIS. Gene labels have been annotated to suggestively significant (p < 1e-5) intragenic SNPs with the lowest p-value. (Only SNPs with p < .05 were plotted).*

Gene Analysis

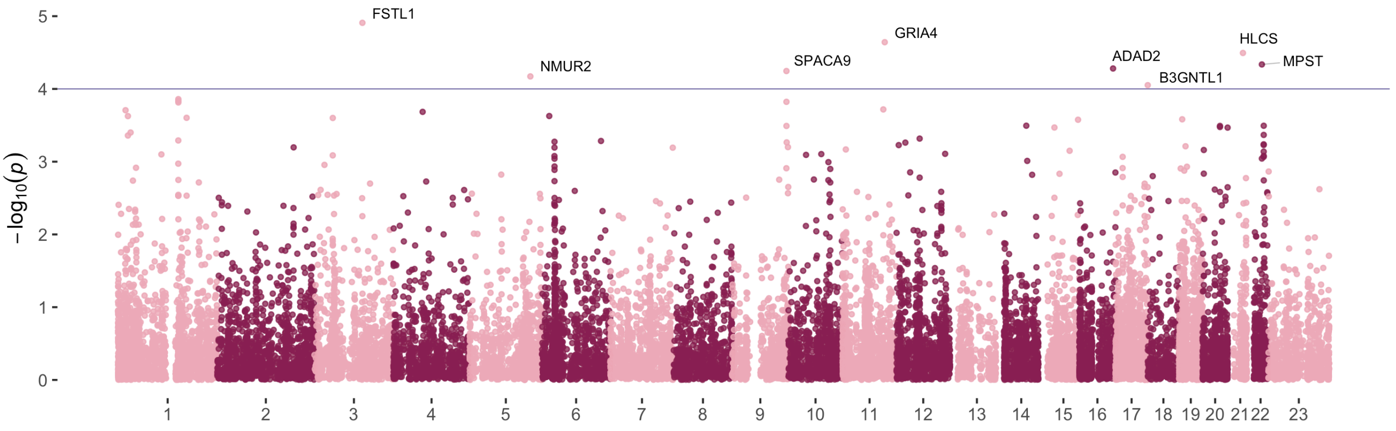

Supplementary Figure 14.2 *Manhattan plot showing the -log10 p-values for the MAGMA gene analysis (based on the GWEIS SNP-environment interaction results). Suggestively significant genes (p < 1e-4) have been annotated.*

Sleeplessness / Insomnia

GWEIS

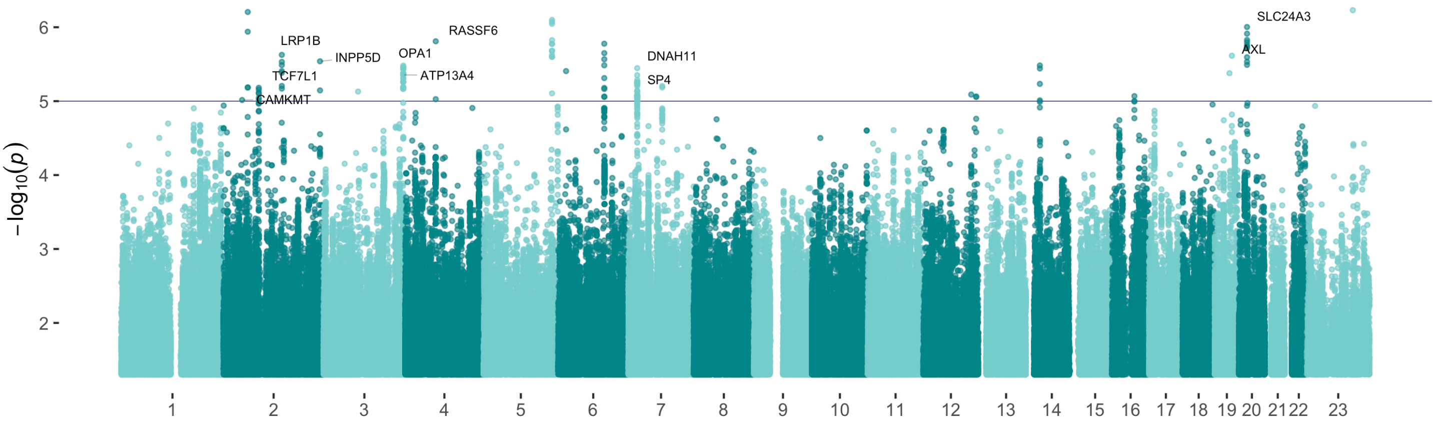

Supplementary Figure 15.1 *Manhattan plot showing the -log10 SNP-environment interaction p-values from the GWEIS. Gene labels have been annotated to suggestively significant (p < 1e-5) intragenic SNPs with the lowest p-value. (Only SNPs with p < .05 were plotted).*

Gene Analysis

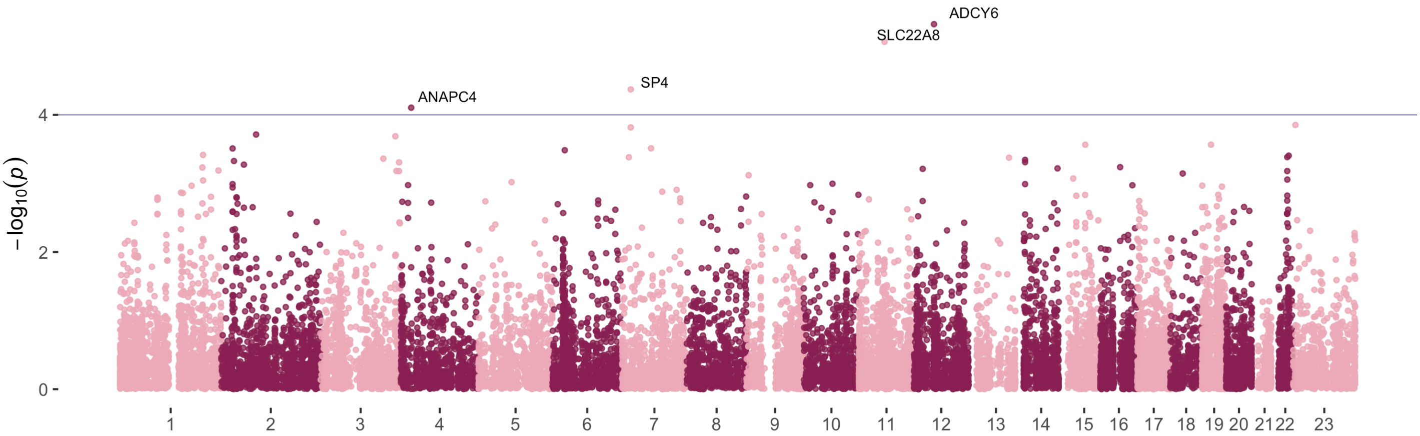

Supplementary Figure 15.2 *Manhattan plot showing the -log10 p-values for the MAGMA gene analysis (based on the GWEIS SNP-environment interaction results). Suggestively significant genes (p < 1e-4) have been annotated.*

Long-Standing Illness, Disability, or Infirmity

GWEIS

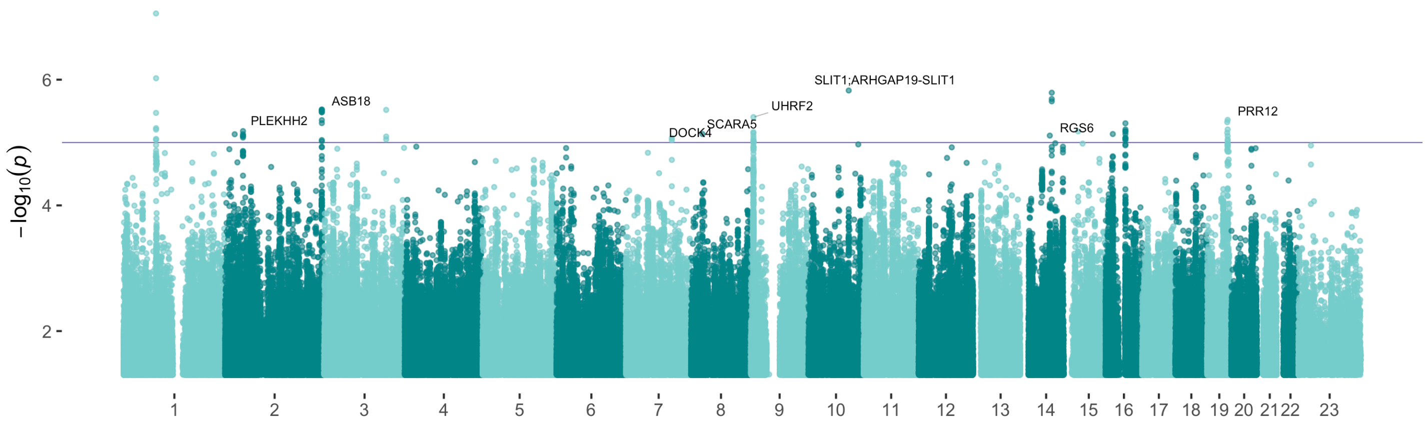

Supplementary Figure 16.1 *Manhattan plot showing the -log10 SNP-environment interaction p-values from the GWEIS. Gene labels have been annotated to suggestively significant (p < 1e-5) intragenic SNPs with the lowest p-value. (Only SNPs with p < .05 were plotted).*

Gene Analysis

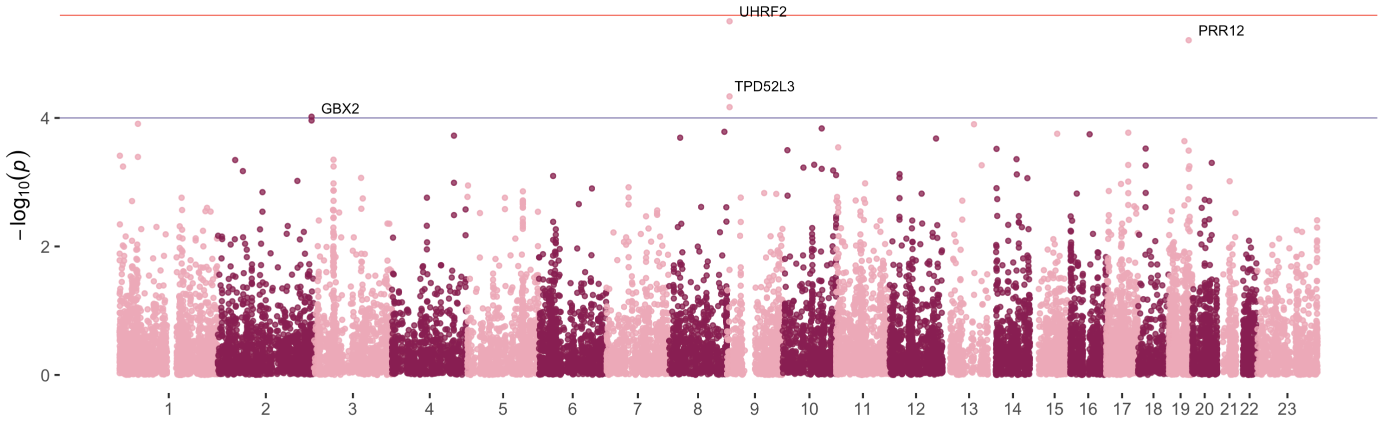

Supplementary Figure 16.2 *Manhattan plot showing the -log10 p-values for the MAGMA gene analysis (based on the GWEIS SNP-environment interaction results). Suggestively significant genes (p < 1e-4) have been annotated.*

Health Satisfaction

GWEIS

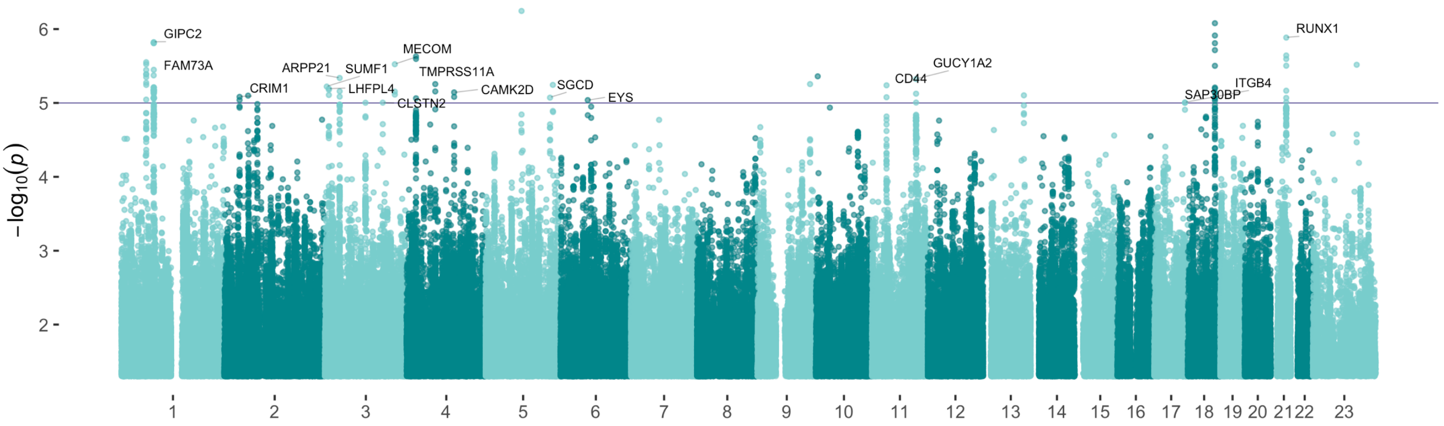

Supplementary Figure 17.1 *Manhattan plot showing the -log10 SNP-environment interaction p-values from the GWEIS. Gene labels have been annotated to suggestively significant (p < 1e-5) intragenic SNPs with the lowest p-value. (Only SNPs with p < .05 were plotted).*

Gene Analysis

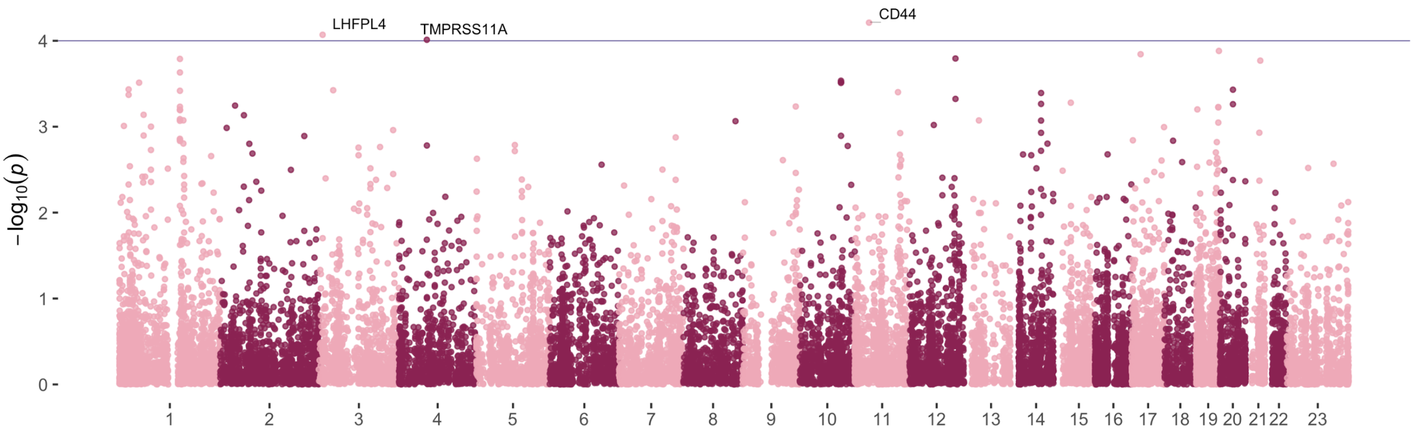

Supplementary Figure 17.2 *Manhattan plot showing the -log10 p-values for the MAGMA gene analysis (based on the GWEIS SNP-environment interaction results). Suggestively significant genes (p < 1e-4) have been annotated.).*

Friendships Satisfaction

GWEIS

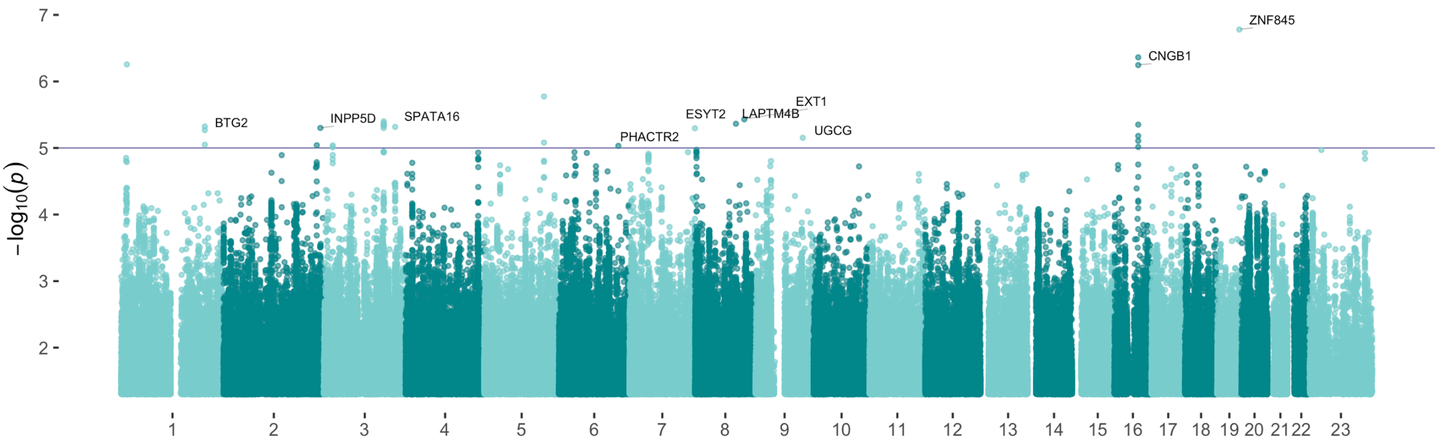

Supplementary Figure 18.1 *Manhattan plot showing the -log10 SNP-environment interaction p-values from the GWEIS. Gene labels have been annotated to suggestively significant (p < 1e-5) intragenic SNPs with the lowest p-value. (Only SNPs with p < .05 were plotted).*

Gene Analysis

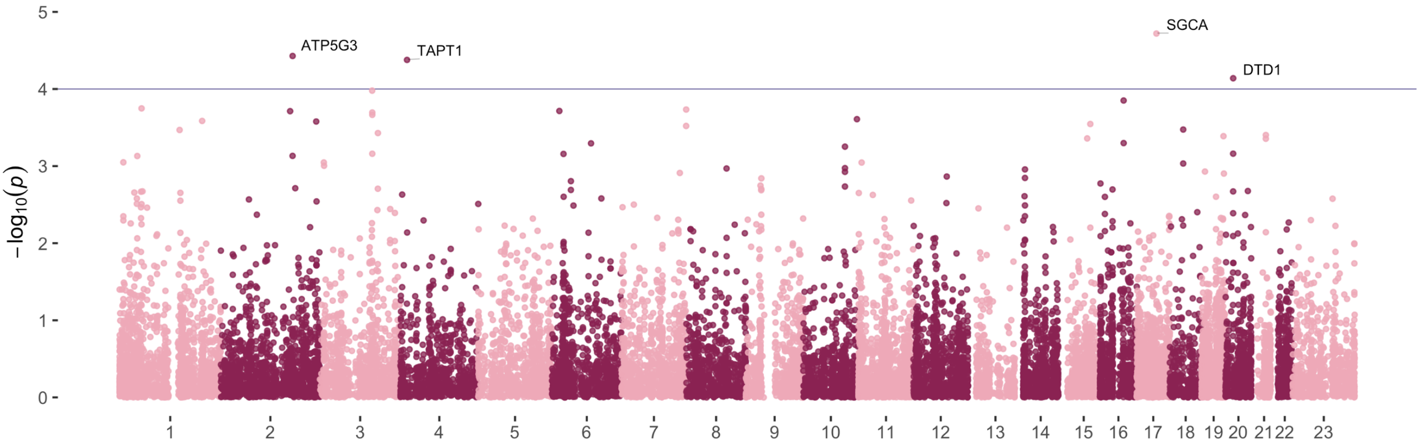

Supplementary Figure 18.2 *Manhattan plot showing the -log10 p-values for the MAGMA gene analysis (based on the GWEIS SNP-environment interaction results). Suggestively significant genes (p < 1e-4) have been annotated.*

Financial Situation Satisfaction

GWEIS

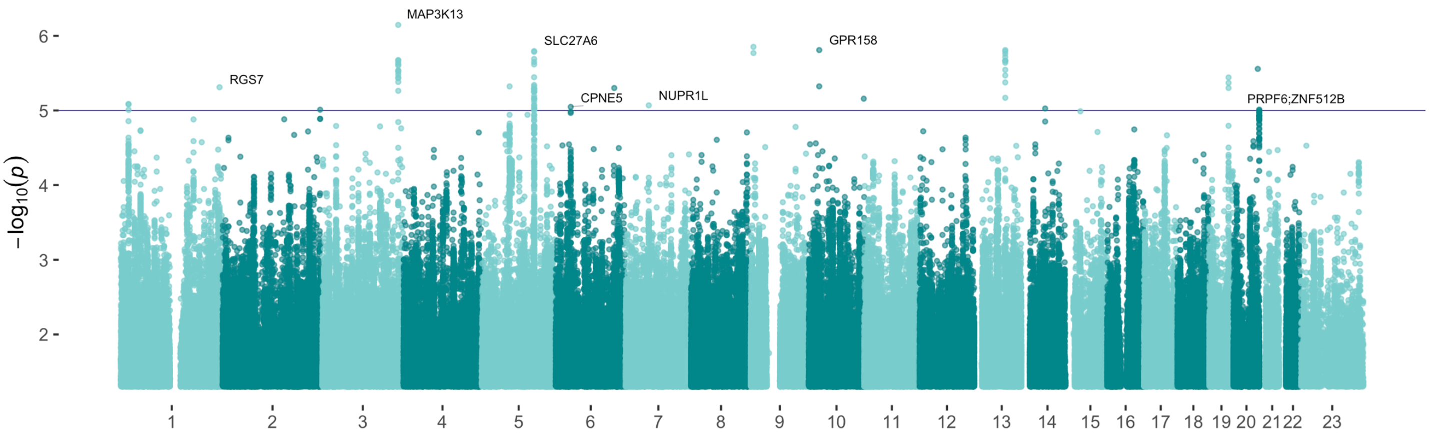

Supplementary Figure 19.1 *Manhattan plot showing the -log10 SNP-environment interaction p-values from the GWEIS. Gene labels have been annotated to suggestively significant (p < 1e-5) intragenic SNPs with the lowest p-value. (Only SNPs with p < .05 were plotted).*

Gene Analysis

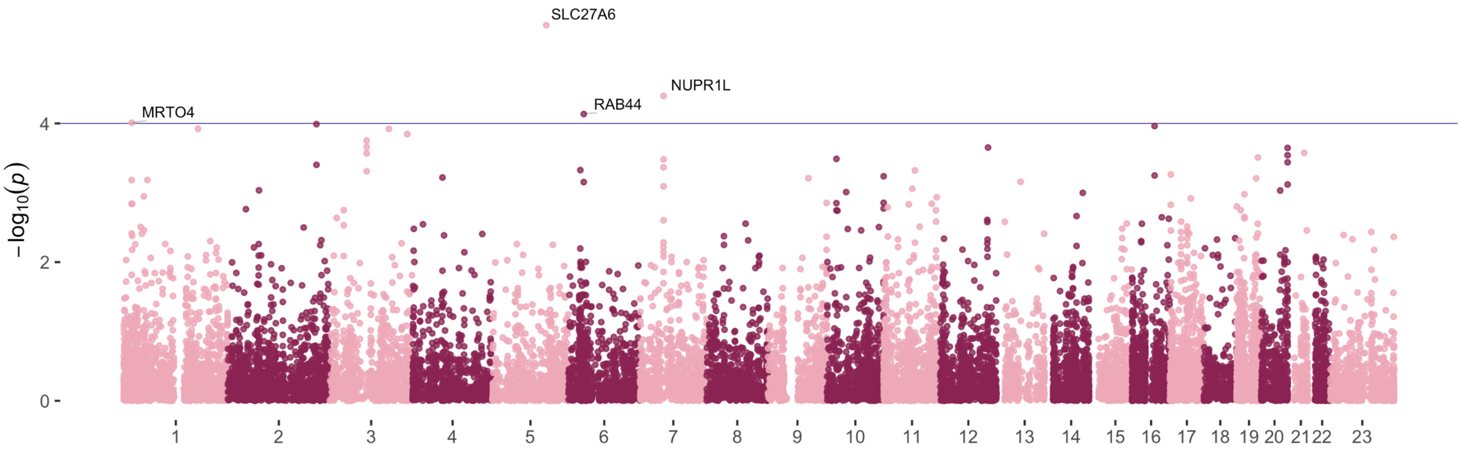

Supplementary Figure 19.2 *Manhattan plot showing the -log10 p-values for the MAGMA gene analysis (based on the GWEIS SNP-environment interaction results). Suggestively significant genes (p < 1e-4) have been annotated.*

Illness, Injury, Bereavement, Stress in the Last 2 years

GWEIS

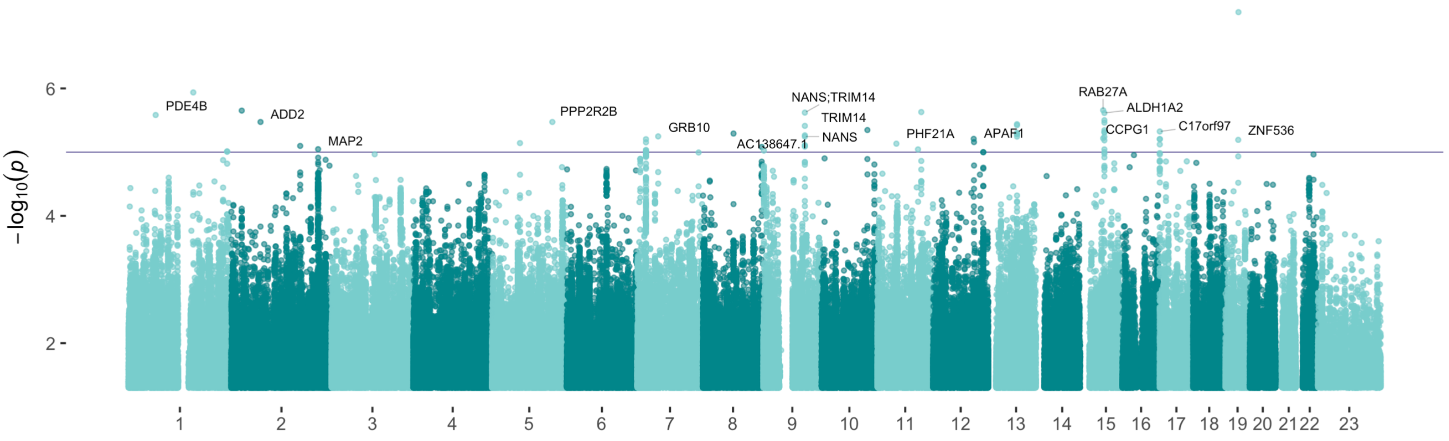

Supplementary Figure 20.1 *Manhattan plot showing the -log10 SNP-environment interaction p-values from the GWEIS. Gene labels have been annotated to suggestively significant (p < 1e-5) intragenic SNPs with the lowest p-value. (Only SNPs with p < .05 were plotted).*

Gene Analysis

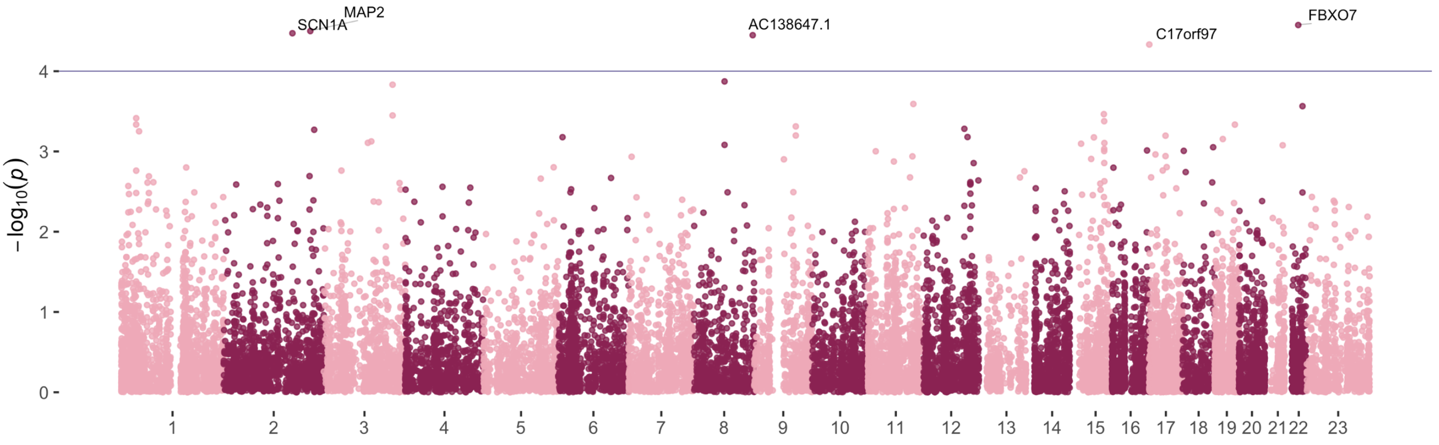

Supplementary Figure 20.2 *Manhattan plot showing the -log10 p-values for the MAGMA gene analysis (based on the GWEIS SNP-environment interaction results). Suggestively significant genes (p < 1e-4) have been annotated.*

Smoking Status

GWEIS

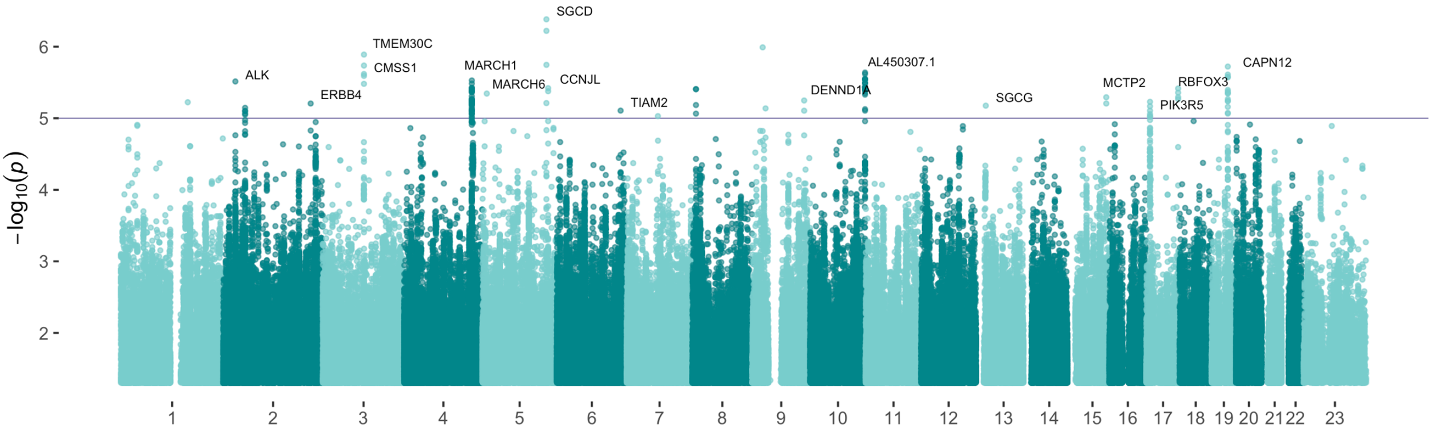

Supplementary Figure 21.1 *Manhattan plot showing the -log10 SNP-environment interaction p-values from the GWEIS. Gene labels have been annotated to suggestively significant (p < 1e-5) intragenic SNPs with the lowest p-value. (Only SNPs with p < .05 were plotted).*

Gene Analysis

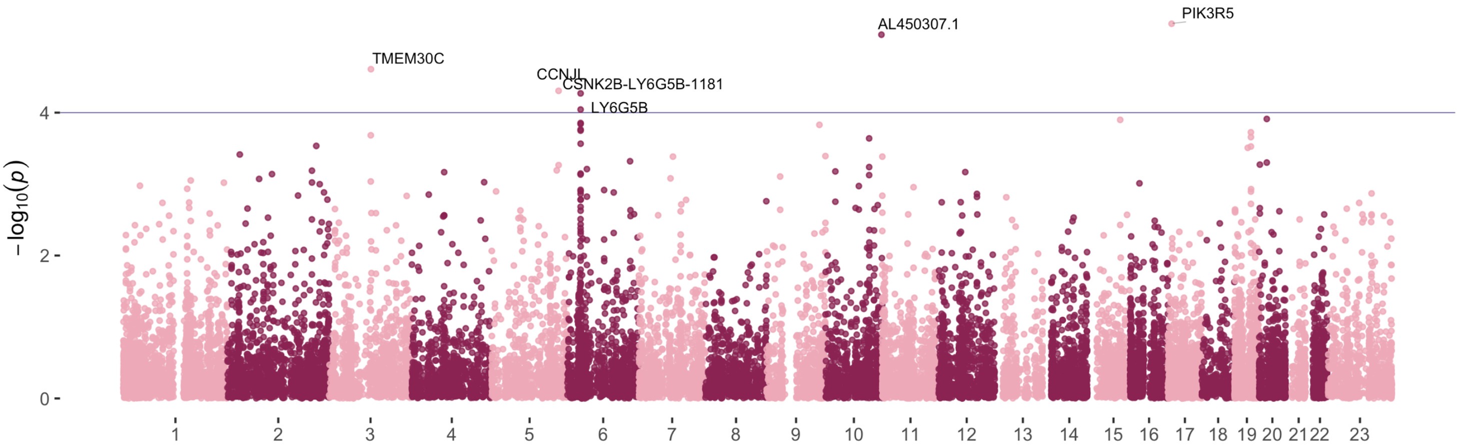

Supplementary Figure 21.2 *Manhattan plot showing the -log10 p-values for the MAGMA gene analysis (based on the GWEIS SNP-environment interaction results). Suggestively significant genes (p < 1e-4) have been annotated.*

Physically Abused by Family as a Child

GWEIS

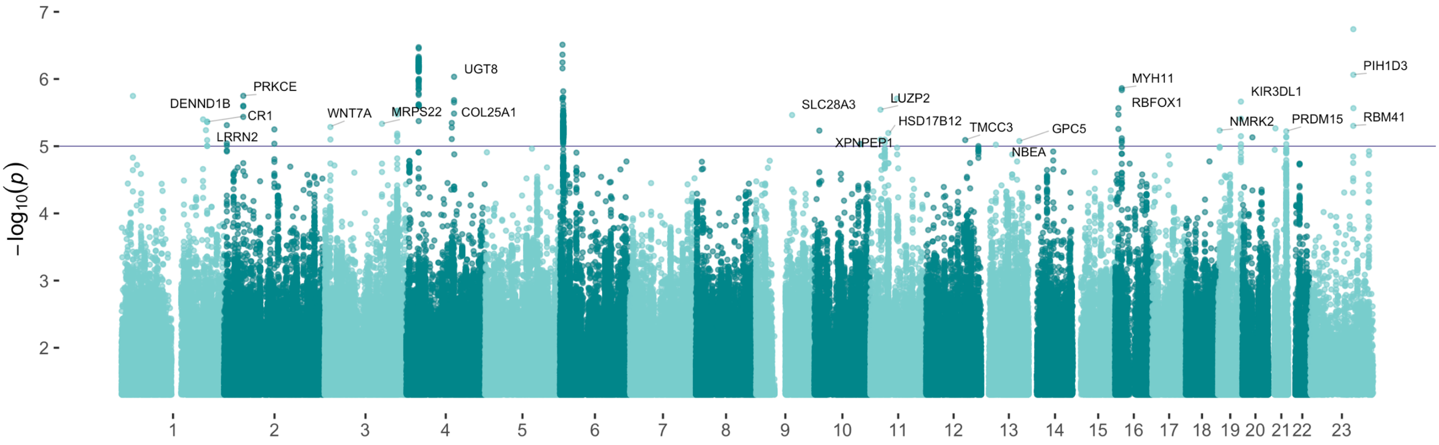

Supplementary Figure 22.1 *Manhattan plot showing the -log10 SNP-environment interaction p-values from the GWEIS. Gene labels have been annotated to suggestively significant (p < 1e-5) intragenic SNPs with the lowest p-value. (Only SNPs with p < .05 were plotted).*

Gene Analysis

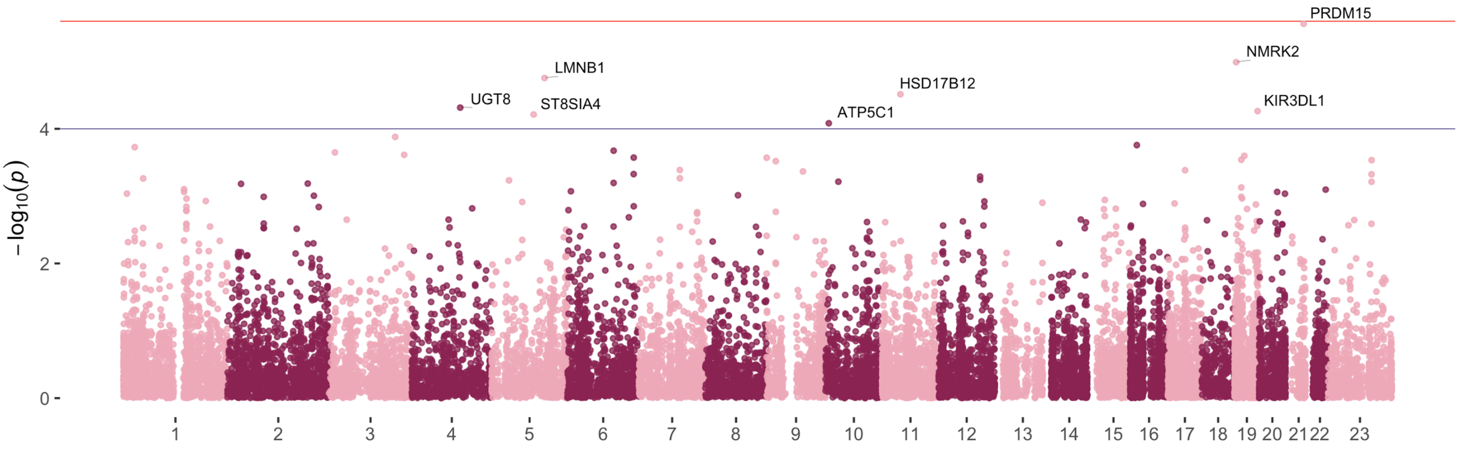

Supplementary Figure 22.2 *Manhattan plot showing the -log10 p-values for the MAGMA gene analysis (based on the GWEIS SNP-environment interaction results). Suggestively significant genes (p < 1e-4) have been annotated.*

Felt Loved as a Child

GWEIS

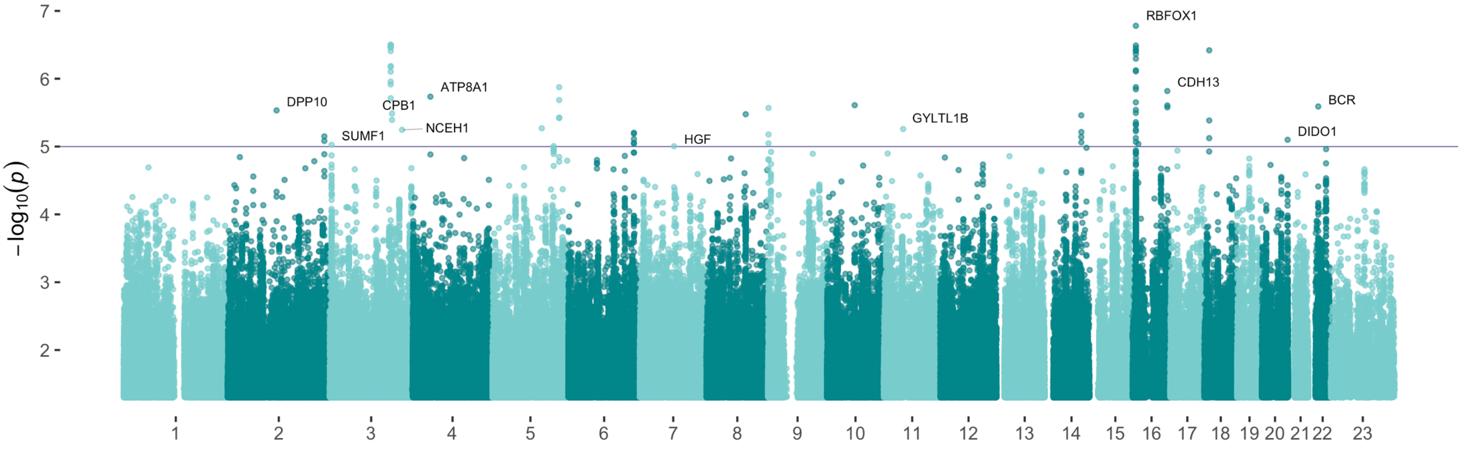

Supplementary Figure 23.1 *Manhattan plot showing the -log10 SNP-environment interaction p-values from the GWEIS. Gene labels have been annotated to suggestively significant (p < 1e-5) intragenic SNPs with the lowest p-value. (Only SNPs with p < .05 were plotted).*

Gene Analysis

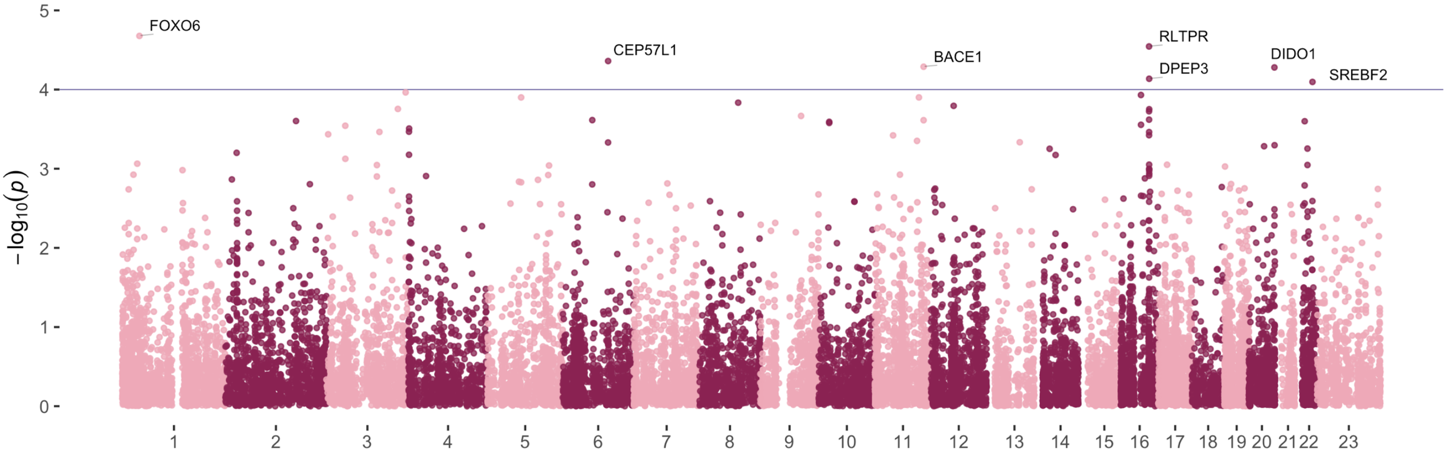

Supplementary Figure 23.2 *Manhattan plot showing the -log10 p-values for the MAGMA gene analysis (based on the GWEIS SNP-environment interaction results). Suggestively significant genes (p < 1e-4) have been annotated.*

Victim of Physically Violent Crime

GWEIS

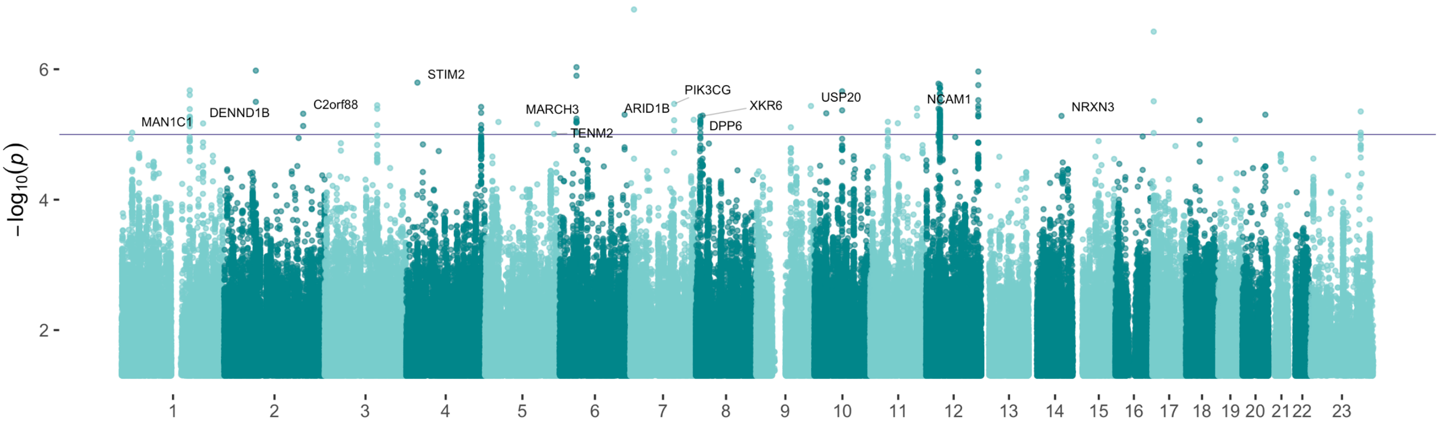

Supplementary Figure 24.1 *Manhattan plot showing the -log10 SNP-environment interaction p-values from the GWEIS. Gene labels have been annotated to suggestively significant (p < 1e-5) intragenic SNPs with the lowest p-value. (Only SNPs with p < .05 were plotted).*

Gene Analysis

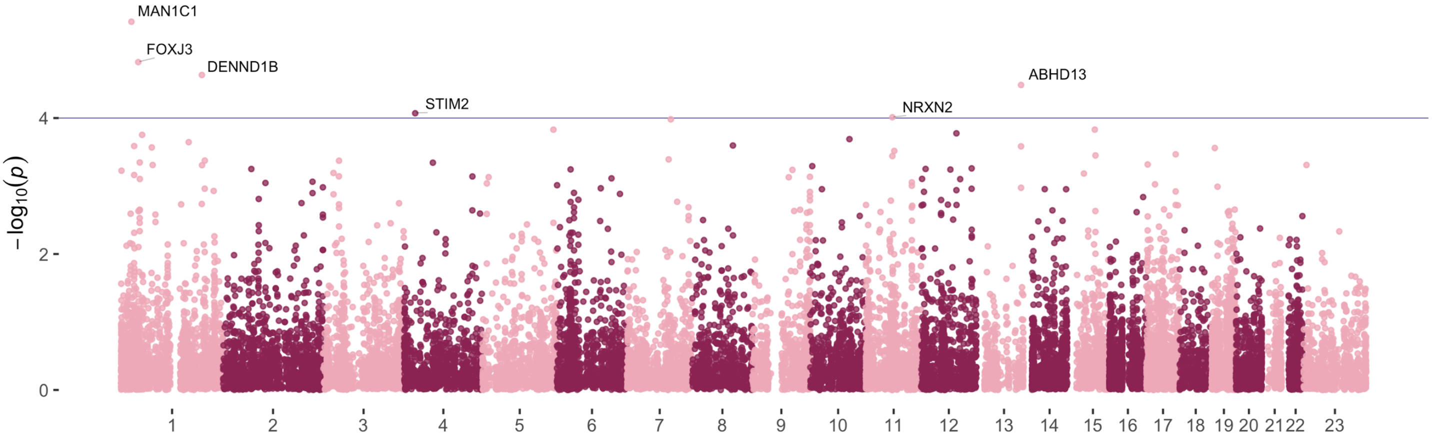

Supplementary Figure 24.2 *Manhattan plot showing the -log10 p-values for the MAGMA gene analysis (based on the GWEIS SNP-environment interaction results). Suggestively significant genes (p < 1e-4) have been annotated.*

Body Mass Index (BMI)

GWEIS

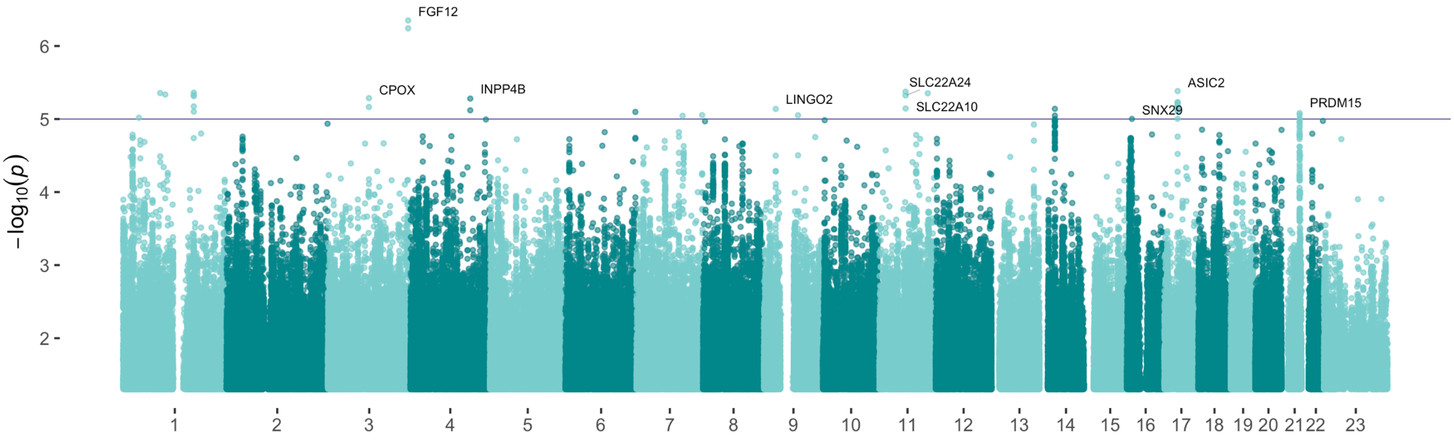

Supplementary Figure 25.1 *Manhattan plot showing the -log10 SNP-environment interaction p-values from the GWEIS. Gene labels have been annotated to suggestively significant (p < 1e-5) intragenic SNPs with the lowest p-value. (Only SNPs with p < .05 were plotted).*

Gene Analysis

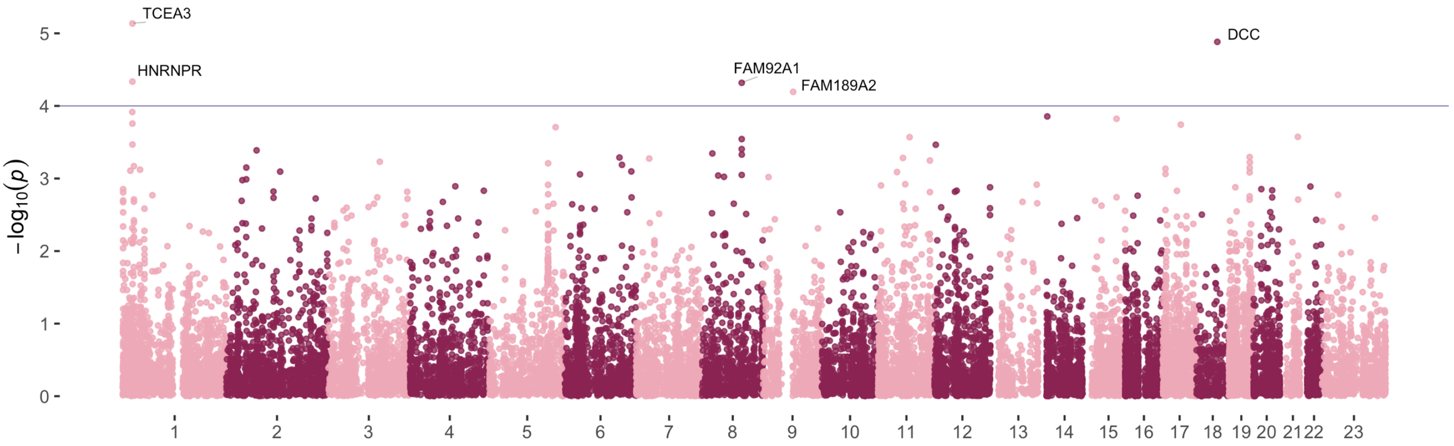

Supplementary Figure 25.2 *Manhattan plot showing the -log10 p-values for the MAGMA gene analysis (based on the GWEIS SNP-environment interaction results). Suggestively significant genes (p < 1e-4) have been annotated.*

MANHATTAN PLOTS (grouped)

This section contains all Manhattan plots from the GWEIS and gene analyses grouped together

GWEIS

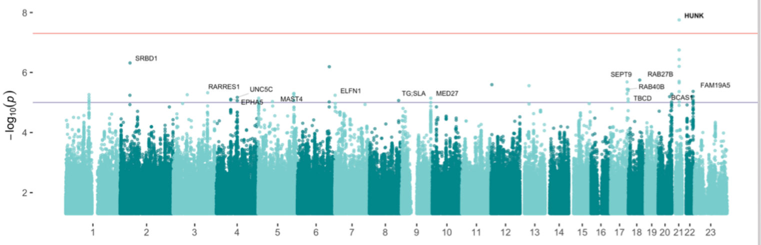

Townsend deprivation index at recruitment

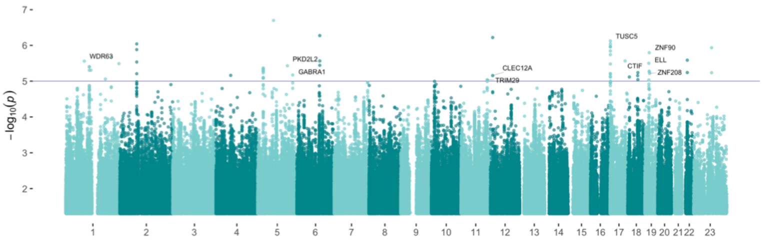

Age completed full time education

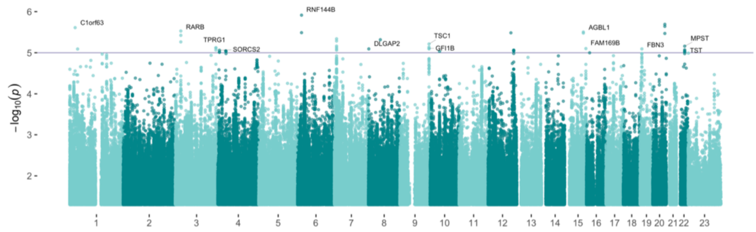

Frequency of friend/family visits

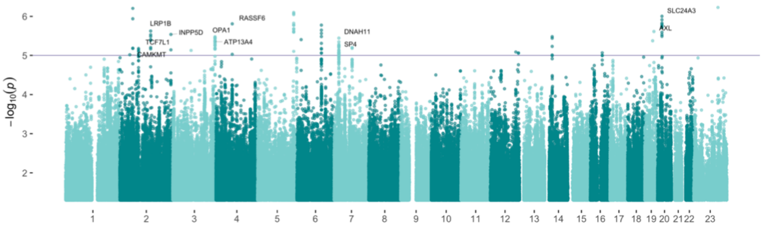

Sleeplessness / insomnia

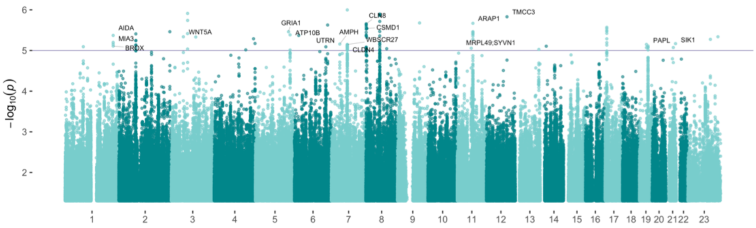

Alcohol intake frequency

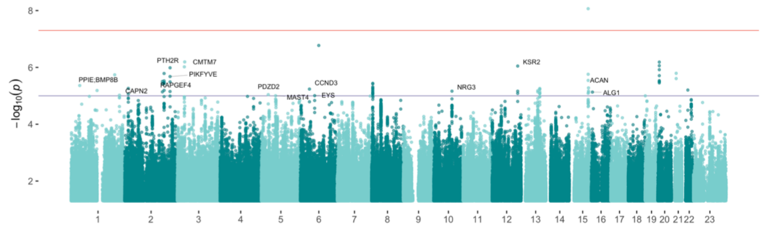

Able to confide

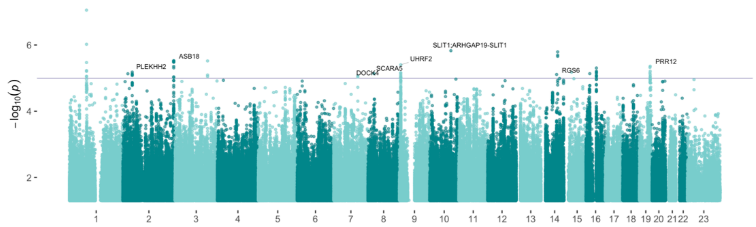

Long-standing illness, disability or infirmity

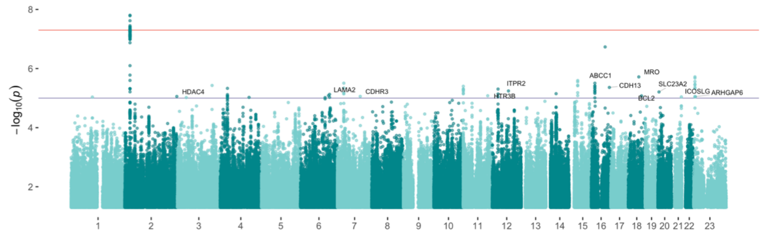

Work/job satisfaction

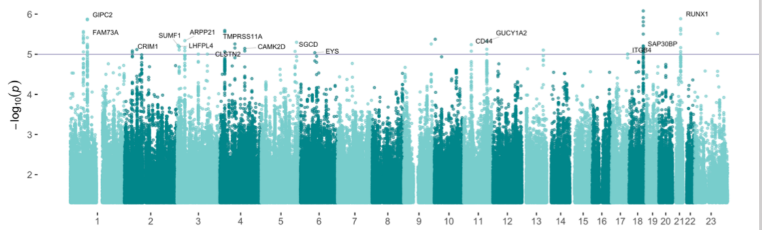

Health satisfaction

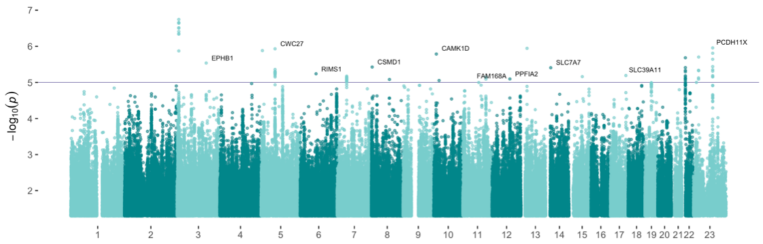

Family relationship satisfaction

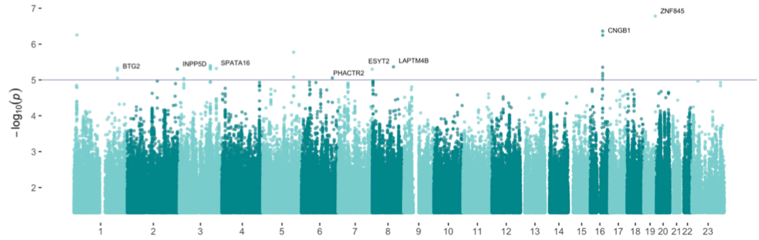

Friendships satisfaction

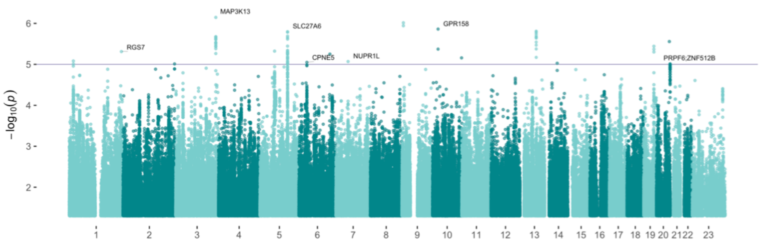

Financial situation satisfaction

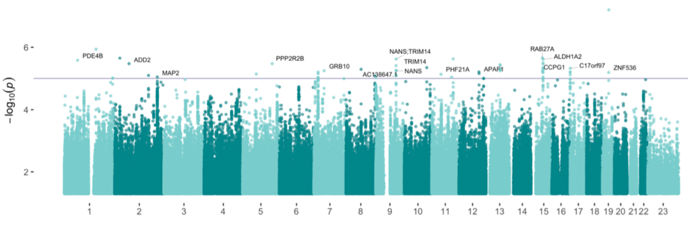

Illness, injury, bereavment, stress in the last 2 years

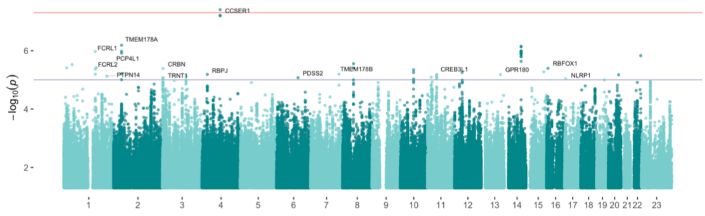

Leisure/social activities

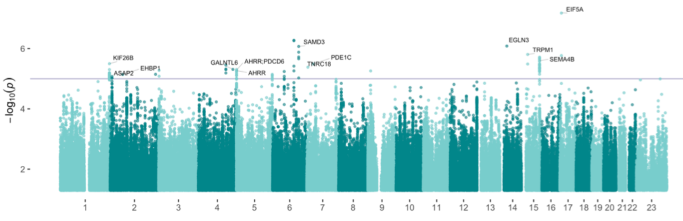

Fluid intelligence score

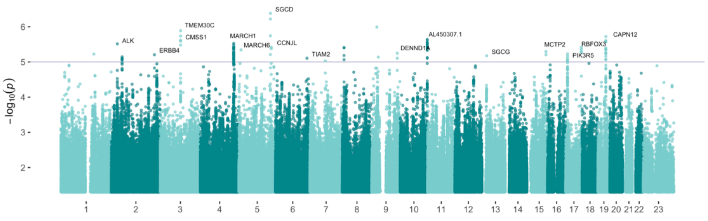

Smoking status

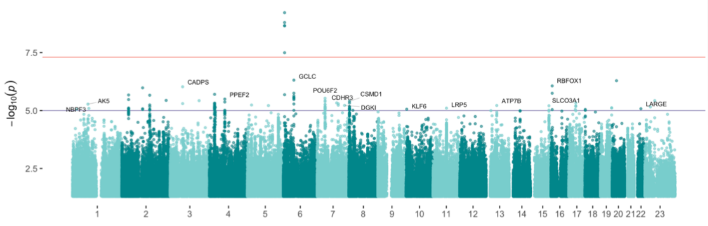

Felt hated by family member as a child

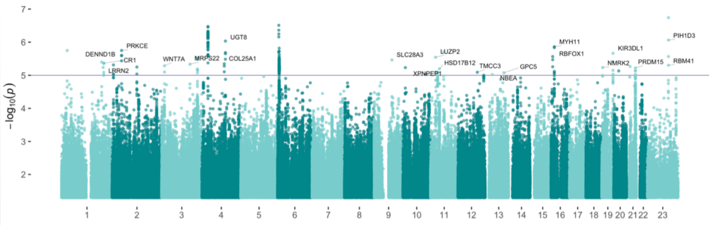

Physically abused by family as a child

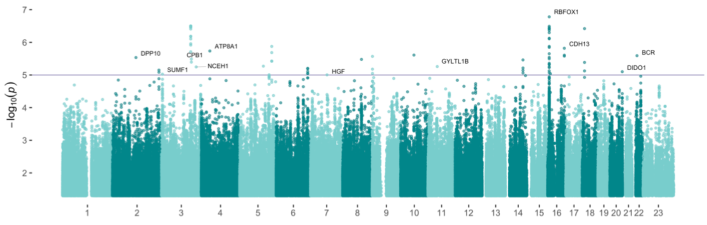

Felt loved as a child

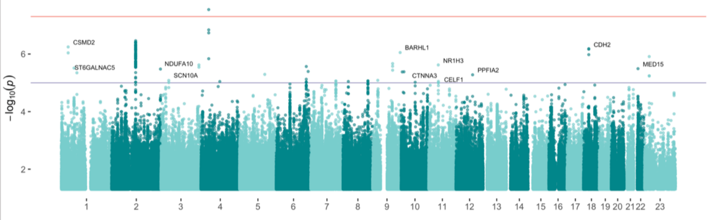

Been in a confiding relationship as an adult

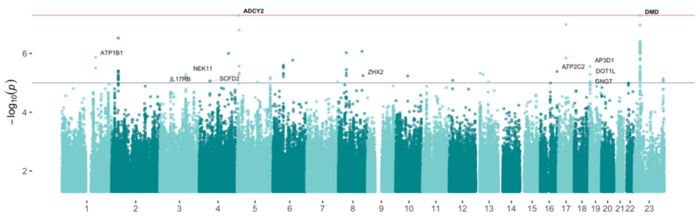

Diagnosed with life-threatening illness

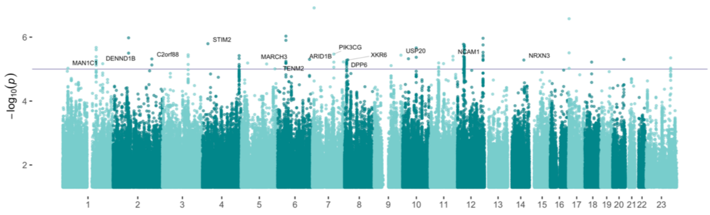

Victim of physically violent crime

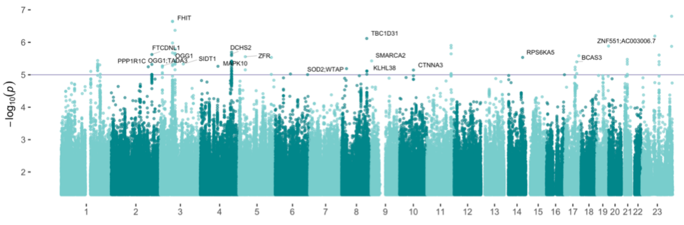

Victim of sexual assault

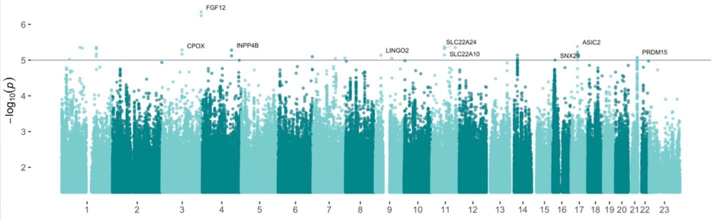

Body mass index (BMI)

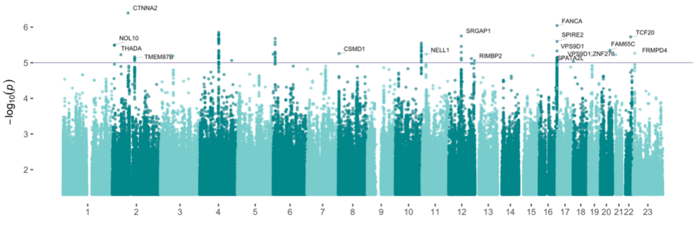

Pain for 3+ months

Gene Analysis

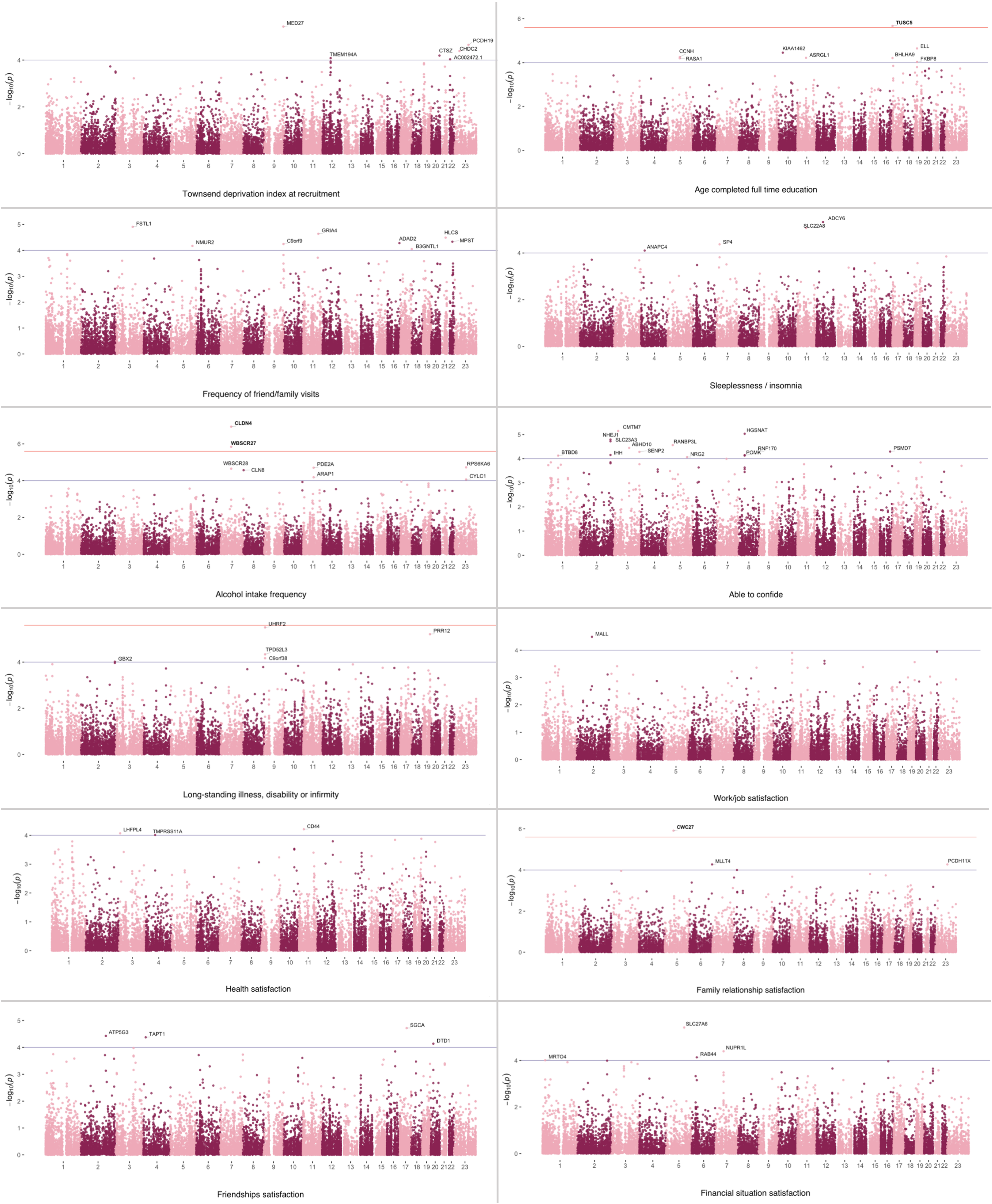

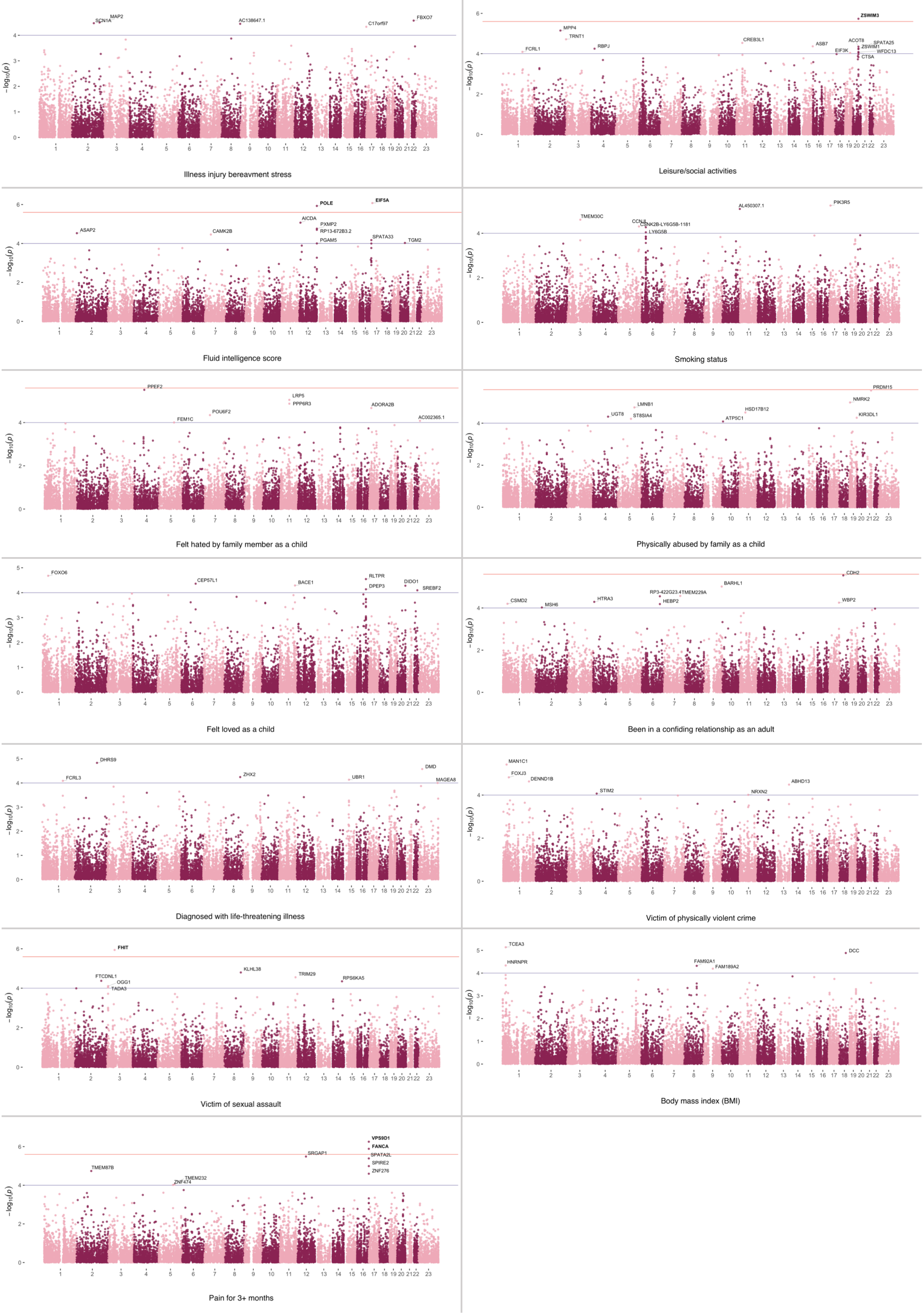

Illness injury bereavment stress
SCN1A
MAP2
AC138647.1
C17orf97
FBXO7
Leisure/social activities
ZSWIM3
MPP4
TRNT1
FCRL1
RBPJ
CREB3L1
ASB7
ACOT8
SPATA25
ZSWIM1
EIF3K
WFDC13
CTSA
Fluid intelligence score
POLE
EIF5A
AICDA
PXMP2
RP13-672B3.2
PGAM5
ASAP2
CAMK2B
SPATA33
TGM2
Smoking status
AL450307.1
PIK3R5
TMEM30C
CCNL1
CSNK2B-LY6G5B-1181
LY6G5B
Felt hated by family member as a child
PPEF2
LRP5
PPP6R3
ADORA2B
POU6F2
FEM1C
AC002365.1
Physically abused by family as a child
PRDM15
NMRK2
LMNB1
UGT8
ST8SIA4
HSD17B12
ATP5C1
KIR3DL1
Felt loved as a child
FOXO6
CEP57L1
BACE1
RLTPR
DPEP3
DIDO1
SREBF2
Been in a confiding relationship as an adult
CDH2
BARHL1
RP3-422G23.4TMEM229A
HEBP2
CSMD2
MSH6
HTRA3
WBP2
Diagnosed with life-threatening illness
DHRS9
FCRL3
ZHX2
UBR1
DMD
MAGEA8
Victim of physically violent crime
MAN1C1
FOXJ3
DENND1B
STIM2
NRXN2
ABHD13
Victim of sexual assault
FHIT
FTCDNL1
OGG1
TADA3
KLHL38
TRIM29
RPS6KA5
Body mass index (BMI)
TCEA3
HNRNPR
FAM92A1
FAM189A2
DCC
Pain for 3+ months
VPS9D1
FANCA
SPATA2L
SPIRE2
ZNF276
SRGAP1
TMEM87B
TMEM232
ZNF474
