# Supplementary material for: Genome-wide gene-environment interactions in neuroticism: an exploratory study across 25 environments
Source: Transl Psychiatry. 2021 Mar 22;11:180. doi: 10.1038/s41398-021-01288-9 (PMC7985503; doi:10.1038/s41398-021-01288-9)
Supplement: Supplementary file 2 — Suppl. Info (A) [file 41398_2021_1288_MOESM2_ESM.pdf]

Supplementary Information (A)

Table of Contents

UK Biobank Sample Information and Quality Control **2**

Heteroscedasticity and Spurious Inflation of GWEIS Test Statistics **4**

QQ-Plots: Robust vs OLS **5**

UK Biobank Sample Information and Quality Control

All data used for this study was obtained from the UK Biobank[1] (www.ukbiobank.ac.uk). The UK Biobank is a large, population-based cohort that includes around 500,000 participants, all of whom have provided written informed consent. The UK Biobank study received ethical approval from the National Research Ethics Service Committee North West Haydock (reference 11/NW/0382), and all procedures were performed in accordance with the World Medical Association Declaration of Helsinki ethical principles for medical research. The current study was conducted under the UK Biobank application number 16406.

Here, we used genotype data from the March 2018 release (version 3), which is an updated version of data released earlier in July 2017. Data collection, primary processing and quality control (QC) was performed by UK Biobank itself, and has been described in detail elsewhere[2]. In short, 489,212 individuals were genotyped on two custom made arrays (UK BiLEVE Axiom^{TM} array & UK Biobank^{TM} Axiom array) sharing 95% of marker content. After quality control, a total of 805,426 unique genotyped markers were imputed by the UK Biobank using two reference panels: The first being a merged reference panel consisting of UK10K haplotypes[3] and the 1,000 Genomes (phase 3)[4] panel; and the second the Haplotype Reference Consortium (HRC)[5] panel. (For any variants imputed in both panels, the HRC imputation was used).

The resulting imputed, quality-controlled data obtained from the UK Biobank consisted of a total of 487,409 individuals and 97,059,328 variants. With this, we performed additional processing and quality control to ensure the inclusion of high-quality variants from unrelated individuals of known ancestry. To do so, we first converted the imputed dosages to hard-called genotypes with PLINK[6] using the default hard call threshold of 0.9. We excluded multi-allelic single nucleotide polymorphisms (SNPs), indels, and SNPs without unique rsID, and applied additional filtering based on missingness (< 5%) and imputation quality (info score > 0.9). Quality control for SNPs on the X chromosome was performed separately, first excluding any SNPs for which there were heterozygous males, or a MAF of < 1e-4. Any SNPs with a difference in MAF or missingness between males and females of > .02 were also removed, as were SNPs with a hardy Weinberg equilibrium of < 5e-8, leaving 268,871 X chromosome SNPs. For autosomal SNPs, we set a minor allele frequency (MAF) threshold of > 1e-5, resulting in 16,703,829 autosomal SNPs.

Ancestry was determined by projecting the autosomal genotype data onto the first 30 principal components of the 1,000 Genomes (phase 3) data using SmartPCA[7]. Each individual was then assigned to the subpopulation of the 1,000 Genomes to which it was closest (as measured by the Mahalanobis distance). For individuals for which this distance was greater than six standard deviations, the ancestry was set to missing, and their samples were excluded (N = 764). Additional sample filtering was performed based on metrics provided by the UK Biobank in the following order: mismatch between reported sex and genetic sex (N = 370 excluded) or sex aneuploidy (N = 471) (both determined via standard examination of X/Y genotype intensities), excluded in kinship inference (N = 8) or excessive number of relatives (N = 189) (for more details, see Bycroft et al.[2]). To obtain a sample of unrelated individuals, we removed samples one at a time, every time removing the person with the greatest number of relatives until no pairs of individuals with a kinship coefficient greater than 0.04 remained. Any individuals who had withdrawn their consent were also removed (N = 118), and altogether these quality control steps resulted in 483,781 individuals.

For this current study, we restricted the sample to individuals with European ancestry (N = 387,558) in order to prevent potential bias from population stratification, and analysed only data from individuals that possessed complete neuroticism data across all items (N = 313,467). We also excluded any variants with a MAF of less than 1%, which left a total of 8,614,007 SNPs for the analyses.

Heteroscedasticity and Spurious Inflation of GWEIS Test Statistics

As noted previously[8,9], GWEIS tests statistics are vulnerable to spurious inflation of test statistics as a result of violations of the assumption of equal variances (i.e. heteroscedasticity). To address this, we computed *t*-statistics for the interaction term using both model-based and robust standard errors in the form of the Huber-White sandwich estimator (see Methods). We evaluated the presence of inflation by obtaining LD-score regression intercepts[10] and plotting the observed -log10 *p*-values against the expected null distribution in the form of QQ-plots for results based on both the model-based and robust standard errors.

In line with previous studies[8,9], we could confirm the presence of mild inflation for several environments when relying on traditional, model-based standard errors. LD-score intercepts[10] for the model-based results ranged from .866 to 1.223, and although these were generally not substantially different from 1, QQ-plots nevertheless indicated diversions from the expected distribution of *p*-values for weakly associated SNPs on several occasions. This was not the case for the robust results, however, for which QQ-plots indicated no signs of inflation and LD-score intercepts were all below 1.03.

All relevant QQ-plots can be found on the following three pages, with LD score intercepts (and standard errors) indicated by β_0.

QQ-Plots: Robust vs OLS

β_0= LD score intercept with standard errors

Townsend deprivation index at recruitment

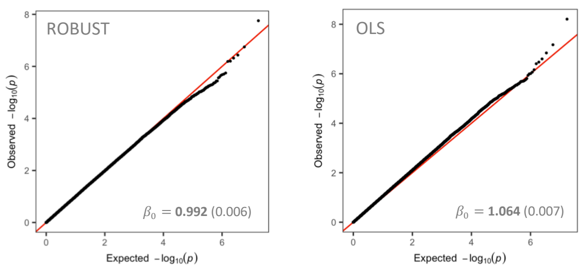

Age completed full time education

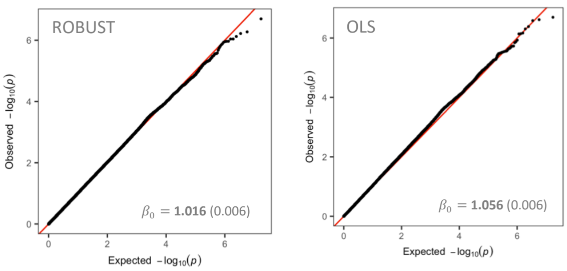

Frequency of friend/family visits

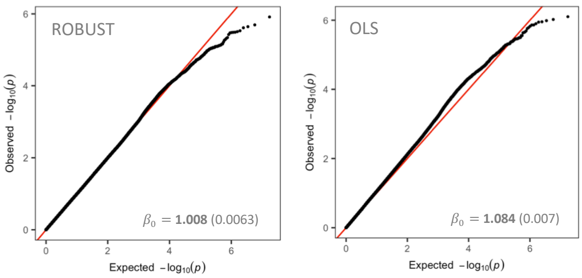

Sleeplessness / insomnia

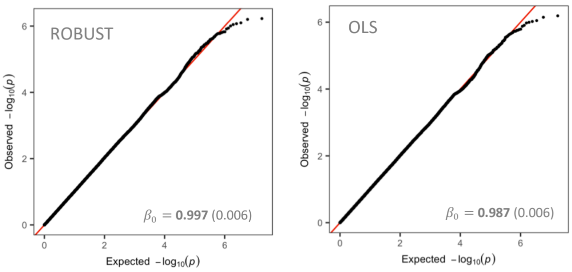

Alcohol intake frequency

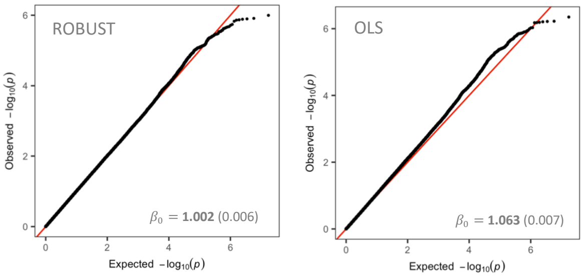

Able to confide

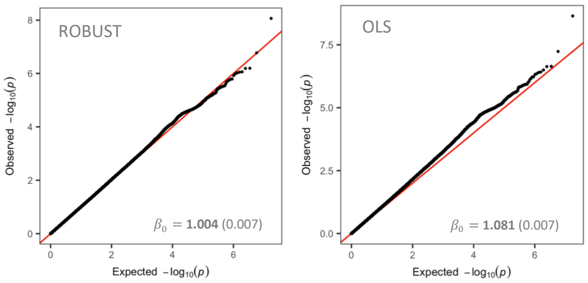

Long-standing illness, disability or infirmity

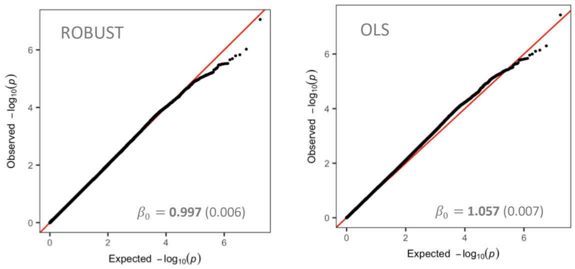

Work/job satisfaction

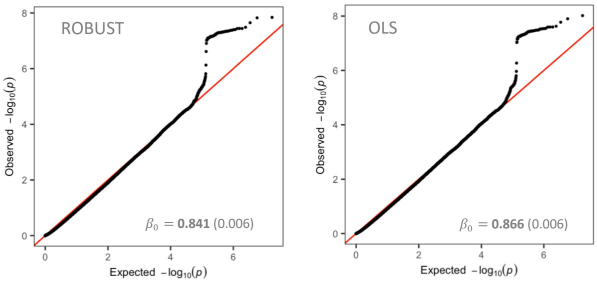

Health satisfaction

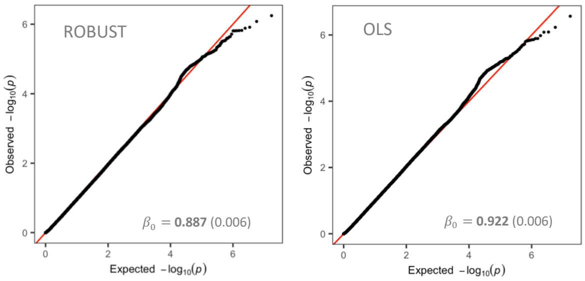

Family relationship satisfaction

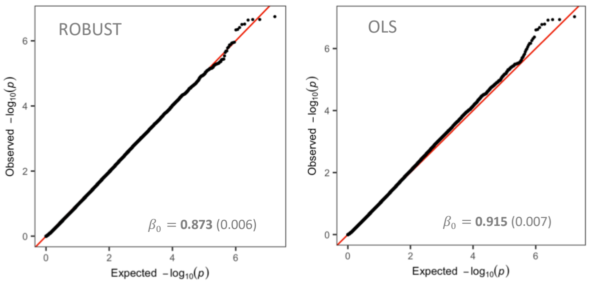

Friendships satisfaction

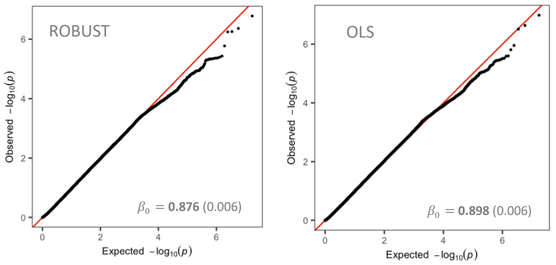

Financial situation satisfaction

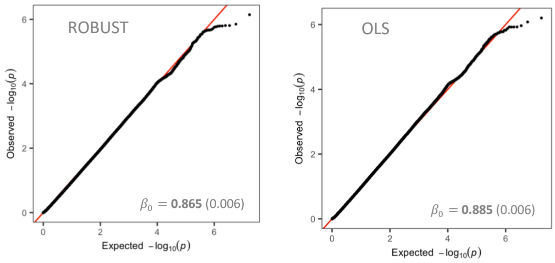

Illness, injury, bereavement, stress in the last 2 years

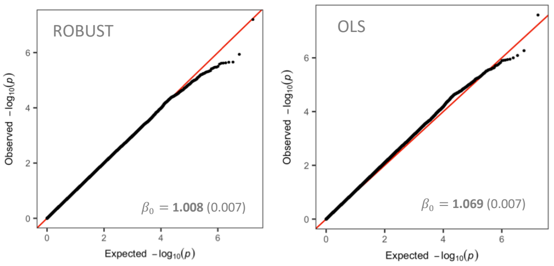

Leisure/social activities

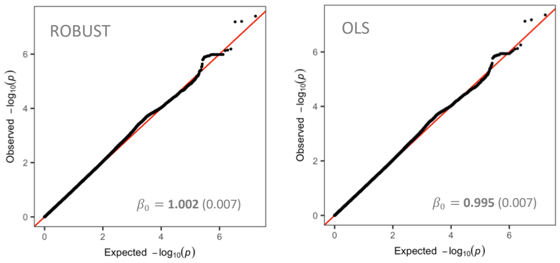

Fluid intelligence score

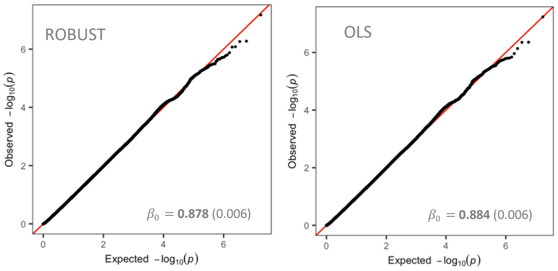

Smoking status

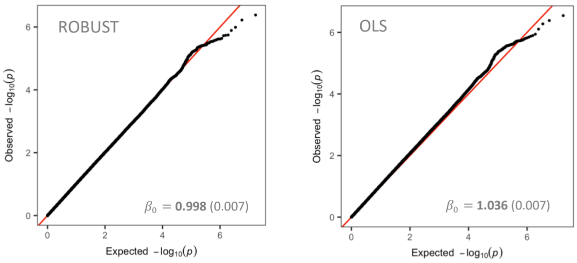

Felt hated by family member as a child

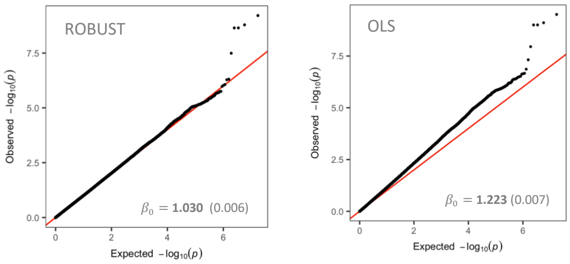

Physically abused by family as a child

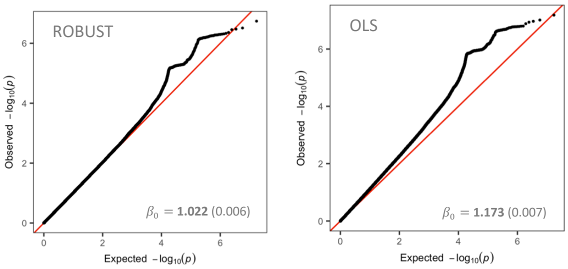

Felt loved as a child

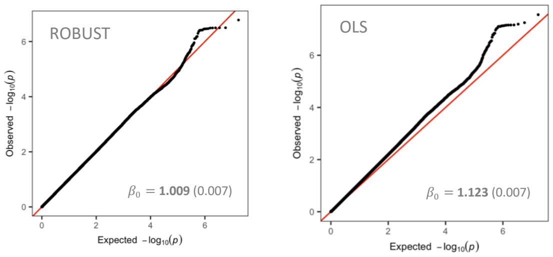

Been in a confiding relationship as an adult

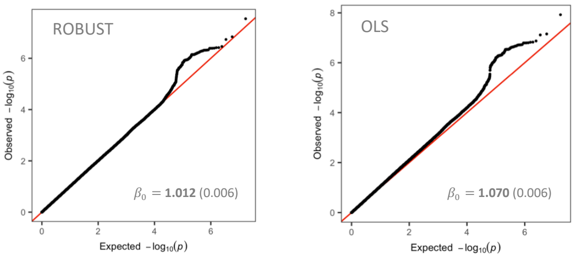

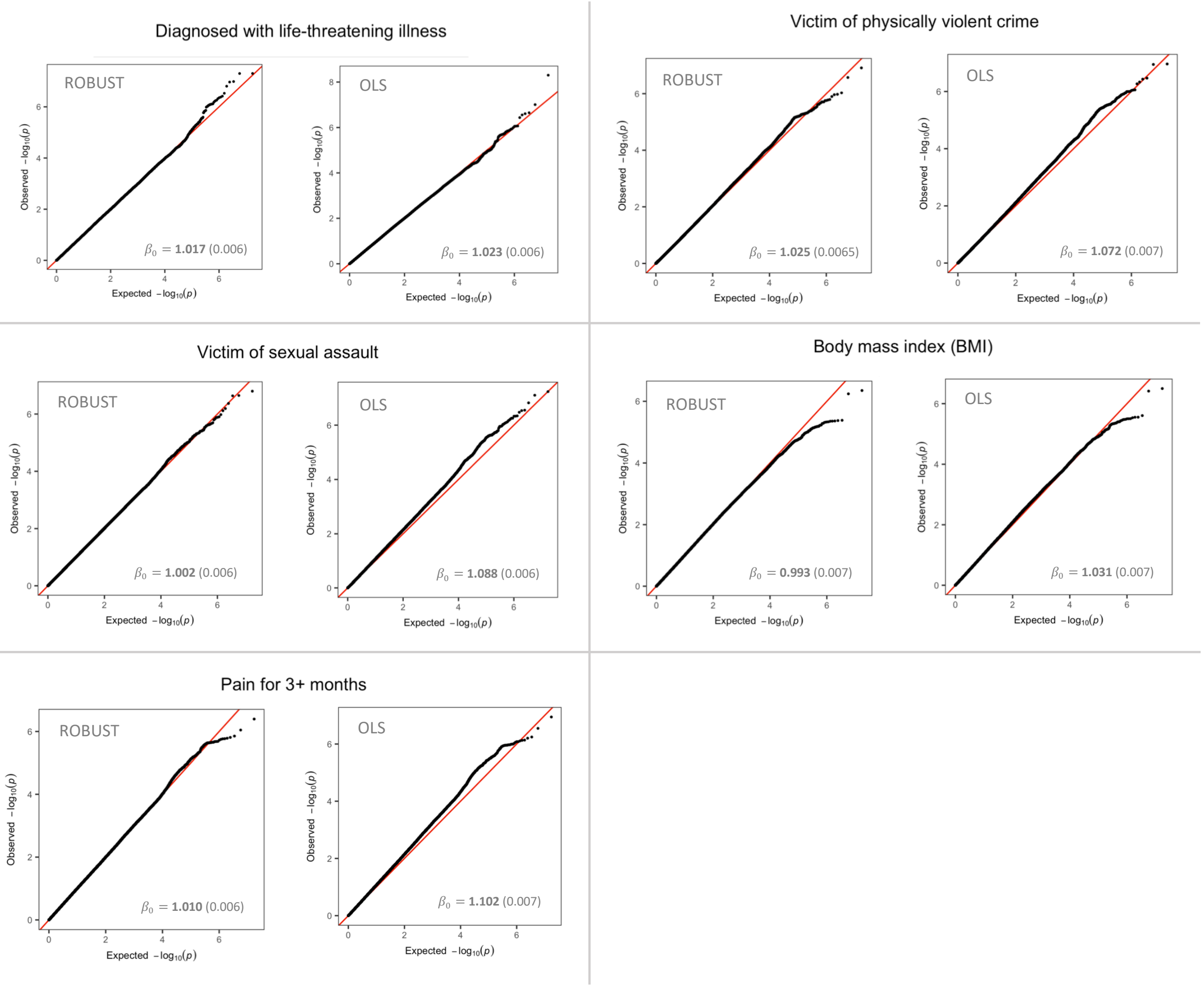
Diagnosed with life-threatening illness
ROBUST
β_0 = 1.017 (0.006)
OLS
β_0 = 1.023 (0.006)
Victim of physically violent crime
ROBUST
β_0 = 1.025 (0.0065)
OLS
β_0 = 1.072 (0.007)
Victim of sexual assault
ROBUST
β_0 = 1.002 (0.006)
OLS
β_0 = 1.088 (0.006)
Body mass index (BMI)
ROBUST
β_0 = 0.993 (0.007)
OLS
β_0 = 1.031 (0.007)
Pain for 3+ months
ROBUST
β_0 = 1.010 (0.006)
OLS
β_0 = 1.102 (0.007)
Observed $-\log_{10}(p)$
Expected $-\log_{10}(p)$

REFERENCES

1. Sudlow, C. *et al.* UK Biobank: An Open Access Resource for Identifying the Causes of a Wide Range of Complex Diseases of Middle and Old Age. *PLOS Med.* **12**, e1001779 (2015).
2. Bycroft, C. *et al.* The UK Biobank resource with deep phenotyping and genomic data. *Nature* **562**, 203–209 (2018).
3. The UK10K Consortium. The UK10K project identifies rare variants in health and disease. *Nature* **526**, 82–90 (2015).
4. The 1000 Genomes Project Consortium. A global reference for human genetic variation. *Nature* **526**, 68–74 (2015).
5. McCarthy, S. *et al.* A reference panel of 64,976 haplotypes for genotype imputation. *Nat. Genet.* **48**, 1279–1283 (2016).
6. Chang, C. C. *et al.* Second-generation PLINK: rising to the challenge of larger and richer datasets. *Gigascience* **4**, 7 (2015).
7. Patterson, N., Price, A. L. & Reich, D. Population Structure and Eigenanalysis. *PLoS Genet.* **2**, e190 (2006).
8. Voorman, A., Lumley, T., McKnight, B. & Rice, K. Behavior of QQ-plots and Genomic Control in studies of gene-environment interaction. *PLoS One* **6**, e19416 (2011).
9. Almli, L. M. *et al.* Correcting systematic inflation in genetic association tests that consider interaction effects: application to a genome-wide association study of posttraumatic stress disorder. *JAMA Psychiatry* **71**, 1392–1399 (2014).
10. Bulik-Sullivan, B. K. *et al.* LD Score Regression Distinguishes Confounding from Polygenicity in Genome-Wide Association Studies. *Nat. Genet.* **47**, 291–295 (2015).
